# LC/MS Q-TOF Metabolomic Investigation of Amino Acids and Dipeptides in *Pleurotus ostreatus* Grown on Different Substrates

**DOI:** 10.1021/acs.jafc.2c04197

**Published:** 2022-08-09

**Authors:** Roberto Maria Pellegrino, Francesca Blasi, Paola Angelini, Federica Ianni, Husam B. R. Alabed, Carla Emiliani, Roberto Venanzoni, Lina Cossignani

**Affiliations:** † Department of Chemistry, Biology and Biotechnology, University of Perugia, 06122 Perugia, Italy; ‡ Department of Pharmaceutical Sciences, University of Perugia, 06126 Perugia, Italy; § Center for Perinatal and Reproductive Medicine, Santa Maria della Misericordia University Hospital, University of Perugia, Sant’Andrea delle Fratte, 06132 Perugia, Italy

**Keywords:** untargeted metabolomics profiling, Pleurotus ostreatus mushroom, polar metabolites, data analysis, multivariate statistical analysis, functional ingredients

## Abstract

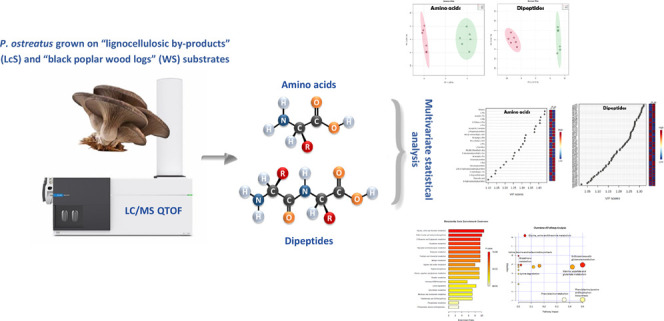

The well-established correlation between diet and health arouses great interest in seeking new health-promoting functional foods that may contribute to improving health and well-being. Herein, the metabolomic investigation of *Pleurotus ostreatus* samples grown on two different substrates (black poplar wood logs, WS, and lignocellulosic byproducts, LcS) revealed the high potential of such a mushroom as a source of bioactive species. The liquid chromatography/mass spectrometry combined with quadrupole time-of-flight (LC/MS Q-TOF) analysis allowed the identification of essential and nonessential amino acids along with the outstanding presence of dipeptides. Multivariate statistical models highlighted important differences in the expression of both classes of compounds arising from the growth of *P. ostreatus* strains on WS and LcS. The former, in particular, was correlated to an increased expression of carnitine-based amino acid derivatives and proline-based dipeptides. This finding may represent a potential strategy to drive the expression of bioactive compounds of interest to obtain enriched mushrooms or useful functional ingredients from them.

## Introduction

Advances in food metabolomics are emerging in the scientific panorama as a pivotal tool applicable to different aspects of food science including food safety and quality, microbiology, processing, functional foods, and nutrition. The phenotypic determination of a food product allows mapping specific pathways to get a comprehensive characterization of the food molecular composition. This, in turn, on the one side, facilitates the detection of adulteration or changes in the nutritional profile and, on the other, would enable the selection of new potential biomarkers associated with the intake of a specific nutrient. Therefore, the identification of peculiar interactions and components in a food matrix plays a significant role in the attribution of the final properties of the product itself (i.e., sensory attributes, food authenticity, nutritional quality, and safety) as well as to establish a potential impact on the health status. Metabolomics research has been growing, especially in the food science sector, for the evaluation and identification of markers of food quality, processing, and microbiology. Accordingly, metabolomics analyses have been applied to several matrices such as honey, wine, meat, fruits, vegetables, and mushrooms to cite but a few.^1,2^ In this context, special attention has been drawn in recent years to the study of mushrooms whose chemical composition and associated benefits are marking them increasingly attention-grabbing species. Furthermore, in a wider frame directed to a circular economy perspective, a great potential is associated with the use of fungal biotechnology in finding sustainable solutions to produce sources of food, feed, chemicals, and diverse materials.^3^ In a recent study, Meyer and co-workers emphasized the ability of fungi to selectively transform organic materials into a wide pool of useful products, highlighting how their proper exploitation could help face many future challenges besides representing an exceptional reservoir of healthy compounds.^4^ In accordance, the potential health-promoting and therapeutic properties of mushrooms are strictly associated with high contents of various mycochemicals among which proteins and peptides, polysaccharides, unsaturated fatty acids, minerals, and secondary metabolites stand out.^5^

In particular, the role of dietary proteins has gained increasing acknowledgment not only as basic molecules needed for the growth and maintenance of physiological functions but also as a source of amino acids and small bioactive peptides (BPs). Concerning proteins in mushrooms, their higher nutritional value with respect to most plant proteins relates to the presence of all essential amino acids required by humans.^6^ For this reason, since the quality, quantity, and availability (*in vivo*) of proteins in terms of free and essential amino acids are reliable indicators of the nutritional value of mushrooms, the determination of free amino acid composition might be of great value for potential applications in fields such as pharmaceutical, medical, food, and nutritional sciences.^7^

Sun and co-workers^8^ reported an average value of free amino acids of 4345 mg/100 g dry weight (DW), measured in 13 different mushroom species. The content of essential amino acids, in the analyzed species, was 1033.4 mg/100 g DW. The importance of identifying the amino acid profile varies from the role in body protein synthesis to the association with significant health-related effects being involved in various cellular pathways, gene expression, oxidative stress, intracellular protein metabolism, and immune processes.^9,10^ Likewise, the study of BPs is constantly attracting remarkable attention from the scientific community for the physiological effects exerted on the human body. BPs are generally sequences between 2 and 20 amino acids, encrypted in the parent protein sequence and activated once released. Their release could follow proteolytic processes such as hydrolysis by proteolytic enzymes derived from microorganisms or plants, microbial fermentation, gastrointestinal digestion, or processing conditions.^11^ Several food-derived BPs showing antihypertensive, antioxidant, immunomodulatory, and antibacterial activities among others have been identified. In particular, small di- or tripeptides present one main advantage over longer ones in that they are orally active owing to the higher stability and ability to penetrate biological barriers.

In particular, one main advantage of di- and tripeptides, over longer ones, is their improved activity by oral administration owing to the higher stability and ability to penetrate biological barriers. For these reasons, BPs are currently witnessing a growing success in a broad range of applications as drugs, nutraceuticals, or functional foods for promoting health. Recently, Zhou and co-workers reported an average protein content in different mushrooms of 23.80 g ± 9.82 g/100 g DW^12^ whose quality makes such species suitable as a dietary food supplement. Since proteins also serve as a precursor material to isolate potential bioactive peptides (BPs), their high levels outline mushrooms as a promising source of BPs, which add up to the endogenous peptides secreted to survive in changing environments. Several biological activities, including antimicrobial, antifungal, antihypertensive, antioxidant, and anticancer, have been attributed to various mushroom peptides.^12^

Noteworthy, the presence of peptides, together with free amino acids, plays an important role in eliciting organoleptic characteristics of mushrooms, thus underlying their contribution to food palatability.^13,14^

In light of all of the above, and based on our recent works,^15,16^ we focused our attention on the genus *Pleurotus* and, specifically, on the *Pleurotus ostreatus* species characterized by a high economic significance. *P. ostreatus*, also known as oyster mushroom, is, in fact, one of the most globally cultivated and affordable species, normally widespread in nature, nutritionally rich, and with a high content of bioactive compounds.

Considering the interest in *P. ostreatus* for human consumption, and within a wider project aimed at investigating the impact of the basal substrate composition on the metabolic profile, the main objective of this study was the identification of polar metabolites, specifically amino acids and peptides, in *P. ostreatus* samples by applying liquid chromatography/mass spectrometry combined with quadrupole time-of-flight (LC/MS Q-TOF). Remarkably, our work highlighted the presence of a number of dipeptides along with the well-known presence of essential and nonessential amino acids. To the best of our knowledge, the presence of dipeptides has never been reported so far; therefore, the present paper represents the first case in which such low-molecular-mass species have been identified in *P. ostreatus* samples. Multivariate statistical models were also applied to highlight any difference arising from the growth of *P. ostreatus* strains on two different substrates. Overall, this study could represent a valuable tool to allow a deep evaluation of the nutritional profile of such wild edible mushrooms.

## Materials and Methods

### Chemicals and Reagents

LC-MS grade water and methanol, heptafluoro butanoic acid (HFBA), and ammonium acetate were purchased from Sigma-Aldrich (Sigma-Aldrich GmbH, Hamburg, Germany).

### Samples

The *P. ostreatus* fruiting bodies were collected in Piegaro (Perugia, Umbria, Italy) in November 2020. The Vaucher specimen (PeruMyc2256) was identified based on the morphological and molecular analyses and was deposited in the herbarium at the University of Perugia (Department of Chemistry, Biology and Biotechnology (DCBB)). In brief, to isolate mycelium in pure culture, small pieces of pseudotissue (about 10 mm^3^) were aseptically drawn from the fruiting bodies and inoculated into Rose Bengal chloramphenicol agar.^17^ The cultures were incubated in the dark at 25 °C for 14 days. Subsequently, they were regularly subcultured on malt extract agar.^18^ Once the mycelium completely invaded the agar medium, the cultures were used for spawn preparation.^19^

Two different substrates were tested for the cultivation of *P. ostreatus* strains, “black poplar wood logs” (WS) and “lignocellulosic byproducts” (LcS), as previously reported.^15^ In particular, the first substrate (WS) was represented by wood logs of *Populus nigra* L., while the second one (LcS) had the following composition: wheat straw (40%), *Hordeum vulgare* caryopsis (20%), *Triticum dicoccum* caryopsis (20%), and black poplar sawdust (20%). *P. ostreatus* samples were grown in triplicate on the two substrates described. In the end, 12 different samples were collected and then freeze-dried. Specifically, six *P. ostreatus* samples were grown on the WS substrate, while the remaining six samples were grown on the LcS substrate.

### Polar Metabolite Extraction

*P. ostreatus* samples were obtained from 4–5 fruiting bodies for each strain from the first fruiting flush (stored at −80 °C until use) and then lyophilized.

The polar metabolite extraction of freeze-dried samples was carried out by mixing 50 mg of sample with 1 mL of ultrapure water. After vortexing, the samples were placed in an ultrasonic bath and processed for 10 min. The samples were then centrifuged for 10 min at 14 000 g at 20 °C, and then 300 μL of cold methanol (−20 °C) was added. The mixture was further vortexed for 10 s and centrifuged for 10 min at 14 000 g at 4 °C. The supernatant was filtered through 0.2 μm nylon membrane filters. Extracts were immediately transferred to an autosampler vial and analyzed without further treatment by injecting 5 μL each run.

### Untargeted LC-MS/MS-Based Metabolomics Analysis

Untargeted metabolomics was carried out using ultraperformance liquid chromatography mass spectrometry (UHPLC)-Q-TOF, employing a 1260 ultra-high-performance liquid chromatograph and a G6530A Q-TOF mass spectrometer equipped with a JetStream source (both Agilent Technologies, Santa Clara, CA). Chromatographic separation was performed on an Ascentis Express RP-Amide column (150 mm × 3 mm, 2.7 μm, Supelco). The mobile phase consisted of 0.2% aqueous solution of HFBA (A) and 10 mM ammonium acetate methanolic solution (B). Mobile phase was delivered at a flow rate of 0.6 mL/min under the following gradient procedure: 0–2.5 min, 3% B; 2.5–5 min, 3–20% B; 5–7.5 min, 20% B; 7.5–13 min, 20–55% B; 13–15.5 min, 55–95% B; 15.5–18.5 min, 95% B; 18.5–19 min, 3% B; 22 min stop run. The column temperature was set at 35 °C, and the injection volume was 5 μL. The source was operated in both polarities as follows: ion spray 3500 V; gas temperature and sheath gas temperature were set at 250 and 300 °C respectively; nebulizer (N_2_) 35 psi; and sheath gas flow 12 L/min. Data-dependent acquisition was used in the mass range of 40–1700 *m*/*z* for both MS and MS/MS with a collision energy of 30 V.

Raw data was processed with MS-DIAL (version 4.48)^20^ to perform peak detection, peak alignments, peak area integration of the MS signal, and metabolite annotation. Annotation based on MS and MS/MS data was performed using the NIST 2020 tandem mass library. All metabolites with a total score greater than 60% were considered. Two data sets, one for each polarity, were obtained and then merged in a single table (Table 1S Supporting Information). At the end of the analytical workflow, a data matrix reporting the relative abundances expressed as the area of each annotated peak in each sample was obtained. This data matrix was used for statistical analysis and pathway analysis, as described below.

### Statistical Analysis and Pathway Analysis

Principal component analysis (PCA), partial least-squares data analysis (PLS-DA), heat map, and metabolic pathway enrichment analysis were performed with MetaboAnalyst.^21^ For statistical analysis, samples were normalized by median, followed by Pareto scaling.

## Results and Discussion

The fruiting bodies of oyster mushrooms are well recognized for their high nutritional value, and, more specifically, various species belonging to the *Pleurotus* genus have been characterized as sources of substances with multidirectional health-promoting properties.^22^ More in detail, *P. ostreatus* stands out for its richness in bioactive components, which confer to this specialty mushroom a valuable nutritional and medicinal value.

The outcomes obtained in our recent works, based on the evaluation of the antioxidant and antimicrobial properties^15^ and on the characterization of the lipid fraction in *P. ostreatus* samples,^23^ highlighted a significant influence of the growth substrate on the *in vitro* measured activity and on the lipidomic profile, respectively. Based on these considerations, the main objective of the present investigation was to extend the attention toward the analysis of polar metabolites. Twelve *P. ostreatus* samples were overall analyzed, six of which were grown on the WS substrate, while six were grown on the LcS substrate (Table 2S Supporting Information). A deeper insight into the results provided by the untargeted metabolomics approach *via* LC/MS Q-TOF allowed the detection, among the various compounds, of amino acids along with the outstanding presence of dipeptides never described, to the best of our knowledge, so far. Concerning protein composition, the occurrence of all essential and nonessential free amino acids was found in the referred mushroom species (Table 1). In line with our findings, literature data reports a mean value of high-quality proteins of 28.85% in fresh *P. ostreatus* samples.^24^ In a recent work, Tagkouli and co-workers^9^ estimated, on average, a crude protein content in *P. ostreatus* mushrooms (ranging between 164.07 ± 1.65 and 177.36 ± 3.55 mg/g) higher than in other *Pleurotus* species. The same authors also found the highest content of free amino acids exhibited by *P. ostreatus*, although wide value fluctuations were observed with respect to literature data.^25−27^ However, since several factors (i.e., genetic variability, harvest treatments, and type of growth substrates) could affect mushroom composition, such fluctuations are expectable.

**Table 1:** Identified Amino Acids and Relative Abundance Measured as % Area Values ± Standard Error (Mean % Area ± SE, *n* = 6) in the *P. ostreatus* Samples Grown on WS and LcS Substrates

identified amino acid (AA)		% area value		identified amino acid (AA)		% area value	
#	metabolite	WS substrate	LcS substrate	#	metabolite	WS substrate	LcS substrate
AA-1	*N*-cyclopentylglycine	0.00 ± 0.00	0.05 ± 0.02	AA-28	ornithine	0.53 ± 0.06	1.27 ± 0.30
AA-2	cycloleucine	0.01 ± 0.00	0.03 ± 0.01	AA-29	*N*-acetyltyrosine	0.53 ± 0.02	0.48 ± 0.01
AA-3	2-chlorophenylalanine	0.01 ± 0.00	0.01 ± 0.00	AA-30	3-hydroxyisovaleroylcarnitine	0.58 ± 0.05	0.38 ± 0.03
AA-4	decanoylcarnitine	0.01 ± 0.00	0.01 ± 0.00	AA-31	NG,NG-dimethyl-arginine	0.52 ± 0.01	0.71 ± 0.01
AA-5	1-acetylproline	0.01 ± 0.00	0.03 ± 0.01	AA-32	ergothioneine	0.68 ± 0.05	0.79 ± 0.08
AA-6	*N*-acetylphenylalanine	0.01 ± 0.00	0.02 ± 0.00	AA-33	3-hydroxybutyrylcarnitine	1.39 ± 0.18	0.64 ± 0.13
AA-7	*N*-(aminocarbonyl)-phenylalanine	0.03 ± 0.00	0.02 ± 0.00	AA-34	*O*-*tert*-butyl-thr	1.08 ± 0.04	0.74 ± 0.01
AA-8	threonine	0.01 ± 0.00	0.06 ± 0.00	AA-35	glutamic acid	1.59 ± 0.08	0.86 ± 0.02
AA-9	acetamidomethyl-cysteine	0.01 ± 0.00	0.04 ± 0.01	AA-36	methionine	2.41 ± 0.36	2.16 ± 0.05
AA-10	*N*,*N*-dimethyl-histidine	0.01 ± 0.00	0.02 ± 0.00	AA-37	proline	2.08 ± 0.07	4.20 ± 0.17
AA-11	*N*-acetylproline	0.01 ± 0.00	0.02 ± 0.00	AA-38	*N*-formyltryptophan	2.79 ± 0.11	2.45 ± 0.08
AA-12	arginine, methyl ester	0.03 ± 0.00	0.04 ± 0.00	AA-39	valine	2.70 ± 0.11	2.57 ± 0.09
AA-13	β-homolysine	0.03 ± 0.01	0.15 ± 0.06	AA-40	carnitine	2.79 ± 0.21	1.00 ± 0.10
AA-14	acetylthreonine	0.02 ± 0.00	0.13 ± 0.01	AA-41	lysine	3.00 ± 0.15	3.28 ± 0.20
AA-15	hexanoylcarnitine	0.04 ± 0.01	0.06 ± 0.00	AA-42	propionylcarnitine	3.17 ± 0.11	1.27 ± 0.15
AA-16	octanoylcarnitine	0.07 ± 0.01	0.03 ± 0.00	AA-43	glutamine	2.79 ± 0.15	2.44 ± 0.23
AA-17	hydroxyarginine	0.06 ± 0.01	0.02 ± 0.00	AA-44	tyrosine	4.01 ± 0.17	3.56 ± 0.10
AA-18	norvaline	0.07 ± 0.00	0.04 ± 0.00	AA-45	tryptophan	4.14 ± 0.15	3.93 ± 0.12
AA-19	phenylalanine, methyl ester	0.08 ± 0.00	0.04 ± 0.00	AA-46	isoleucine	5.46 ± 0.36	4.83 ± 0.19
AA-20	homoserine	0.08 ± 0.00	0.11 ± 0.01	AA-47	histidine	5.02 ± 0.19	4.90 ± 0.06
AA-21	2-methylbutyrylcarnitine	0.09 ± 0.01	0.09 ± 0.00	AA-48	leucine	7.62 ± 0.42	6.39 ± 0.23
AA-22	aspartic acid	0.09 ± 0.01	0.02 ± 0.00	AA-49	acetylcarnitine	7.99 ± 0.74	3.61 ± 0.08
AA-23	betaine	0.25 ± 0.07	4.66 ± 0.20	AA-50	arginine	7.74 ± 0.21	8.80 ± 0.35
AA-24	N5-(1-iminoethyl)-ornithine	0.23 ± 0.01	0.13 ± 0.01	AA-51	phenylalanine	9.53 ± 0.31	8.95 ± 0.29
AA-25	*N*-α-acetyl-ornithine	0.17 ± 0.01	0.31 ± 0.02	AA-52	butyrylcarnitine	6.33 ± 1.00	10.47 ± 0.62
AA-26	pipecolic acid	0.37 ± 0.02	0.21 ± 0.03	AA-53	isovalerylcarnitine	11.39 ± 1.80	12.60 ± 0.52
AA-27	*N*-α-(*tert*-butoxycarbonyl)-histidine	0.36 ± 0.03	0.36 ± 0.00				

Furthermore, nonproteinogenic, structurally diverse, amino acids were detected, having their origin in the canonical amino acids or as products of biosynthetic pathways not plainly identified.^28^ Among these, for example, dialkylated or hydroxylated α-amino acids,^29^ the unbranched isomer of valine or leucine,^28^ carnitine, and its acyl-derivatives^30^ stand out. Some of noncanonical amino acids isolated from fungi are incorporated into toxins or antibiotics and have a role in protecting the host plant against infections or are, more generally, biologically active substances particularly appealing for numerous medical and pharmaceutical applications.

**Figure 1:**
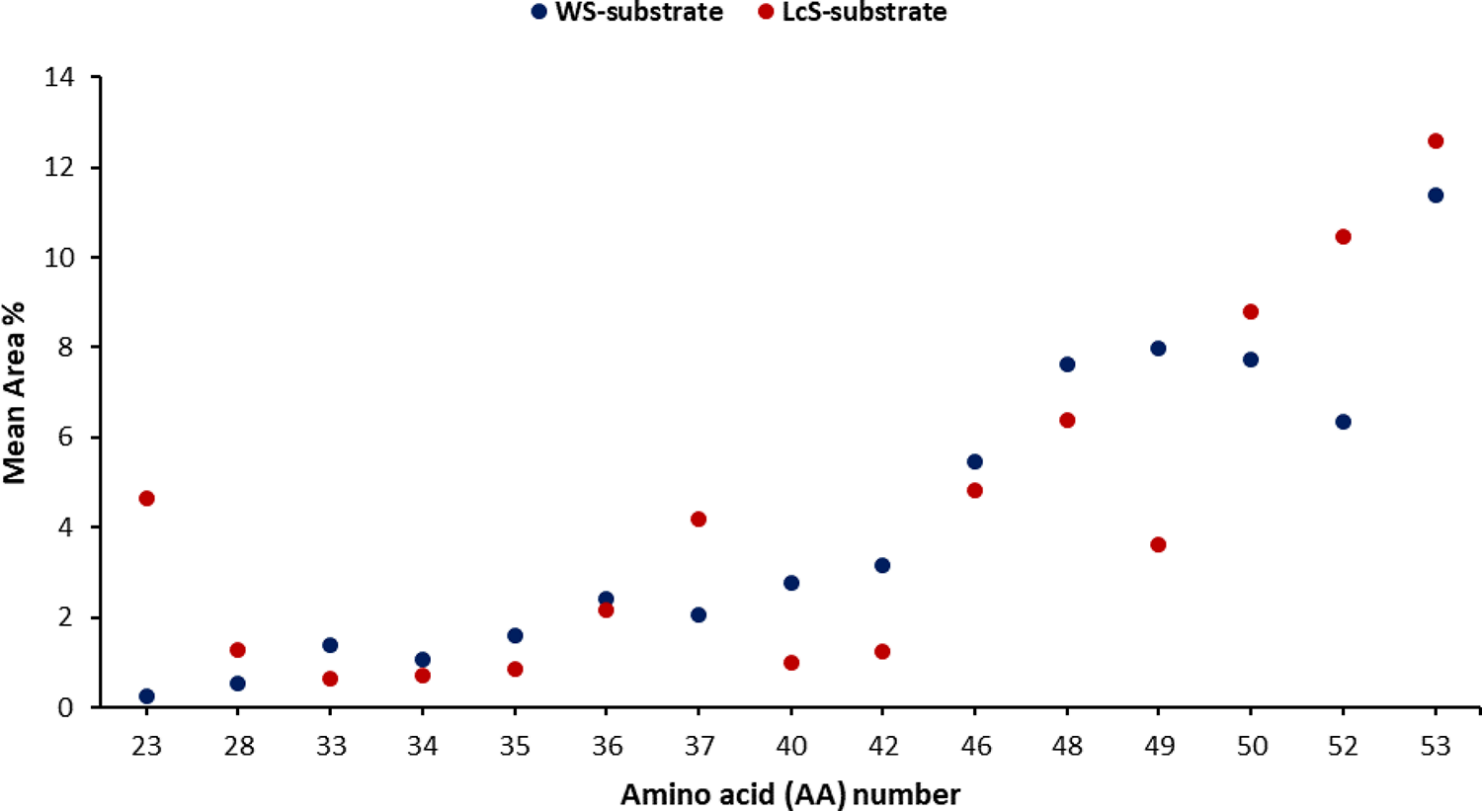
Normalized distribution (area values) of the most differentially expressed amino acid based on the comparison between the two growth substrates (WS and LcS). Points represent *P. ostreatus* strains grown on the respective substrate and are reported as mean values (*n* = 6). The corresponding number for each amino acid is defined in Table 1.

Abundance rank data for amino acids, depicted in Figure 1 and detailed in Table 1, evidenced a different expression of such metabolites in the investigated samples. Generally, aromatic, basic, and some hydrophobic natural and unnatural amino acids were the most abundant in the investigated samples (area values > 1.0%).

More specifically, by taking into account the influence of the growth substrate, the WS well correlated to increased levels of 3-hydroxybutyrylcarnitine (33), glutamic acid (35), carnitine (40), propionylcarnitine (42), leucine (48), and acetylcarnitine (49), while the LcS was mainly effective in the increased expression of betaine (23), ornithine (28), proline (37), and butyrylcarnitine (52) among the others (Figure 1 and Table 1).

In addition, as anticipated above, the investigated *P. ostreatus* samples, grown on WS or LcS substrates, represented a plentiful resource of dipeptides with potential bioactive activities and beneficial effects on human health (Table 2). Generally, mushroom proteins have high thermal and pH stability;^31^ therefore, since in our experiments no drastic extraction conditions were adopted, the recovery of such dipeptides in the extracts could be reasonably ascribed to their endogenous presence as secondary functional metabolites or natural intermediate products of protein catabolism. There is in general a dearth of information on the origin and presence of dipeptides in mushrooms. Moore and co-workers^32^ recently described pyroglutamyl dipeptides from hydrolyzed mushroom proteins, formed by an intramolecular cyclization of glutamine or glutamic acid, with saltiness-enhancing potential. Extracts or hydrolysates of mushrooms were also found to exert a blood pressure lowering effect, and some active dipeptides as Ile–Tyr, Ile–Trp, and Lys–Trp were identified.^33^ However, apart from the possibility of obtaining such peptides in digested or processed edible mushrooms, their endogenous occurrence has not been thoroughly studied and hence further investigation would be needed to fill these gaps.

**Table 2:** Identified Peptides and Relative Abundance Measured as % Area Values ± Standard Error (Mean % Area ± SE, *n* = 6) in the *P. ostreatus* Samples Grown on WS and LcS Substrates

identified dipeptide (pept)		% area value		identified dipeptide (pept)		% area value	
#	metabolite	WS substrate	LcS substrate	#	metabolite	WS substrate	LcS substrate
Pept-1	His–Trp	0.00 ± 0.00	0.13 ± 0.01	Pept-61	Ala–His	0.61 ± 0.03	0.35 ± 0.01
Pept-2^a^	Pro–Ala–Arg	0.01 ± 0.00	0.03 ± 0.01	Pept-62	Gln–Val	0.44 ± 0.02	0.49 ± 0.01
Pept-3	His–Ile	0.04 ± 0.01	0.91 ± 0.06	Pept-63	Thr–Thr	0.31 ± 0.05	0.33 ± 0.04
Pept-4	His–Val	0.09 ± 0.04	1.23 ± 0.07	Pept-64	Gly–Val	0.46 ± 0.03	0.92 ± 0.03
Pept-5	Leu–His	0.04 ± 0.01	0.25 ± 0.04	Pept-65	Tyr–Pro	0.66 ± 0.03	0.24 ± 0.01
Pept-6	Pro–Tyr	0.11 ± 0.03	0.26 ± 0.02	Pept-66	Lys–Ser	0.43 ± 0.04	0.84 ± 0.05
Pept-7	Leu–Ser	0.04 ± 0.00	0.04 ± 0.00	Pept-67	Glu–Val	0.46 ± 0.03	0.60 ± 0.01
Pept-8	His–Lys	0.06 ± 0.01	0.27 ± 0.01	Pept-68	g-Glu–Glu	0.55 ± 0.01	0.52 ± 0.03
Pept-9	Met–Gly	0.05 ± 0.00	0.03 ± 0.00	Pept-69	Gln–Gln	0.80 ± 0.07	0.50 ± 0.01
Pept-10	His–His	0.09 ± 0.01	0.22 ± 0.02	Pept-70	Lys–Asp	0.58 ± 0.02	0.88 ± 0.03
Pept-11	Val–Ser	0.07 ± 0.01	0.14 ± 0.03	Pept-71	Thr–Lys	0.50 ± 0.03	0.84 ± 0.01
Pept-12	Gln–Asp	0.18 ± 0.02	0.23 ± 0.03	Pept-72	Ser–Thr	0.51 ± 0.03	0.51 ± 0.02
Pept-13	Leu–Leu	0.06 ± 0.02	0.12 ± 0.01	Pept-73	Met–Pro	0.64 ± 0.02	0.36 ± 0.03
Pept-14	Met–Glu	0.10 ± 0.01	0.02 ± 0.01	Pept-74	Glu–Ile	0.59 ± 0.04	1.03 ± 0.04
Pept-15	Pro–Asn	0.11 ± 0.01	0.10 ± 0.01	Pept-75	Ser–Leu	0.63 ± 0.06	0.64 ± 0.02
Pept-16	Thr–Trp	0.13 ± 0.00	0.48 ± 0.02	Pept-76	Thr–Glu	0.55 ± 0.04	0.32 ± 0.03
Pept-17	Val–Trp	0.10 ± 0.01	0.27 ± 0.04	Pept-77	Ile–Ser	0.53 ± 0.06	1.08 ± 0.03
Pept-18	Thr–Gly	0.25 ± 0.03	0.28 ± 0.04	Pept-78	Thr–Ala	0.55 ± 0.05	0.46 ± 0.04
Pept-19	Glu–Pro	0.17 ± 0.01	0.12 ± 0.01	Pept-79	Ile–Gln	0.61 ± 0.05	0.32 ± 0.04
Pept-20	Glu–Tyr	0.19 ± 0.01	0.25 ± 0.01	Pept-80	Leu–Arg	0.75 ± 0.11	0.63 ± 0.09
Pept-21	His–Asp	0.23 ± 0.02	0.22 ± 0.01	Pept-81	Val–Glu	0.60 ± 0.05	0.72 ± 0.02
Pept-22	Val–Met	0.15 ± 0.01	0.41 ± 0.04	Pept-82	Val–Phe	0.66 ± 0.05	0.88 ± 0.12
Pept-23	Pro–Asp	0.19 ± 0.01	0.22 ± 0.01	Pept-83	Val–Asn	0.55 ± 0.06	0.86 ± 0.04
Pept-24	Glu–Gln	0.15 ± 0.02	0.13 ± 0.01	Pept-84	Glu–Gly	0.75 ± 0.03	0.79 ± 0.04
Pept-25	His–Gly	0.45 ± 0.05	0.41 ± 0.04	Pept-85	Ile–Thr	0.76 ± 0.05	0.63 ± 0.02
Pept-26	Gln–Gly	0.33 ± 0.02	0.17 ± 0.01	Pept-86	Gly–Arg	0.88 ± 0.04	0.77 ± 0.02
Pept-27	Thr–Ser	0.21 ± 0.01	0.14 ± 0.01	Pept-87	Thr–Leu	0.80 ± 0.04	1.02 ± 0.07
Pept-28	Val–Asp	0.21 ± 0.01	0.30 ± 0.02	Pept-88	Val–Arg	0.86 ± 0.09	1.58 ± 0.10
Pept-29	Gln–Asn	0.24 ± 0.01	0.17 ± 0.01	Pept-89	Gln–Pro	0.83 ± 0.04	0.09 ± 0.05
Pept-30	Ala–Thr	0.18 ± 0.02	0.08 ± 0.00	Pept-90	Val–Val	1.09 ± 0.04	3.70 ± 0.16
Pept-31	Gln–Tyr	0.24 ± 0.01	0.04 ± 0.01	Pept-91	His–Pro	1.13 ± 0.03	0.13 ± 0.01
Pept-32	Glu–Trp	0.29 ± 0.04	0.64 ± 0.04	Pept-92	Val–Thr	0.76 ± 0.08	0.83 ± 0.07
Pept-33	Pro–Gly	0.28 ± 0.01	0.36 ± 0.01	Pept-93	Thr–Arg	0.80 ± 0.14	0.91 ± 0.07
Pept-34	Glu–His	0.31 ± 0.01	0.49 ± 0.02	Pept-94	Arg–Glu	1.06 ± 0.04	0.69 ± 0.07
Pept-35	Gln–Met	0.19 ± 0.03	0.05 ± 0.01	Pept-95	Gln–Ser	0.87 ± 0.08	0.52 ± 0.05
Pept-36	Pro–Ala	0.45 ± 0.08	0.63 ± 0.06	Pept-96	Ser–Pro	0.97 ± 0.05	0.11 ± 0.02
Pept-37	Asn–Phe	0.31 ± 0.01	0.06 ± 0.01	Pept-97	Phe–Pro	1.55 ± 0.08	0.82 ± 0.03
Pept-38	Pro–Gln	0.29 ± 0.01	0.40 ± 0.01	Pept-98	Gln–Leu	1.03 ± 0.09	0.15 ± 0.03
Pept-39	Gln–Thr	0.20 ± 0.02	0.21 ± 0.04	Pept-99	Ile–Gly	1.42 ± 0.04	2.18 ± 0.09
Pept-40	Pro–Phe	0.59 ± 0.11	1.38 ± 0.06	Pept-100	Ile–Val	1.30 ± 0.02	5.35 ± 0.15
Pept-41	Ser–Ile	0.28 ± 0.01	0.35 ± 0.02	Pept-101	Val–Gln	1.05 ± 0.10	1.11 ± 0.09
Pept-42	Tyr–Glu	0.31 ± 0.02	0.02 ± 0.00	Pept-102	Ala–Pro	1.44 ± 0.02	0.15 ± 0.01
Pept-43	Glu–Thr	0.22 ± 0.03	0.24 ± 0.03	Pept-103	Val–Ile	1.46 ± 0.08	3.29 ± 0.11
Pept-44	Pro–Val	0.47 ± 0.06	2.35 ± 0.15	Pept-104	Ile–Glu	1.39 ± 0.07	1.61 ± 0.03
Pept-45	His–Asn	0.28 ± 0.03	0.33 ± 0.00	Pept-105	Lys–Pro	1.73 ± 0.03	0.25 ± 0.03
Pept-46	Pro–Thr	0.34 ± 0.02	0.53 ± 0.03	Pept-106	Ala–NorLeu	1.77 ± 0.07	1.31 ± 0.07
Pept-47	Phe–Gly	0.41 ± 0.03	0.39 ± 0.03	Pept-107	Val–Ala	1.06 ± 0.17	1.91 ± 0.12
Pept-48	Asp–Pro	0.33 ± 0.01	0.22 ± 0.02	Pept-108	Val–Lys	1.87 ± 0.05	3.26 ± 0.09
Pept-49	Ser–Lys	0.31 ± 0.02	0.24 ± 0.01	Pept-109	Ile–Lys	2.02 ± 0.09	2.43 ± 0.08
Pept-50	Gln–Ile	0.35 ± 0.02	0.30 ± 0.04	Pept-110	Arg–Pro	2.29 ± 0.02	0.12 ± 0.01
Pept-51	Asn–Lys	0.22 ± 0.05	0.97 ± 0.04	Pept-111	Ile–Ala	2.07 ± 0.12	2.47 ± 0.04
Pept-52	Ile–His	0.44 ± 0.03	0.82 ± 0.01	Pept-112	Thr–Pro	2.14 ± 0.06	0.46 ± 0.05
Pept-53	His–Ala	0.59 ± 0.11	0.67 ± 0.04	Pept-113	Ala–Lys	5.29 ± 0.88	3.16 ± 0.35
Pept-54	Pro–Glu	0.40 ± 0.03	0.45 ± 0.04	Pept-114	Pro–Pro	3.51 ± 0.30	4.72 ± 0.26
Pept-55	Leu–Gly	0.61 ± 0.06	0.60 ± 0.04	Pept-115	Val–Pro	4.24 ± 0.11	0.99 ± 0.07
Pept-56	His–Glu	0.44 ± 0.03	0.23 ± 0.03	Pept-116	Ile–Leu	3.52 ± 0.21	3.71 ± 0.19
Pept-57	Ala–Glu	0.40 ± 0.02	0.42 ± 0.04	Pept-117	Leu–Val	3.94 ± 0.33	3.26 ± 0.37
Pept-58	Gln–Ala	0.39 ± 0.02	0.22 ± 0.02	Pept-118	Ile–Pro	6.51 ± 0.15	5.58 ± 0.16
Pept-59	Val–His	0.44 ± 0.02	0.88 ± 0.04	Pept-119	Leu–Pro	7.97 ± 0.32	3.31 ± 0.17
Pept-60	Ser–Glu	0.34 ± 0.04	0.27 ± 0.03				

a Unique identified tripeptide.

**Figure 2:**
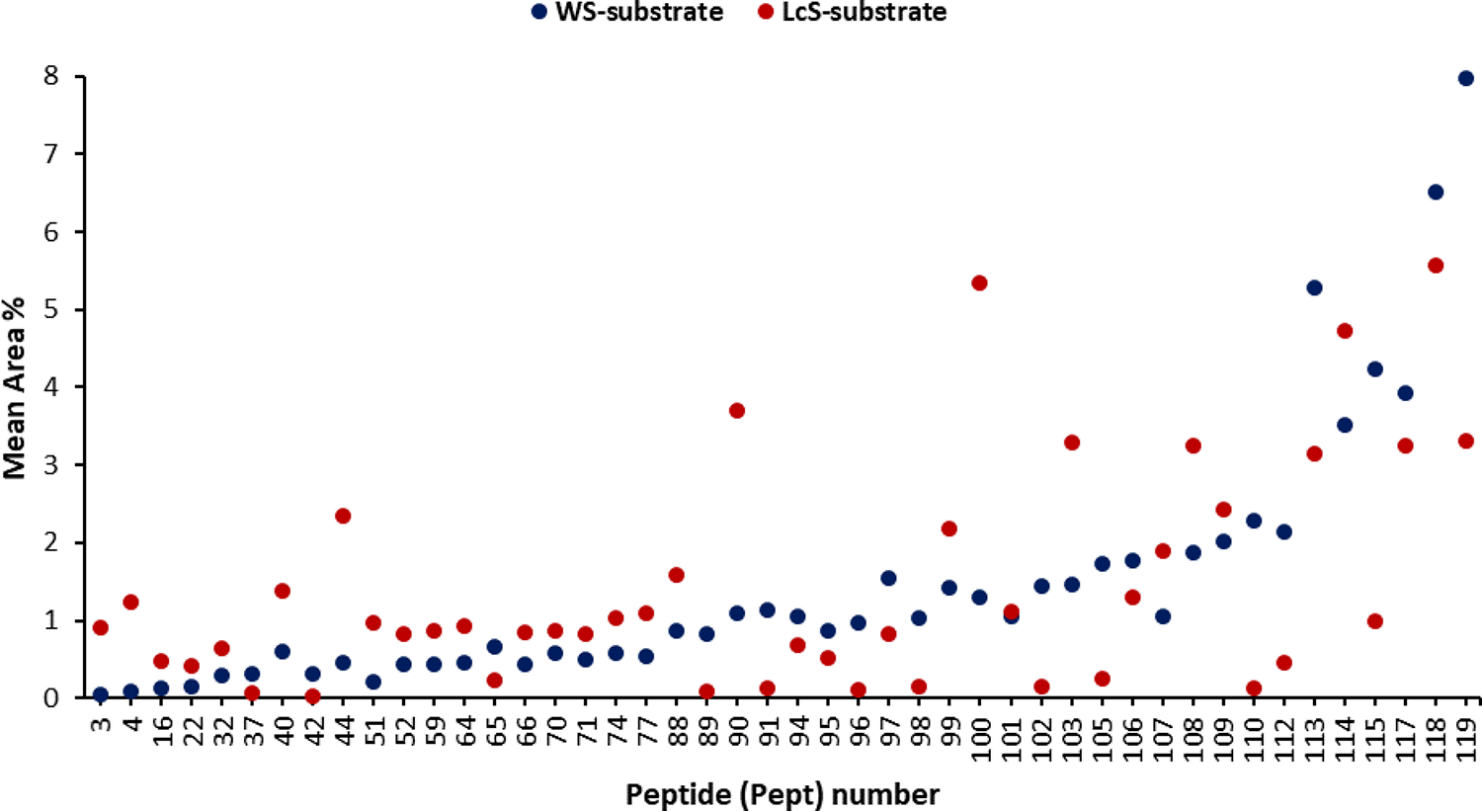
Normalized distribution (area values) of the most differentially expressed dipeptides based on the comparison between the two growth substrates (WS and LcS). Points represent *P. ostreatus* strains grown on the respective substrate and are reported as mean values (*n* = 6). The corresponding number for each dipeptide is defined in Table 2.

An analogous data analysis could be made by considering the dipeptide fraction of metabolites. As evident from Figure 2 and Table 2, a different expression of such compounds was observed for *P. ostreatus* samples grown in different substrates. Considering the relative abundance on the basis of the area values, the WS substrate composition was found to particularly affect the expression of Pro-containing dipeptides such as His–Pro (91), Ser–Pro (96), Phe–Pro (97), Ala–Pro (102), Lys–Pro (105), Arg–Pro (110), Thr–Pro (112), Val–Pro (115), Ile–Pro (118), and Leu–Pro (119) (Figure 2 and Table 2). On the contrary, a marked influence by the LcS substrate was found in the expression of peptides such as Pro–Phe (40), Pro–Val (44), Val–Val (90), Ile–Val (100), Val–Ile (103), and Val–Lys (108) (Figure 2 and Table 2).

Multivariate statistical evaluations, performed to analyze the LC/MS Q-TOF data and elucidate the discrimination in the metabolic profile among the investigated samples, are described in the next section.

### Multivariate Statistical Analysis

**Figure 3:**
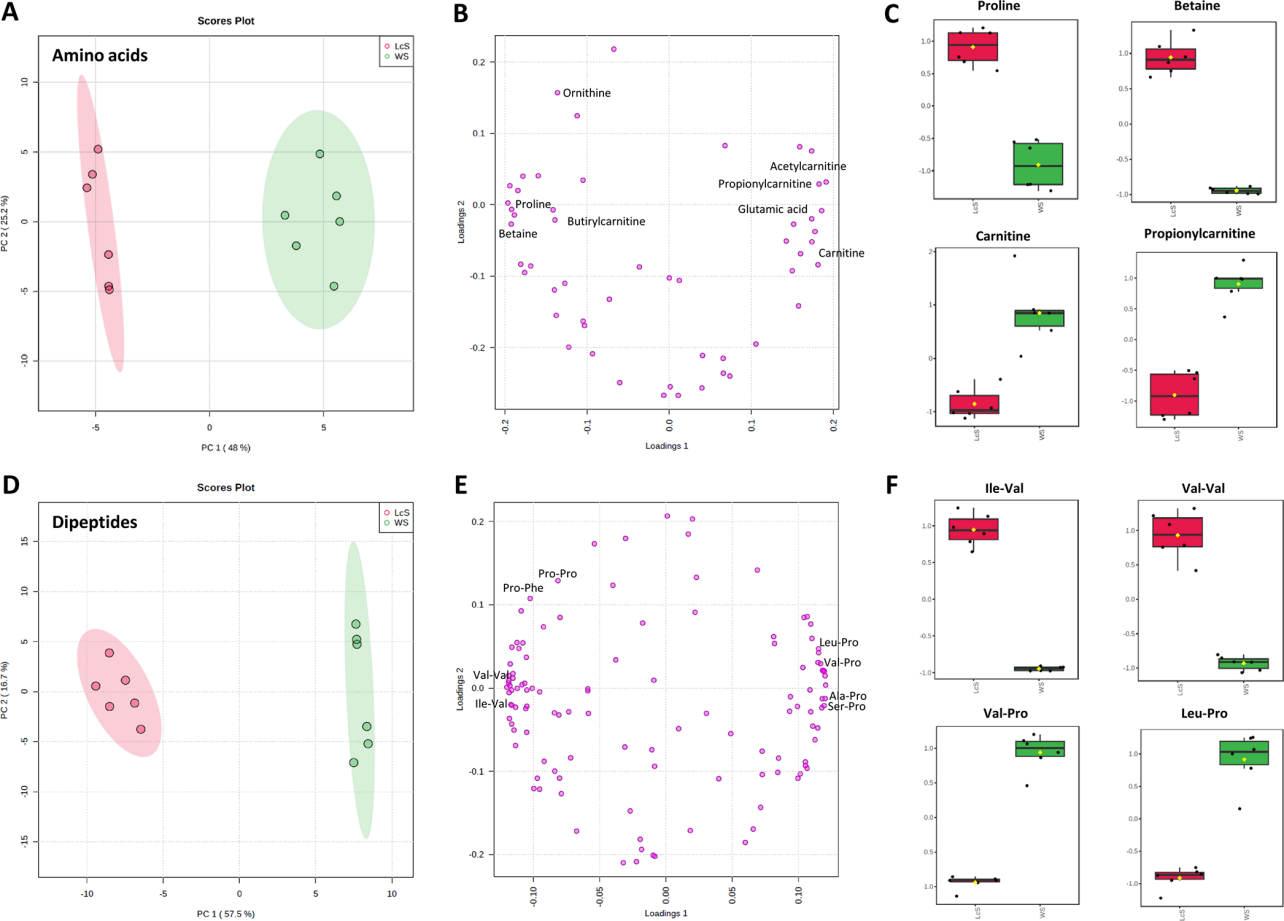
Principal component analysis (PCA) (A) score plot and (B) loading plot visualization using 53 identified amino acids; (C) boxplot of representative, significantly different, amino acids grown on the WS or LcS substrate; PCA (D) score plot and (E) loading plot visualization using 119 identified dipeptides; and (F) boxplot of representative, significantly different, dipeptides grown on the WS or LcS substrate.

The comprehensive multivariate statistical analysis was performed using the MetaboAnalyst web platform (5.0). To interpret the large data set generated, a principal component analysis (PCA) was selected as the useful method to reduce the dimensionality of the MS data while preserving as much the system variability to allow illustrating the discrimination in the amino acids and dipeptide profile among the *P. ostreatus* samples. Accordingly, the unsupervised PCA-based multivariate statistical approach was able to discriminate *P. ostreatus* extracts in relation to the growth substrate in two distinct clusters (Figure 3A,D).

Concerning the amino acids, the PCA score plot showed a notable distinction between the two *P. ostreatus* samples, as evidenced in the net shape of the plotted point swarm, relatively to the WS and LcS substrates (Figure 3A). This, in turn, reflects the metabolic differences generated by the two growth substrates. The first and second principal components (PC1 and PC2) explained 73.2% of the total variance. The loading plot clearly evidenced “peripheral” variables, not clustered with the central group. Such species are those displaying the most noticeable differences between the two substrates, thus configuring as the main contributors to the found discrimination (Figure 3B). More specifically, amino acids such as proline and betaine are mainly expressed in *P. ostreatus* samples grown on the LcS, while carnitine and propionylcarnitine are more representative in samples grown on the WS substrate. The exemplary boxplots, shown in Figure 3C, allow visualizing the above significant differences.

Analogously, the PCA model distinguished the investigated mushroom samples on the basis of the dipeptide profile. The PCA score plot, indeed, showed two well-distinct clusters of each sample in the score plot of PC1 and PC2 accounting for a 74.2% overall variance (Figure 3D). The applied statistical model, with PC1 contributing alone for the highest proportion of the total variance information (57.5%), indicated a significant metabolic diversity among the investigated samples. The obtained results agree with our previous observation that metabolic profiles could be differently affected by the substrate. The loading plot (Figure 3E) clearly shows two compact clusters on both sides of the graph, highlighting a relationship between the PCs and the original variables. Dipeptides such as Ile–Val, Val–Val, Leu–Pro, and Val–Pro are among the species contributing mainly to the observed discrimination. Accordingly, the exemplary boxplots, shown in Figure 3F, evidence a pronounced expression of Ile–Val and Val–Val in the samples grown on the LcS, while Leu–Pro and Val–Pro results are prevalent in the *P. ostreatus* samples grown on the WS.

**Figure 4:**
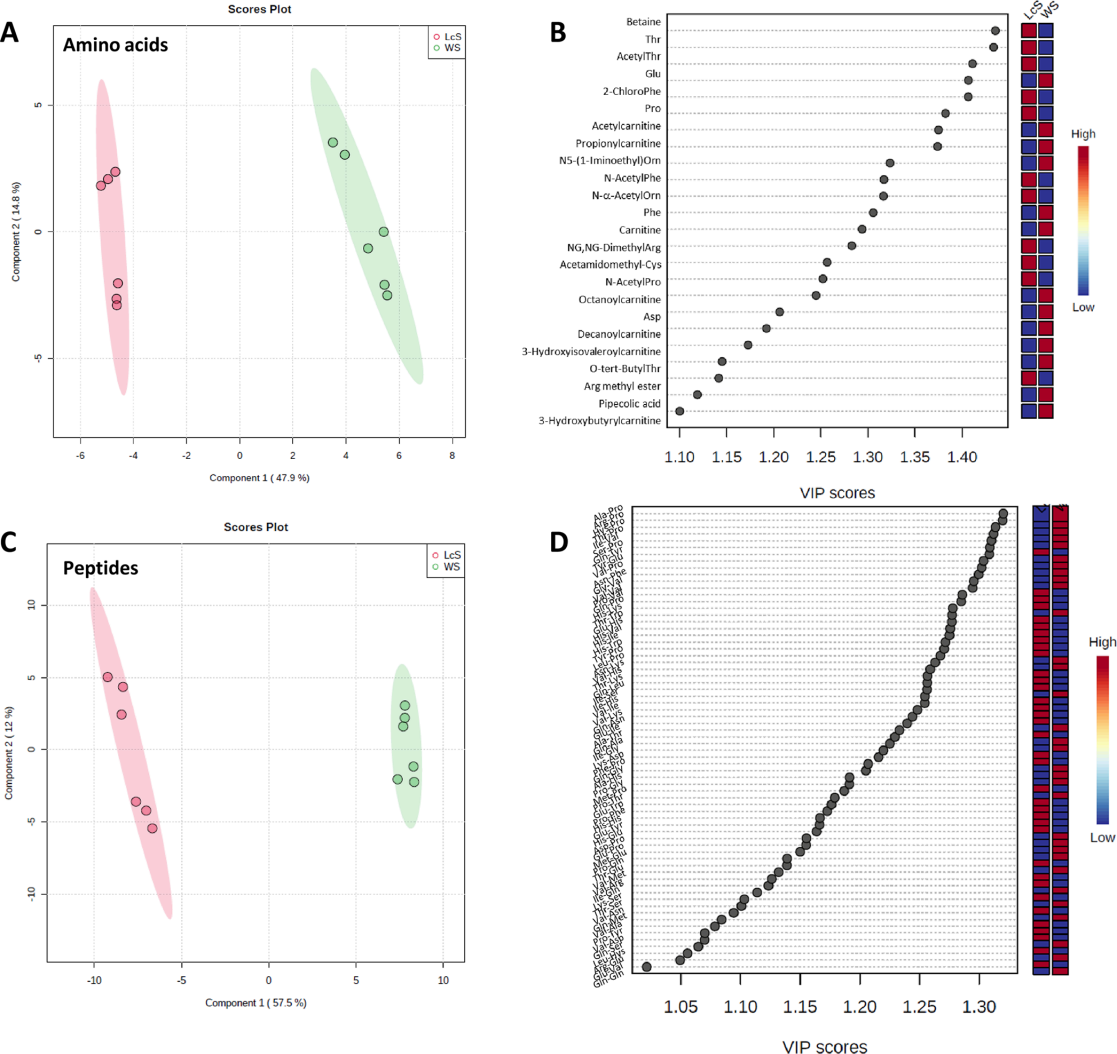
Partial least-squares discriminant analysis (PLS-DA) score plot from (A) amino acids and (C) dipeptide metabolite profiles. The variable importance in projection (VIP) score of (B) differential amino acids and (D) differential dipeptides in the multivariate data set (VIP scores > 1).

A further optimization of the separation was achieved through the supervised partial least-squares data analysis (PLS-DA)^34^ applied to identify and confirm features that could discriminate the metabolic variations between the two groups of *P. ostreatus* on the first component for both amino acids and dipeptides (Figure 4A,C). A clear visualization of metabolites in the extracts that significantly contributed to variability was obtained by means of the variable importance in projection (VIP) score plot. Actually, the information on VIP scores generated by the PLS-DA model is generally combined to gain a deeper knowledge of variables that mostly contribute to the underlying variation.^35^ Plots in Figure 4B,D summarize the contribution a variable makes to the model; the colored boxes on the right indicate the relative concentrations of the corresponding metabolite in the groups under evaluation. Both plots refer to selected metabolites characterized by a VIP score > 1, typically used as the threshold value for selecting relevant variables, and *p* < 0.01 (one-tailed Student’s *t*-test), identified as significant differential metabolites.

Specifically, 24 amino acids and 68 dipeptides emerged as discriminating metabolites between the two groups of *P. ostreatus*, respectively.

**Figure 5:**
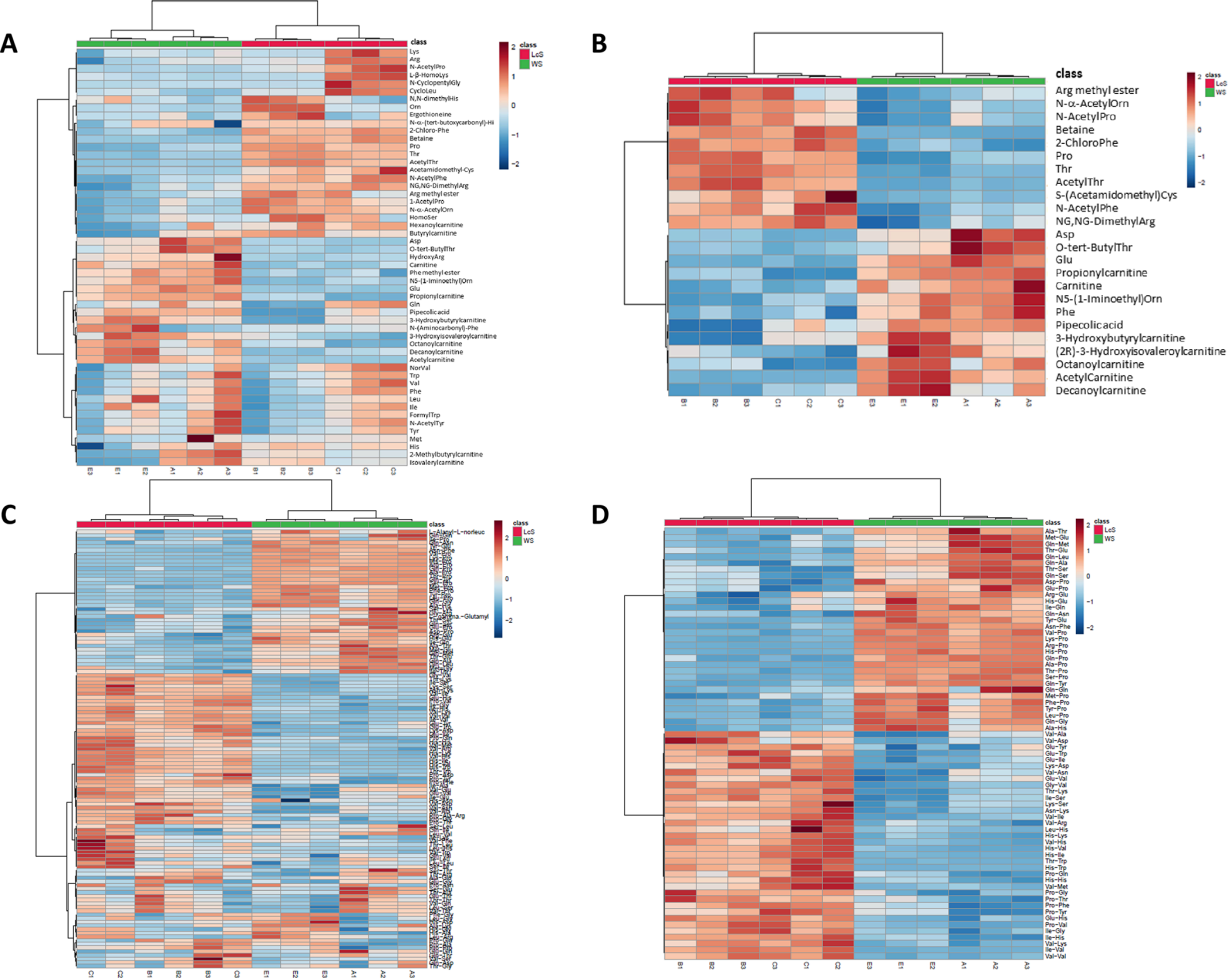
Heat map representation of the relative metabolite expression, affected by the *P. ostreatus* growth substrate, for (A) the whole pool of amino acids; (B) the most significant amino acids (24); (C) the whole pool of dipeptides; and (D) the most significant dipeptides (68). Metabolites are represented along the rows, and samples are represented along the columns. Metabolites significantly increased were displayed in red color, while metabolites significantly decreased were displayed in blue color. The brightness of each color corresponded to the relative abundance of species in each sample.

The information provided by the heat maps in Figure 5A showed a more straightforward visualization corroborating the above observation. Concerning the whole class of amino acid metabolites, clear variations among samples belonging to the two substrates, namely, WS and LcS, were observed in the heat map representation, which allowed us to distinguish, at a first glance, two main regions. Metabolites significantly increased are marked in red: the upper part of Figure 5A displays metabolites especially abundant in samples grown on the LcS substrate. On the contrary, metabolites characterized by an overexpression in samples grown on the WS substrate primarily cover the lower part of the graph. However, it is worth noting small areas characterized by a spread distribution, revealing those “vulnerable” metabolites for which a sharp distinction cannot be noticed on the basis of the growth substrate.

With the exceptions of hexanoylcarnitine and butyrylcarnitine, most of the carnitine-based derivatives have been generally associated with *P. ostreatus* samples grown on WS substrates. Such an aspect is clearly emphasized in the heat map reported in Figure 5B, built up with the above 24 differential amino acids. A previous study by Seline^36^ evidenced that mushrooms are richer in carnitine than plants and that the total carnitine concentration (free carnitine and acylcarnitine esters) in oyster mushroom (530 mg/kg DW) equals approximately the carnitine contents in pig muscles. Several beneficial effects in the treatment of aging, chronic degenerative diseases, and infections have been associated with the supplementation of carnitine and its acyl-derivatives.^37^ Therefore, the selection of the best environmental factors and growth substrates could be exploited to produce high-value-added mushrooms or to design potential functional foods by triggering the upregulation of such species. Accordingly, in a recent study, Di Piazza and co-workers^3^ evaluated the metabolic profile variation in *P. ostreatus* samples grown on lavender-enriched substrates. In another study, Koutrotsios and co-workers^38^ demonstrated how the elemental fingerprints of mushroom cultivation substrates affected the composition of the final products. Therefore, in line with the cited results, in our study, a certain influence of the substrate composition on the metabolic profile of *P. ostreatus* samples could be plausibly hypothesized. Similarly, the heat maps referred to dipeptides exhibited a distinct pattern of metabolites between WS and LcS (Figure 5C). Specifically, the plot shows three main regions of metabolites. In the upper part, the group of metabolites significantly expressed in the WS substrate are reported (red color), while dipeptides mainly expressed in the LcS substrate dominate in the central part. The third lower block outlines a dispersed representation in which a clear distinction of the metabolic profile cannot be referred to the nature of the substrate alone. The heat map plot of the dipeptide profile for the top 68 emerged as differential features is shown in Figure 5D.

As indicated above, an emerging aspect concerns the peculiar presence of Pro-based dipeptides in mushrooms grown on WS substrates, while a major heterogeneousness was found in samples grown on the LcS-enriched substrate. Recent years have witnessed an increasing interest in research on proline as a key regulator of multiple biochemical and physiological processes. Pro-based peptides are generally recognized to play crucial roles in signal transduction pathways and exert various biological functions including antimicrobial, immunomodulatory, and antioxidant properties.^39^ Moreover, prolyl-containing sequence units have been found in several naturally occurring linear and cyclic peptides with immunosuppressive and toxic activities.^40^ Based on such regulatory roles in cellular biochemistry, Pro and Pro-rich peptides may prove to be potential dietary supplements for promoting health beneficial effects. Additionally, the presence of certain dipeptides is likely correlated to the metabolic protein stability and, within this frame, the occurrence of Pro seems to increase the resistance to the proteolytic action of the most common proteases.^41,42^ Therefore, such Pro-based dipeptides could represent a potential strategy to improve the metabolic stability of novel peptide (or proteins) drug candidates.

Tanaka and co-workers demonstrated the potential role of the Tyr–Pro dipeptide from soy in improving impaired cognitive deficits in model mice with Alzheimer’s disease.^43^ An *in vitro* experiment by Foltz highlighted a series of Pro-based dipeptides (Ile–Pro, Arg–Pro, Lys–Pro, and Gly–Pro) exhibiting a notable ACE inhibitory activity, with IC_50_ values below 100 μM and with a high intestinal stability.^44^ Furthermore, Guo and co-workers reported the spontaneous cyclization of Pro-containing linear dipeptides in aqueous solution to give the corresponding cyclic diketopiperazines (DKPs), working as peptide/protein precursors or chiral catalysts.^45^ Due to their chiral, rigid, and functionalized structure, such cyclic dipeptides, most of which notably contain the Pro residue, could bind a large variety of receptors with high affinity, giving a wide spectrum of biological properties.^46^ For example, cyclo(His–Pro) was described to exert multiple biological activities in the central nervous system, suggesting its possible application in the therapy of chronic, age-related, and neurological diseases.^47^ The dipeptide cyclo(Arg–Pro) was shown to possess an interesting chemotherapeutic potential through the inhibition of chitinases.^48^ Further Pro-based DKPs, including cyclo(Ser–Pro), cyclo(Tyr–Pro), and cyclo(Leu–Pro), endowed with antifungal and antiviral activities, were identified in *Lactobacillus plantarum* and *Leuconostoc mesenteroides* fermented Chinese cabbages.^49^

### Metabolic Pathway Analysis

Metabolic pathway enrichment analysis was performed to identify which networks in *P. ostreatus* samples were significantly impacted by the growth substrate. To understand the biological meaning of the observed metabolic changes, the 24 differential amino acids identified on the criteria of VIP values were used to “enrich” the pathways to which the metabolites belonged. The enrichment analysis interpretation was referred to as the KEGG database, one of the most widely used and complete pathway databases.

**Figure 6:**
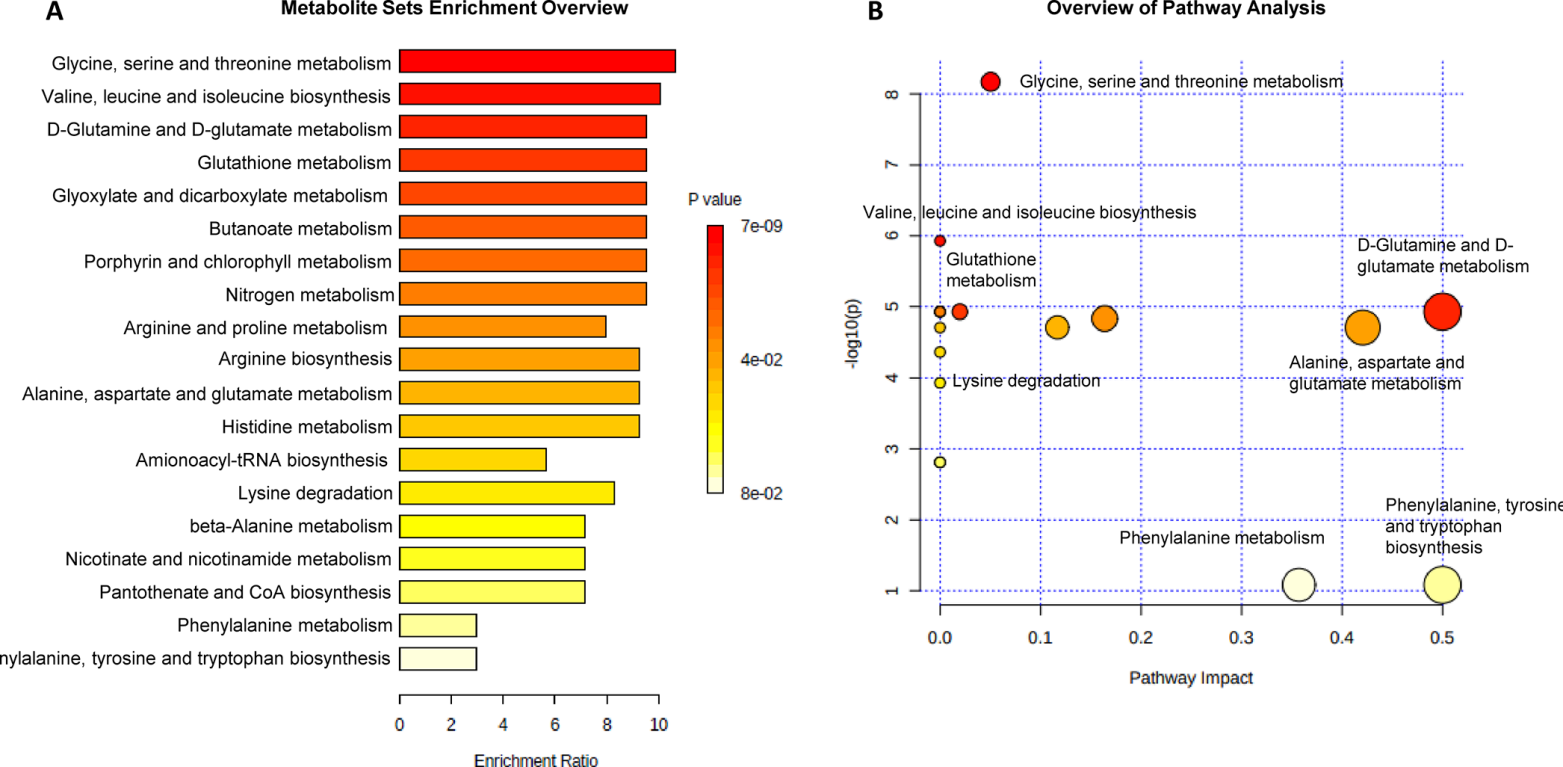
(A) MSEA showing the identified metabolic pathways based on the 24 differential amino acids: the horizontal bar graph summarizes metabolic pathways mainly affected by the growth of *P. ostreatus* samples on WS and LcS substrates; and (B) MetPA showing all matched pathways according to *p*-values from the pathway enrichment analysis (*y*-axis) and pathway impact values from the pathway topology analysis (*x*-axis). Small *p*-values and large pathway impact circles indicate that the pathway is greatly perturbed.

Metabolite set enrichment analysis (MSEA) was utilized to indicate which metabolic pathway may be mostly affected by the growth substrate. The color intensity in the MSEA overview (Figure 6A and Table 3S Supporting Information) reflects the statistical significance of the identified metabolic pathways: the most affected pathways related to the *P. ostreatus* growth on WS and LcS substrates are shown in red, while those altered to a lesser extent vary from orange to white. The obtained results identified 19 differentially expressed pathways, mainly concerning amino acid metabolism: the largest differential effect involved glycine, serine, and threonine metabolism, followed by valine, leucine, and isoleucine biosynthesis and d-glutamine and d-glutamate metabolism. Noteworthy, the first two pathways were also reported by Yan and co-workers^50^ among the metabolic processes, in *P. ostreatus* mycelia, associated with significant changes under heat-stress conditions.

Besides, other biosynthetic processes were identified that could complement the main *P. ostreatus* metabolism and correlated pathways. In the overview of metabolic pathway analysis (MetPA) (Figure 6B and Table 3S Supporting Information), the matched pathways are displayed as circles whose color and size are determined on the basis of the *p*-value from enrichment analysis and the pathway impact value from topology analysis, respectively. The results of this analysis showed target pathways that could be most significantly altered.

Interestingly, several metabolic pathways identified in our study have been reported in the literature mostly associated with the metabolism of yeasts and Ascomycetes mushroom family.^51−55^

However, their expression in Basidiomycetes cannot be excluded from being crucial pathways involved in regulatory mechanisms spanning from macromolecular metabolic processes to the potential detoxification role against pathogens and intracellular regulation mechanism against stressful conditions.^30,56−58^

Concerning the dipeptide fraction, the metabolic pathway enrichment analysis was not achievable. This may be plausibly ascribed to the putative presence of such species as intermediate products of protein biosynthesis or catabolism. However, further in-depth biochemical investigations are needed to explore the occurrence of such species with a particular glance at potentially interesting Pro-rich dipeptides.

In conclusion, the results obtained in our study highlighted the metabolic changes occurring in the investigated *P. ostreatus* samples, generated by the peculiar nature of the substrate. In particular, the choice of two growth substrates, namely, WS and LcS, was found to be effective in differentiating the expression of a large number of metabolites. LC/MS Q-TOF analyses, carried out to identify the metabolic profile, highlighted the well-known occurrence of amino acids, including both natural and non-natural species, and the noteworthy presence of a number of dipeptides, which represents an outstanding novelty of our work. More specifically, the use of the WS substrate revealed particularly discriminant in the expression of carnitine-based amino acid derivatives and Pro-based dipeptides.

As evidenced by our results, the specific substrate composition could be fruitfully exploited to emphasize the expression of well-known or potentially bioactive species producing enriched mushrooms or deriving useful functional ingredients from them. Such preliminary results encourage further experiments to be extended to a wider mushroom sampling as well as to a variation in the substrate nature to investigate more firmly the impact of different growth substrates on the phenotype.

## Supporting Information Available

Raw data files of the identified metabolites by LC/MS Q-TOF analysis (Tables 1S and 2S), the exemplary figure of fragmentation pattern (Figure 1S), and statistical data for the enrichment pathway analysis (Table 3S) (PDF)
