# Supplementary material for: LC/MS Q-TOF Metabolomic Investigation of Amino Acids and Dipeptides in Pleurotus ostreatus Grown on Different Substrates
Source: J Agric Food Chem. 2022 Aug 9;70(33):10371–82. doi: 10.1021/acs.jafc.2c04197 (PMC9413224; doi:10.1021/acs.jafc.2c04197)
Supplement: Supplementary file 1 — jf2c04197_si_001.pdf [file jf2c04197_si_001.pdf]

LC/MS Q-TOF metabolomic investigation of amino acids and dipeptides in *Pleurotus ostreatus* grown on different substrates

Roberto Maria Pellegrino,[a,†] Francesca Blasi,[b,†] Paola Angelini,[a] Federica Ianni,[b,*], Husam B.R. Alabed,[a] Carla Emiliani,[a] Roberto Venanzoni,[a] and Lina Cossignani,[b,c]

[a] Department of Chemistry, Biology and Biotechnology, University of Perugia, 06122 Perugia, Italy

[b] Department of Pharmaceutical Sciences, University of Perugia, 06126 Perugia, Italy

[c] Center for Perinatal and Reproductive Medicine, Santa Maria della Misericordia University Hospital, University of Perugia, Sant'Andrea delle Fratte, 06132 Perugia, Italy

[†] These authors have equally contributed to this work.

***Corresponding Author:**

Ianni Federica

Tel.: +075 5857955

E-mail address: federica.ianni@unipg.it

Table 1S. Overview of the identified metabolites by LC/MS Q-TOF analysis, annotated with NIST 2020 tandem mass library.

Rt (min)	m/z	Metabolite name	Adduct type	Reference m/z	Delta ppm	Formula	INCHIKEY	Total score	MS1 isotopic spectrum	MS/MS spectrum
8.805	154.08569	Vildagliptin	[M+H-C10H14O]+	154.0975	76.64	C17H25N3O2	SYOKIDBDQMKNDQ-XWTIBIIYSA-N	63.6	154.09229:14038 155.09564:942 156.099:0	97.08337:160 97.11013:50 154.14542:73
9.054	217.15691	Val-Val	[M+H]+	217.15469	10.22	C10H20N2O3	KRNYOVHEKOBTEF-UHFFFAOYSA-N	66.2	217.15691:326010 218.16026:43528 219.16362:5098	42.03619:98 55.05472:4280 55.11691:151 56.05523:234 56.09322:56 57.05893:216 72.08189:7784 72.15614:250 72.185:157 72.37117:54 73.0854:408
13.761	304.15823	Val-Trp	[M+H]+	304.16559	24.20	C16H21N3O3	LZDNBBYBDGBADK-KGLIPLIRSA-N	60.8	304.16354:33472 305.16689:8121 306.17025:1211	44.04936:55 55.05224:240 56.04889:134 57.05977:78 60.085:63 72.08155:1437 72.138:96 72.19753:56 130.06502:144 144.07237:121 145.08199:105 146.0578:478 188.06801:552 189.07874:173 198.88312:77
4.121	219.13571	Val-Thr	[M+H]+	219.1339	8.26	C9H18N2O4	GVRKWABULJAONN-QYNIQEEDSA-N	68.3	219.13304:62807 220.13639:7929 221.13975:920	47.0707:95 53.04128:124 55.05686:885 56.05951:112 57.05746:131 72.08139:2303 72.15357:114 73.08999:144 74.06578:70 84.05569:54 118.91647:67
3.417	205.12074	Val-Ser	[M+H]+	205.1183	11.90	C8H16N2O4	STTYIMSDIYISRG-WDSKDSINSA-N	66.7	205.12074:20394 206.12409:2648 207.12745:0	55.05237:694 56.05848:74 60.04446:53 72.07834:464

8.524	215.14049	Val-Pro	[M+H]+	215.13901	6.88	C10H18N2O3	GIAZPLMMQOERPN-YUMQZZPRSA-N	69.3	215.14049:351861 216.14384:42653 217.1472:4117	44.04496:124 55.05545:2988 55.12062:96 56.05722:226 56.08465:67 57.05662:122 70.06538:5686 70.1409:202 70.16547:83 71.06705:476 72.08176:4790 72.15569:208 72.18397:92 72.36788:50 73.07926:86 91.05276:91 116.07014:1286 116.4015:59 117.07:69
13.369	265.15295	Val-Phe	[M+H]+	265.15469	6.56	C14H20N2O3	GJNDXQBALKCYSZ-NEPJUHHUSA-N	62.6	265.15085:36094 266.1542:7523 267.15756:7680	55.05212:400 70.05971:81 72.07896:1547 108.04417:59 118.9118:62 120.07647:175 121.08079:103 136.91835:75
9.804	249.12721	Val-Met	[M+H]+	249.12669	2.09	C10H20N2O3S	YSGSDAIMSCVPHG-JGVFFNPUSA-N	63.5	249.12761:11819 250.13096:1782 251.13432:796	55.05395:185 56.04829:95 72.0797:343 96.07786:56
9.648	246.18425	Val-Lys	[M+H]+	246.1812	12.39	C11H23N3O3	JKHXYJKMNSSFFL-DTWKUNHWSA-N	68.3	246.18279:146309 247.18614:21216 248.1895:2316	55.05557:1514 56.05:121 57.05569:123 67.0548:51 69.0692:90 72.08141:3635 72.1544:165 73.08379:115 74.08187:56 84.08086:5580 84.15781:268 84.21529:71 85.08267:617 85.12876:77 85.26779:53 87.05257:128 112.07801:52 113.104:75 117.06268:60 121.08742:93 129.10617:107 130.08661:828 130.14726:75 131.09532:99 147.1113:73

12.186	231.17436	Val-Ile	[M+H]+	231.1703	17.56	C11H22N2O3	PNVLWFYAPWAQMU-DJLDEBSA-N	66.5	231.17392:131718 232.17727:20998 233.18063:2329	41.04037:76 55.05578:2082 56.05415:254 57.05513:139 69.07134:78 72.08244:4137 72.15668:135 72.26163:64 72.53194:68 73.08437:527 86.09599:387 118.92731:53 136.92125:55
10.073	255.14528	Val-His	[M+H]+	255.1452	0.31	C11H18N4O3	BNQVUHQWZGTIBX-RKDXNWHRSA-N	66.9	255.14914:29542 256.15249:3525 257.15585:775	41.0406:52 55.01808:70 55.05617:224 72.07988:367 83.06108:184 85.02464:57 93.04691:87 95.0605:105 105.07163:74 110.07085:1594 110.1609:72 111.06829:62 156.07626:86
5.3	247.13176	Val-Glu	[M+H]+	247.1288	11.98	C10H18N2O5	UPJONISHZRADBH-POYBYMJQSA-N	67.2	247.13129:52441 248.13464:5815 249.138:1115	41.03763:159 55.05574:851 55.09005:105 56.05739:85 57.06269:96 72.08173:1198 72.15641:57 73.08894:86 84.04134:399 85.04811:108 88.0778:52 102.06631:109 117.077:59
4.147	246.14642	Val-Gln	[M+H]+	246.14481	6.54	C10H19N3O4	XXDVDTMEVBYRPK-POYBYMJQSA-N	68.5	246.14642:129064 247.14977:17864 248.15313:2068	41.03913:118 55.05502:1278 55.12315:52 56.0538:183 57.05649:94 72.08181:2715 72.14297:151 72.30332:54 73.08337:88 84.04571:2539 84.12489:106 84.18593:53 86.0498:191 101.06682:197 102.05256:61

										130.05048:1092 158.92152:63
3.886	233.11707	Val-Asp	[M+H]+	233.1132	16.60	C9H16N2O5	OBTCMSPFOITUIJ-CAHLUQPWSA-N	66.5	233.11591:22234 234.11926:2503 235.12262:0	44.04722:109 55.05528:411 55.08739:56 56.0477:56 57.05824:98 72.08285:653 73.08289:57
2.977	232.13008	Val-Asn	[M+H]+	232.1292	3.79	C9H17N3O4	WITCOKQIPFWQQD-CAHLUQPWSA-N	65.8	232.13008:50106 233.13343:9860 234.13679:1564	44.05079:111 55.05584:612 57.0575:67 60.04285:86 68.04704:149 70.06025:52 72.08072:1666 72.13362:87 72.15714:70 74.02426:439 87.05406:120 116.02893:145 136.92912:55
11.191	274.19107	Val-Arg	[M+H]+	274.18741	13.35	C11H23N5O3	IBIDRSSEHFLGSD-JGVFFNPUSA-N	65.2	274.18762:88616 275.19097:13222 276.19433:1625	42.97935:52 55.0568:350 60.05543:319 70.06611:2041 70.14102:96 72.08114:2020 72.15565:78 73.08408:184 85.0661:73 86.09454:54 87.0819:69 99.09091:84 110.08666:66 112.09447:124 113.07706:79 113.14589:71 114.08326:77 115.08894:96 116.07461:384 118.91936:167

										130.09906:128 136.93228:168 140.07697:63 157.10464:90 158.08806:205 175.11642:243 176.13057:101 239.14386:64 261.10858:78
5.445	189.12323	Val-Ala	[M+H]+	189.1234	0.90	C8H16N2O3	HSRXSKHRSXRCFC-WDSKDSINSA-N	70.1	189.1255:146031 190.12885:16965 191.13221:1784	53.03786:125 55.05521:3528 55.1198:100 55.17471:72 56.05784:410 57.05569:361 58.06755:66 72.08119:3584 72.15427:84 72.18786:70 72.2274:70 73.08307:209 84.08081:67
1.122	61.04026	Urea	[M+H]+	61.0396	10.81	CH4N2O	XSQUKJJJFZCRTK-UHFFFAOYSA-N	66	61.03996:2933 62.04331:0 63.04667:0	43.00614:72 43.02899:218 44.01361:5326 44.09539:115 45.01363:60
10.413	279.13483	Tyr-Pro	[M+H]+	279.13391	3.30	C14H18N2O4	VNYDHJARLHNEGA-RYUDHWBXSA-N	68.7	279.13617:52539 280.13952:9643 281.14288:1658	70.0665:1432 70.14153:61 71.06332:53 91.05454:885 92.05972:104 109.06656:82 114.0542:53 116.06789:359 118.06886:54 119.04931:286 121.0583:136 136.07396:1019 137.07796:100 142.95074:69 147.04169:109
7.074	311.12723	Tyr-Glu	[M+H]+	311.12381	10.99	C14H18N2O6	PDSLRCZINIMU-QWRGUYRKSA-N	60.7	311.12643:19162 312.12978:3794 313.13314:806	45.03059:60 91.05808:394 92.05885:77 104.10293:51 110.06616:53 119.05165:356 130.04631:76 136.07639:386

										137.07196:101 150.0415:65
12.533	161.10872	Tryptamine	[M+H]+	161.1073	8.81	C10H12N2	APJYDQYYACXCRM-UHFFFAOYSA-N	70.2	161.10654:97640 162.10989:13494 163.11325:843	51.02283:193 63.0179:56 65.0395:230 66.03239:116 77.04113:298 78.0489:112 89.03737:350 90.03807:56 91.05362:1418 91.14342:58 92.06156:175 93.05669:132 102.04993:98 103.05406:169 104.05883:108 115.05383:2010 115.14814:73 116.05782:545 117.06538:1801 118.07052:285 119.07544:77 127.0546:1298 127.15098:82 128.05225:411 129.05881:187 130.05742:59 132.08168:74 133.09245:92 142.06531:203 143.07245:1510 143.18126:68 144.07655:1055 145.0925:220
10.536	149.06097	Tropic acid	[M+H-H2O]+	149.05969	8.59	C9H10O3	JACRWUWPXAESPB-UHFFFAOYSA-N	66.4	149.05922:19811 150.06257:2554 151.06593:0	51.02534:120 77.04131:1096 77.11346:68 79.05754:261 80.05895:135 91.05556:52 92.05896:92 102.05476:74 103.05954:276 104.05242:69

10.535	131.05034	trans-Cinnamic acid	[M+H-H2O]+	131.0491	9.46	C9H8O2	WBYWAXJHAXSJNI-VOTSOKGWSA-N	66.4	131.05005:17597 132.0534:2012 133.05676:0	77.04008:418 103.05578:74
1.364	139.00394	trans-Aconitic acid	[M+H-2H2O]+	139.00259	9.71	C6H6O6	GTZCVFVGUGFEME-HNQUOIGGSA-N	64.1	139.00166:2288 140.00501:627 141.00837:0	68.99852:203 69.03954:82
13.16	306.14648	Thr-Trp	[M+H]+	306.14481	5.45	C15H19N3O4	KAFKKRJQHOECGW-WQHBLYJGSA-N	63.2	306.14221:30313 307.14556:6220 308.14892:977	56.04949:148 74.06071:279 75.05736:52 85.03267:85 118.0671:96 120.07237:138 136.93103:87 144.07628:125 146.05988:322 159.09396:89 170.0499:74 188.06387:459
2.157	221.11325	Thr-Thr	[M+H]+	221.1132	0.23	C8H16N2O5	DSGIVWSDDRDJIO-KAZBKCHUSA-N	62	221.11568:23012 222.11903:2456 223.12239:0	56.04871:290 58.06362:189 74.0594:474 84.04565:128
1.726	207.09567	Thr-Ser	[M+H]+	207.0975	8.84	C7H14N2O5	GXGHLJTHMDII-WISUUJSJSA-N	66.4	207.09967:17312 208.10302:3237 209.10638:0	56.05029:329 57.03868:102 60.04561:178 74.06038:380
6.106	217.11884	Thr-Pro	[M+H]+	217.1183	2.49	C9H16N2O4	QOLYAJSZHIJCTO-VQVTYTSYSA-N	69.6	217.121:176310 218.12435:19371 219.12771:4459	55.05254:93 56.0498:1264 56.11044:54 57.03467:397 58.06591:112 59.03355:85 69.07259:72 70.06616:4681 70.1393:167 70.21783:62 70.23931:70 71.07054:229 74.06083:2109 74.13419:76 75.05883:63 86.09563:108 116.07079:661 117.06812:68
6.549	248.15962	Thr-Lys	[M+H]+	248.16051	3.59	C10H21N3O4	YKRQRPFODDJQTC-GJMOJQLCSA-N	64.3	248.1613:45231 249.16465:6275 250.16801:679	56.04987:130 57.03093:94 74.05878:527 84.07977:1033 85.03062:114 85.08408:75

										86.10981:62 131.08189:76 147.11096:52
11.294	233.15691	Thr-Leu	[M+H]+	233.1496	31.35	C10H20N2O4	BQBCIBCLXBKYHW-BIIVOSGPSA-N	63.6	233.1507:50736 234.15405:6074 235.15741:1048	43.05305:94 56.04869:542 56.08965:58 57.03092:181 74.0598:1305 75.06843:85 86.09901:164 87.09486:56 100.11089:51 118.91803:241 136.92789:96
1.94	177.08929	Thr-Gly	[M+H]+	177.08701	12.88	C6H12N2O4	BIYXEUAFGLTAEM-WUJLRWPWSA-N	66.1	177.08681:14285 178.09016:4085 179.09352:530	54.03273:83 56.04922:540 57.03769:102 74.06538:210 84.0421:53
2.562	249.10776	Thr-Glu	[M+H]+	249.10809	1.32	C9H16N2O6	BECPPKYKPSRKCP-JCGDXUMPSA-N	61.4	249.11093:45419 250.11428:6703 251.11764:1280	56.05095:127 57.03691:74 60.08488:78 74.06184:179 84.04642:156 85.04212:67
8.18	276.15802	Thr-Arg	[M+H]+	276.1666	31.07	C10H21N5O4	HYLXOQURIOCKIH-DSYKOEDSSA-N	60.9	276.16641:84746 277.16976:13932 278.17312:1914	41.03763:92 43.01907:90 45.03344:127 56.05074:143 57.03532:129 59.03505:109 60.05548:312 69.72964:62 70.06722:1247 71.06765:318 72.08368:85 74.06078:633 86.09857:193 87.02829:74 88.07616:78 98.06377:165 99.02403:80 100.05281:188 112.08947:235 113.07433:75 114.11156:63 115.09147:84 116.07298:288 118.07064:81 126.06938:69

										130.05072:52<br>130.09499:77<br>136.92693:122<br>150.09772:132<br>170.10747:51<br>175.1198:274<br>178.09592:61<br>200.91641:69<br>213.13135:62<br>241.13681:60
2.564	191.10242	Thr-Ala	[M+H]+	191.1026	0.94	C7H14N2O4	VPZKQTYZIVOJDV-LMVFSUKVSA-N	65.8	191.10466:42828<br>192.10801:4755<br>193.11137:510	56.04897:195<br>57.03465:102 74.0583:270
9.422	265.11533	Thiamine cation	[Cat]+	265.11179	13.35	C12H17N4OS	JZRWCGZRTZMZEH-UHFFFAOYSA-N	60.9	265.11063:7219<br>266.11398:1607<br>267.11734:0	42.03396:81 81.04653:175<br>122.07275:203

15.331	242.28629	Tetrabutylammonium cation	[Cat]+	242.28419	8.67	C16H36N	DZLFLBLQUQXARW-UHFFFAOYSA-N	70.7	242.28629:1571399 243.28964:407341 244.293:34795	41.03912:4805 41.09568:181 41.11564:86 41.14586:72 42.0428:135 44.05042:1426 44.11067:86 45.05025:90 48.37786:75 55.06042:58 57.07045:7173 57.13741:255 57.17751:127 58.0659:14422 58.13163:671 58.15588:264 58.18802:160 58.25434:105 58.28602:91 58.34351:81 58.38648:62 58.44855:91 58.49096:74 58.51011:58 59.07082:268 67.05479:65 70.06944:58 72.08265:1167 73.08139:110 74.09635:2327 74.19257:65 75.09614:146 77.03043:53 79.04949:61 82.33197:60 84.07515:195 84.10123:57 86.09788:712 87.09892:69 100.11246:41565 100.1991:2268 100.4014:53 100.45583:136 100.50109:120 100.52877:88 100.58514:95 100.65312:61 100.72276:66 100.75869:72 101.01625:50 101.11611:3255 101.27108:79 101.34238:57

101.49202:73
101.61606:55
101.73919:54
102.08941:58
102.11535:164
102.13728:94
102.42632:52
114.12959:69
128.14235:2641
128.23721:93
128.27303:58
129.14571:200
30.15834:6047
130.25897:212
130.30945:134
131.16013:883
131.66562:57
141.49197:57
142.15907:42170
142.2603:2297
142.42366:206
142.46945:81
142.53831:87
142.66508:119
142.72699:86
142.84082:53
142.88422:58
142.92023:89
142.95343:58
143.04227:87
143.16068:4699
143.24734:280
143.31165:175
143.34178:149
143.38406:78
143.47917:80
143.65434:60
144.04536:51
144.16951:217
144.53508:53

										182.14653:56 184.20508:5417 184.32381:186 184.37401:101 184.42596:102 184.49474:76 185.20851:857 185.2843:62 186.22105:8418 186.33763:260 186.38362:162 186.42751:109 186.5197:93 186.6299:116 186.71111:66 186.76685:90 187.22357:1766 188.22319:137 242.28435:2407 242.42624:90 242.5002:53 242.6337:77
12.516	146.16388	Spermidine	[M+H]+	146.16521	9.10	C7H19N3	ATHGHQPFGPMSJY-UHFFFAOYSA-N	61	146.16353:776168 147.16688:89560 148.17024:4043	72.08087:714 84.08324:164 85.09202:62 119.07441:51
1.873	207.09627	Ser-Thr	[M+H]+	207.0975	5.94	C7H14N2O5	LDEBVRIURYMKQS-UOWFLXDJSA-N	67.3	207.09799:48383 208.10134:7586 209.1047:901	43.05338:53 56.049:304 57.0322:84 57.04881:54 60.04427:922 74.05899:240 120.06059:101 141.07161:53
4.204	203.10435	Ser-Pro	[M+H]+	203.1026	8.62	C8H14N2O4	WBAXJMCUFIXCNI-WDSKDSINSA-N	70	203.10435:104445 204.1077:12034 205.11106:1694	43.04036:63 60.04646:1443 60.11351:62 70.0662:2353 70.14169:88 71.06748:122 116.0677:326
5.578	234.14391	Ser-Lys	[M+H]+	234.14481	3.84	C9H19N3O4	SBMNPABNWKXNBJ-RQJHMYQMSA-N	66.8	234.14391:31717 235.14726:4388 236.15062:828	42.03696:104 56.04899:53 60.04605:355 84.0802:893 118.91942:78 129.10742:50 130.09103:58

10.725	219.1375	Ser-Leu	[M+H]+	219.1339	16.43	C9H18N2O4	NFDYGNFETJVMSE-RQJHMYQMSA-N	62.7	219.13516:55940 220.13851:7037 221.14187:861	42.03057:148 44.0464:95 57.03148:62 60.04319:1194 71.0677:76 86.09913:326 87.09286:87 102.94397:73 103.05275:95 118.91057:81
9.908	219.13617	Ser-Ile	[M+H]+	219.1339	10.36	C9H18N2O4	BXLYSRPHVMCOPS-RRKCRQDMSA-N	63.9	219.13118:25087 220.13453:3321 221.13789:615	41.03702:56 42.034:99 43.01832:67 45.05048:60 57.06129:104 60.04466:760 69.06982:162 86.09914:236 118.91747:123
2.146	235.09152	Ser-Glu	[M+H]+	235.0925	4.17	C8H14N2O6	LAFKUZYWNCHOHT-UHNVWZDZSA-N	64.6	235.09152:44107 236.09487:6461 237.09823:1096	42.03139:99 50.09751:69 60.0436:1067 60.11332:82 61.04029:65 70.06995:66 84.04359:819 84.1269:52 85.04289:127 85.44175:50 98.06739:58 102.05518:133 109.03665:53 130.0529:69
1.822	90.0554	Sarcosine	[M+H]+	90.055	4.44	C3H7NO2	FSYKKLYZXJSNPZ-UHFFFAOYSA-N	63.4	90.05695:4476 91.0603:0 92.06366:0	42.03469:101 43.01567:72 44.05026:10527 44.10876:389 44.12645:147 44.15922:116 44.3038:58 44.43998:66 44.66281:66 44.73191:57 44.99797:747 45.05246:300
2.066	134.02837	Acetamidomethyl cysteine	[M+H-C2H5NO]+	134.02699	10.30	C6H12N2O3S	QFQYGJMNIDGZSG-YFKPBYRVSA-N	60.9	134.02846:4275 135.03181:0 136.03517:0	44.05112:78 58.99686:207 60.98959:209 88.02439:222
12.508	72.08035	Pyrrolidine	[M+H]+	72.0808	6.24	C4H9N	RWRPKQPQOW-UHFFFAOYSA-N	63	72.08051:24395 73.08386:1640 74.08722:2733	42.03339:178 43.0404:68 44.04549:66 57.05611:62 72.07757:128 72.09748:62

7.796	215.14069	Pro-Val	[M+H]+	215.13901	7.81	C10H18N2O3	AWJGUZSYVIVZGP-YUMQZZPRSA-N	63.7	215.14069:203021 216.14404:26956 217.1474:3277	43.05545:53 70.06642:2881 70.13721:114 70.19227:57 71.06943:56 72.08075:76 78.03579:53 118.08894:55
9.455	279.13388	Pro-Tyr	[M+H]+	279.13391	0.11	C14H18N2O4	OIDKVWTWGDWMHY-RYUDHWBXSA-N	62.1	279.13589:3943 280.13924:679 281.1426:635	43.05826:55 70.06728:245
2.966	217.11885	Pro-Thr	[M+H]+	217.1183	2.53	C9H16N2O4	GVUVRRPYYDHHGK-VQVTYTSYSA-N	60.8	217.11931:20510 218.12266:2982 219.12602:914	44.05127:67 67.04964:141 68.04559:63 70.02683:101 70.06487:605
6.75	213.12869	Pro-Pro	[M+H]+	213.1234	24.82	C10H16N2O3	RWCOTTLHDJWHRS-YUMQZZPRSA-N	62.5	213.12407:244682 214.12742:28478 215.13078:5934	43.05542:193 70.06567:12990 70.13879:544 70.16665:189 70.26168:139 70.33511:121 70.43504:106 70.52003:54 71.07069:616 71.14332:53 99.05984:90 116.07009:198
12.706	263.14404	Pro-Phe	[M+H]+	263.13901	19.12	C14H18N2O3	IWIANZLCJVYEFX-RYUDHWBXSA-N	62.2	263.14066:107558 264.14401:20638 265.14737:2737	70.06519:3644 70.20641:66 71.06618:241 118.91826:84 120.07478:59
7.318	187.10823	Prolylalanine	[M+H]+	187.1077	2.83	C8H14N2O3	FELJDCNGZFDUNR-UHFFFAOYSA-N	62.1	187.10963:2985 188.11298:0 189.11634:7525	57.04705:96 70.06519:652
4.178	173.0941	Pro-Gly	[M+H]+	173.0921	11.55	C7H12N2O3	RNKSNIBMTUYWSH-YFKPBYRVSA-N	62.7	173.09126:9838 174.09461:1109 175.09797:0	41.04179:100 70.06521:536
3.497	245.11354	Pro-Glu	[M+H]+	245.1132	1.39	C10H16N2O5	QLROSWPKSBORFJ-BQBZGAKWSA-N	64.5	245.11256:26144 246.11591:3787 247.11927:794	70.06563:547

2.72	244.13066	Pro-Gln	[M+H]+	244.1292	5.98	C10H17N3O4	SHAQGFGGJSLLHE-BQBZGAKWSA-N	63.1	244.13231:26253 245.13566:4733 246.13902:673	56.04865:109 70.0667:2233 70.13775:95 70.18986:50 84.04395:342 84.08085:68
2.708	231.09877	Pro-Asp	[M+H]+	231.0975	5.50	C9H14N2O5	GLEOIKLQBZNKJZ-WDSKDSINSA-N	60.1	231.09892:13610 232.10227:1611 233.10563:0	70.0658:108
2.163	230.11342	Pro-Asn	[M+H]+	230.11349	0.30	C9H15N3O4	JQOHKCDMINQZRV-WDSKDSINSA-N	60.1	230.11372:10333 231.11707:2025 232.12043:0	70.06171:56
6.684	172.11455	Pro-Ala-Arg	[M+2H]2+	172.10809	37.53	C14H26N6O4	VXCHGLYSIOOZIS-UHFFFAOYSA-N	63.1	172.11455:3783 173.1179:586 174.12126:919	41.03749:204 42.03234:99 43.02866:1122 43.05441:1558 43.11605:51 43.34594:67 44.03719:132 46.06727:76 53.04128:149 53.0627:55 55.027:239 55.05369:231 56.04609:72 57.03405:86 57.07005:59 60.05615:4160 60.12466:184 60.15075:68 67.05567:403 68.04923:363 70.06612:39697 70.13917:2185 70.21603:74 70.29211:114 70.37391:100 70.42239:76 70.47812:106 70.56183:128 70.60428:55 70.96516:59 70.99144:68 71.0501:897 71.11448:75 72.08518:532 72.39101:60 73.06046:99 84.08415:632 85.06209:102 97.07232:207 98.06554:133 112.08411:53 116.06668:141

										130.10408:115 130.13831:73
3.493	187.10924	Pro-Ala	[M+H]+	187.1077	8.23	C8H14N2O3	FELJDCNGZFDUNR-WDSKDSINSA-N	65.7	187.10788:52060 188.11123:6207 189.11459:892	70.06628:1001 70.12289:55 71.04183:72 72.04478:82
18.57	167.03261	Phthalic acid	[M+H]+	167.03391	7.78	C8H6O4	XNGIFLGASWRNHJ-UHFFFAOYSA-N	66.7	167.0332:29223 168.03655:2858 169.03991:711	65.03739:901 65.07549:107 66.04279:120 93.03182:414 93.06886:50 121.02256:161
1.148	184.07262	Phosphocholine	[M+H]+	184.0733	3.69	C5H14NO4P	YHHSONZFOIEMCP-UHFFFAOYSA-N	68.6	184.07262:96448 185.07597:6961 186.07933:4252	44.05262:77 45.0337:848 45.0794:66 56.04885:139 58.06053:244 59.06796:119 59.09115:60 60.08175:288 71.0729:849 80.97437:391 86.09527:1214 86.179:75 87.0972:142 98.98487:1995

									99.04916:122 100.98462:86 104.09862:66 124.99689:487 125.09075:50 126.00359:95
13.032	263.13269	Phe-Pro	[M+H]+	263.13901	24.02	C14H18N2O3	WEQJQNWXCSUVMA-RYUDHWBXSA-N	70.4	263.14035:118113 264.1437:17468 265.14706:3166	70.06506:1765 70.13827:73 71.06998:90 72.04785:52 77.03465:277 91.04935:124 93.06586:362 103.05128:686 104.05209:82 116.06767:847 120.07911:4247 120.176:137 121.08433:256 132.07237:60 146.0441:54 147.05804:111 160.09061:58
11.895	223.11041	Phe-Gly	[M+H]+	223.1077	12.15	C11H14N2O3	GLUBLISJVJFHQS-VIFPVBQESA-N	63.8	223.11028:25229 224.11363:3602 225.11699:789	120.08092:762
7.615	147.04919	p-Coumaric acid	[M+H-H2O]+	147.0441	34.62	C9H8O3	NGSWKAQJJWESNS-ZZXKWVIFSA-N	68.9	147.04713:10547 148.05048:1233 149.05384:552	65.04132:189 91.05457:106
3.244	220.1196	Pantothenic acid	[M+H]+	220.1179	7.72	C9H17NO5	GHOKWGTUZJEAQD-UHFFFAOYSA-N	60.8	220.1169:64364 221.12025:7848 222.12361:1030	41.03819:84 43.01712:331 43.04611:54 55.01641:100 57.06841:237 59.0498:220 67.051:141 69.06993:96 70.02934:638 72.04301:207 73.02928:150 75.69403:53 77.0433:107 85.06319:81 88.02149:144 90.04818:171 94.06056:108 96.07964:135 98.01888:58

										99.00668:53<br>124.06865:155<br>124.10094:50
1.698	120.06734	O-tert-Butylthreonine	[M+H-<br>C4H8]+	120.0655	15.32	C8H17NO3	NMJINEMBBQVPG<br>Y-RITPCOANSA-N	66.9	120.06723:147829<br>121.07058:9039<br>122.07394:1830	41.03115:61 43.02437:130<br>43.04443:76 44.04948:65<br>46.03061:238<br>56.05048:2517<br>56.14328:53 57.04026:386<br>58.0304:122 58.06604:63<br>74.06319:114 75.05974:61<br>76.01595:69
15.918	288.21912	Octanoylcarnitine	[M+H]+	288.21689	7.74	C15H29NO4	CXTATJFJDMJMIY-<br>CYBMUJFWSA-N	62.3	288.2179:8870<br>289.22125:3474<br>290.22461:0	55.05307:57 57.07001:102<br>57.08704:59 59.07866:52<br>60.07966:67 69.072:66<br>85.02818:712<br>144.11455:56
3.328	124.04074	Nicotinic acid	[M+H]+	124.0393	11.61	C6H5NO2	PVNIIMVLHYAWGP<br>-UHFFFAOYSA-N	71.6	124.04074:150873<br>125.04409:13878<br>126.04745:1527	51.02422:146<br>52.01993:332<br>53.03951:1612<br>53.07409:164 53.10025:87<br>78.03476:2430<br>78.11172:66 79.04221:384<br>80.049:1505 81.05206:273<br>124.04147:265
11.767	116.07244	NG,NG-<br>Dimethylarginine	[M+H-<br>C3H9N3]+	116.0706	15.85	C8H18N4O2	YDGMGEXADBMO<br>MJ-LURJTMIESA-N	62.6	116.07103:24427<br>117.07438:1976<br>118.07774:571	70.06855:699<br>71.06545:146

12.808	188.07274	N-Formyltryptophan	[M+H-CH3NO]+	188.0706	11.38	C12H12N2O3	RNEMLJPSSOJRHX-UHFFFAOYSA-N	71	188.07234:449555 189.07569:61528 190.07905:5177	65.03569:53 90.04288:64 91.05295:2526 91.1384:87 91.35542:59 92.05348:188 103.0534:65 115.05213:1721 116.05704:539 117.05865:862 117.18151:52 118.06322:3780 118.15803:123 118.3343:65 119.0669:538 119.34892:54 127.04984:219 128.04755:171 140.04739:106 142.06152:868 143.0706:1282 144.07138:327 146.05986:132 170.06609:105
2.605	287.19733	N-Cyclopentylglycine	[2M+H]+	287.1965	2.89	C7H13NO2	LRHRHAWNXCGABU-UHFFFAOYSA-N	62.5	287.19836:14481 288.20171:2573 289.20507:0	58.064:77 71.07626:60 144.1028:1169 145.09712:96 146.11432:51
7.615	136.0811	N-Acetyltyrosine	[M+H-C3H4O3]+	136.0757	39.68	C11H13NO4	CAHKINHBCWCHCF-JTQLQIEISA-N	64	136.07713:77570 137.08048:6630 138.08384:0	41.03827:82 44.04217:52 51.02575:65 53.04073:91 63.02268:74 65.04076:780 66.04195:106 77.03796:203 78.04409:84 79.05994:90 82.07107:74 91.05722:1784 91.14362:57 92.05949:211 94.04434:148 106.06386:58 107.0496:380 107.09712:61 108.05651:89 118.91967:95

2.688	112.0774	1-Acetylproline	[M+H-CH2O2]+	112.0757	15.17	C7H11NO3	GNMSLDIYJOSUSW-LURJTMIESA-N	64.2	112.0774:4815 113.08075:0 114.08411:580	70.06256:109
9.346	208.09549	N-Acetyl phenylalanine	[M+H]+	208.0968	6.30	C11H13NO3	CBQJSKKFNMON-JTQLQIEISA-N	61.5	208.09549:3659 209.09884:1068 210.1022:0	77.04105:55 103.05415:110 107.03922:50 120.08083:326 133.04897:63
3.491	158.08226	N-Acetylproline	[M+H]+	158.08121	6.64	C7H11NO3	GNMSLDIYJOSUSW-ZCFIWIBFSA-N	60	158.08337:3565 159.08672:543 160.09008:683	43.01791:163 70.06426:313 70.09451:56
1.092	222.09695	N-Acetyl glucosamine	[M+H]+	222.0972	1.13	C8H15NO6	OVRNDRQMDRJTHS-PVFLNQBWSA-N	60.5	222.09898:7357 223.10233:1425 224.10569:1168	41.03871:57 43.01841:173 55.01418:73 60.04491:66 61.0304:169 72.04369:88 78.03716:123 81.03438:226 83.03979:108 84.04568:420 85.04842:102 96.0437:243 97.04606:101 98.04871:104 109.03131:57 126.05751:117 138.05902:249 138.11092:61
8.524	116.07121	N5-(1-Iminoethyl) ornithine	[M+H-C2H6N2]+	116.0706	5.26	C7H15N3O2	UYZFAUAYFLEHRC-LURJTMIESA-N	62.2	116.07088:21540 117.07423:1045 118.07759:730	41.03711:67 70.0657:1108 77.0349:111
12.516	112.11068	N1,N12-Diethylspermine	[M+H-C7H21N3]+	112.1121	12.67	C14H34N4	PGMIWVSHZMWSSI-UHFFFAOYSA-N	64.2	112.11054:16190 113.11389:1811 114.11725:0	41.03801:52 55.0535:83 55.07234:55 70.05893:70 84.08009:666 110.09898:122 112.1083:62

12.415	144.08228	N-[2-(1H-Indol-3-yl)ethyl] nicotinamide	[M+H-C6H6N2O]+	144.0808	10.27	C16H15N3O	ZDAZUJBASMCUAK-UHFFFAOYSA-N	68.6	144.08224:550277 145.08559:80740 146.08895:5019	41.03656:86 51.02573:74 52.02393:78 55.05512:67 58.06413:238 65.03798:276 67.03781:132 72.08061:1314 77.03714:818 78.04016:89 84.08142:226 89.03591:235 90.04459:288 91.05422:1558 91.17662:65 92.05279:252 101.03488:148 103.05327:200 104.04887:80 115.05338:2270 115.14757:91 116.05739:739 116.14196:50 117.06397:1740 117.15462:75 117.19855:52 117.29855:59 117.41367:55 118.06209:169 126.05034:136 127.05483:659 128.05533:172 129.05824:94 142.06354:568 142.12856:55 143.07167:1239 144.07513:390
2.692	175.11043	N-α-Acetylornithine	[M+H]+	175.1077	15.59	C7H14N2O3	JRLGPAXAGHMNOL-LURJTMIESA-N	63.4	175.11043:82696 176.11378:9244 177.11714:1068	43.01984:289 43.0564:114 70.06661:4909 70.13969:187 70.16072:99 70.28015:51 71.0702:692 81.03783:88 82.06788:95 140.93378:51

4.547	110.07248	N-α-(tert-Butoxycarbonyl) histidine	[M+H-C6H10O4]+	110.0713	10.72	C11H17N3O4	AYMLQYFMYHISQO-QMMMGPOBSA-N	65.7	110.07154:11380 111.07489:1325 112.07825:663	56.0509:228 66.03522:62 81.04433:183 82.05054:57 83.05494:60
3.319	184.10828	N,N-Dimethylhistidine	[M+H]+	184.10809	1.03	C8H13N3O2	IMOBSLOLPCWZKQ-ZETCQYMHSA-N	61.2	184.10747:3423 185.11082:943 186.11418:0	58.06728:66 68.04954:116 95.06015:216 138.03006:56 138.08974:80 138.10611:61
1.118	425.17398	N,N'-Diacetylchitobiose	[M+H]+	425.17661	6.19	C16H28N2O11	PLJAKLUDUPBLGD-VLWZLFBZSA-N	62.4	425.17398:1972 426.17733:895 427.18069:0	61.03353:75 84.04573:203 85.04018:59 126.05415:355 138.05673:224 144.05922:59 204.09656:63 244.07715:92
9.474	209.0965	N-(Aminocarbonyl) phenylalanine	[M+H]+	209.0921	21.04	C10H12N2O3	IPWQOZCSQLTKOI-QMMMGPOBSA-N	60.4	209.09564:4104 210.09899:628 211.10235:0	103.05635:83 118.07265:55 120.08073:263
13.936	212.11139	N-(4-Acetylphenyl)-4-methylbenzamide	[M+H-C2H2O]+	212.10699	20.74	C16H15NO2	WIFHKFKUUAUAKR-UHFFFAOYSA-N	64.6	212.11092:6797 213.11427:1511 214.11763:0	92.04789:67 119.05505:363 120.0558:64
1.911	117.10385	N-(3-Aminopropyl) acetamide	[M+H]+	117.1022	14.09	C5H12N2O	YFZBPSXRYCOKCW-UHFFFAOYSA-N	65.2	117.10232:9769 118.10567:1820 119.10903:0	56.05029:234 58.06466:471 59.07583:74
12.141	178.13322	N-(3-(Aminomethyl) benzylacetamidine	[M+H]+	178.1339	3.82	C10H15N3	RODUKNYOEVZQPR-UHFFFAOYSA-N	62	178.13322:2907 179.13657:682 180.13993:911	118.91999:62 120.08963:125 178.13306:140
9.995	247.11623	Met-Pro	[M+H]+	247.1111	20.76	C10H18N2O3S	DZMGFGQBRYWJOR-YUMQZZPRSA-N	68.9	247.11366:47966 248.11701:5792 249.12037:2854	56.05164:696 56.08734:69 61.01004:555 69.07378:61 70.02817:103 70.0662:786 104.05871:59 116.07211:430 118.07333:60 125.07734:57

5.832	207.08185	Met-Gly	[M+H]+	207.0798	9.90	C7H14N2O3S	QXOHLNCNYLGICT-YFKPBYRVSA-N	60	207.08157:4253 208.08492:0 209.08828:15118	56.05106:55 61.0095:309 84.03779:102
6.298	279.10059	Met-Glu	[M+H]+	279.10089	1.07	C10H18N2O5S	ADHNYKZHPOEULM-RQJHMYQMSA-N	64.1	279.10059:11957 280.10394:2459 281.1073:1376	56.0487:160 61.00899:183
1.07	685.23749	Melibiose	[2M+H]+	685.23969	3.21	C12H22O11	AYRXSINWFIIFAE-GFRRCQKTSA-N	66.3	685.23749:3006 686.24084:858 687.2442:0	85.02949:186 127.03507:97 145.05113:212 163.0576:103
1.077	667.22913	Maltotetraose	[M+H]+	667.22913	0.00	C24H42O21	LUEWUZLMQUOBSB-CHYJJPLWSA-N	61.8	667.23315:1481 668.2365:0 669.23986:0	97.03445:59 127.04062:201 145.048:202 163.05844:72
4.842	234.1456	Lys-Ser	[M+H]+	234.14481	3.37	C9H19N3O4	YSZNURNVYFUEHC-BQBZGAKWSA-N	64.1	234.14586:50300 235.14921:6452 236.15257:687	55.05573:117 56.04991:484 67.05531:80 84.08138:2735 84.16328:89 85.08257:182 102.93117:54 136.93462:89
10.035	244.16566	Lys-Pro	[M+H]+	244.1656	0.25	C11H21N3O3	AIXUQKMMBQJZCU-IUCAKERBSA-N	67.6	244.16792:132200 245.17127:16614 246.17463:6000	41.0408:52 43.01876:112 55.05289:61 56.05012:906 56.28376:65 57.05334:76 61.01166:565 67.05297:87 68.05881:61 70.06648:1587 70.14119:68 84.08198:7275 84.16164:324 84.19798:103 84.27502:52 85.0854:640 88.03954:60 94.0627:62 98.06058:112 116.07285:377 130.08061:76
5.986	262.14349	Lys-Asp	[M+H]+	262.1398	14.08	C10H19N3O5	CIOWSLJGLSUOME-BQBZGAKWSA-N	61.9	262.14191:50236 263.14526:7299 264.14862:1501	55.05699:155 56.05097:313 56.08002:64 59.06661:83 84.08177:2421 84.15921:121 84.18377:50

										85.08395:273 101.09949:106 164.93031:61
5.146	118.08896	Valine	[M+H]+	118.0863	22.53	C5H11NO2	KZSNJWFQEVHDMF-UHFFFAOYSA-N	70.8	118.08636:417403 119.08971:28045 120.09307:2330	40.02626:56 41.03822:507 42.03425:1312 42.11143:52 43.02014:179 43.40185:66 44.04871:150 45.00138:161 45.03416:429 53.03965:386 54.04059:227 55.05514:11969 55.11913:503 55.14323:194 55.17091:119 55.22021:75 55.26519:59 55.73561:54 56.05338:2269 57.05834:5526 57.12546:176 57.15339:89 57.49241:66 58.05985:116 59.05019:497 67.04383:66 72.08138:2870 72.1586:109 73.08208:96 102.92632:51
7.615	182.08827	Tyrosine	[M+H]+	182.08121	38.77	C9H11NO3	OUYCCCASQSFEME-UHFFFAOYSA-N	71.1	182.0834:464426 183.08675:51966 184.09011:5183	43.01936:140 43.03907:188 51.02347:316 65.03938:1335 65.5985:51 66.04633:92 67.05523:93 74.01435:113 77.04041:3784 77.11509:143 77.14334:60 77.81937:60 78.04333:243 79.05748:204 80.79215:50 81.06722:105 82.07141:107 89.04292:69 91.05495:16713 91.13911:727 91.17346:262

91.23411:154 91.38986:79
91.4453:70 91.478:62
91.50313:58 91.52796:92
91.91566:58
92.05739:2336
92.1416:109 92.23436:54
93.05215:121 93.26933:71
94.03535:131 94.0647:148
95.04957:4373
95.13487:173 95.16337:98
95.20634:76 95.38438:67
96.05146:381
103.05514:141
106.8864:50
107.04913:1375
108.05278:133
109.06447:254
117.06067:122
118.06442:414
119.04819:1910
119.14602:82
120.05541:136
121.06281:72
123.04356:1455
124.04494:98
136.07269:459
138.0733:51

12.808	205.09918	Tryptophan	[M+H]+	205.0972	9.65	C11H12N2O2	QIVBCDIJIAJPQS-SECBINFHSA-N	72	205.09856:724987 206.10191:109052 207.10527:10146	44.99562:54 46.0312:69 53.00028:84 54.03224:77 65.0365:97 66.03544:70 74.02299:348 77.04053:281 89.03532:132 90.04488:311 91.05424:3760 91.29662:55 92.05817:610 96.49704:63 101.03951:60 103.05547:287 103.10382:59 105.0733:175 115.05422:6393 115.1478:262 115.18456:100 115.25337:51 115.37315:90 116.05985:1149 116.14468:57 117.06097:4514 117.23311:84 117.28931:58 118.02477:56 118.06548:23109 118.15896:1220 118.19476:384 118.28411:218 118.33676:151 118.42065:162 118.47708:166 118.58171:89 118.62014:82 118.65614:86 118.67455:55 118.85494:68 118.90833:67 118.96304:74 119.06834:3033 119.19292:109

119.24722:57
119.38553:54
120.07427:129
127.05448:1289
127.16088:68
127.34328:54
128.05685:318
129.05101:59
130.06593:2654
130.15645:118
131.06538:400
131.40654:68
132.08139:2647
132.18411:92 133.03:51
133.08813:296
134.09421:85
140.04866:319
140.10263:66
141.05031:101
142.06618:3012
142.17102:156
142.2135:64 142.48039:78
143.07294:6672
143.17569:270
143.29416:81
143.38425:81
143.42514:54
143.93016:55
144.07979:3356
144.26543:58
145.08229:245
146.05989:2825
146.21466:67
147.06003:462
149.0546:60 157.06024:56
158.08319:146
159.09554:338
159.15872:57
170.0594:616
170.17122:54

										170.67471:57<br>171.06226:59<br>171.09094:51
1.228	102.05557	Threonine	[M+H-H2O]+	102.055	5.59	C4H9NO3	AYFVYJQAPQTCCC-UHFFFAOYSA-N	64.6	102.0553:13447<br>103.05865:0<br>104.06201:0	56.04462:113

7.134	218.14189	Propionylcarnitine	[M+H]+	218.1387	14.62	C10H19NO4	UFAHZIUFPNSHSL-MRVPVSSYSA-N	66.9	218.14189:505116 219.14524:80039 220.1486:10143	43.02213:117 44.02267:63 44.04715:111 44.05834:77 56.05146:185 57.03431:2036 57.0814:104 57.1168:63 58.03494:162 58.07043:151 59.07789:74 60.08113:1164 60.12985:57 60.15417:53 72.07825:64 74.06136:218 84.08365:286 84.11921:56 85.03054:13729 85.10996:598 85.13551:216 85.17271:134 85.25544:127 85.30837:54 85.37169:50 85.41438:60 85.44432:68 85.47034:90 85.49285:59 85.84096:74 86.03372:790 88.019:127 98.09823:70 103.03719:53 132.07014:70 140.0631:61 144.0948:96 161.06584:70 182.91376:89
11.18	116.07571	Proline	[M+H]+	116.0706	44.02	C5H9NO2	ONIBWKKTOPOVIA-UHFFFAOYSA-N	67.1	116.07169:21201 117.07504:1809 118.0784:771	43.05234:81 58.06629:102 68.05318:52 70.06694:443 70.11933:50
4.226	130.0874	Pipecolic acid	[M+H]+	130.0863	8.46	C6H11NO2	HXEACLLIILLPRG-YFKPBYRVSA-N	63.2	130.0874:57299 131.09075:4868 132.09411:723	42.03178:60 55.05396:131 56.04907:815 84.08081:934 84.13683:86 97.00168:117
12.56	180.10298	Phenylalanine, methyl ester	[M+H]+	180.1019	6.00	C10H13NO2	VSDUZFOSJDMAFZ-UHFFFAOYSA-N	62.7	180.10074:8941 181.10409:2738 182.10745:582	77.04062:95 91.05781:148 93.07916:62 103.0562:172 106.06483:61 120.08429:240

5.68	150.06169	Methionine	[M+H]+	150.0583	22.59	C5H11NO2S	FFEARJCKVFRZRR-BYPYZUCNSA-N	65	150.05823:309208 151.06158:21226 152.06494:19382	41.04028:240 44.04766:68 44.97964:427 45.98795:146 46.02575:87 46.96988:62 46.99421:135 49.00688:136 53.0379:153 56.04979:4416 56.11695:128 56.15549:81 56.19213:61 57.05213:227 58.99752:57 61.01124:5394 61.08097:213 61.19873:62 62.01412:225 63.01302:143 71.99899:67 74.02495:157 77.00392:59 85.01051:77 87.02928:168 88.02125:61 96.08092:69 98.10188:88 111.08472:53 125.10408:96

3.947	147.11705	Lysine	[M+H]+	147.11279	28.96	C6H14N2O2	KDXKERNSBIXSRK-YFKPBYRVSA-N	69.3	147.11433:485189 148.11768:40972 149.12104:3211	40.02656:90 41.03813:967 41.09379:56 42.03512:1158 42.09159:51 43.0192:151 43.04392:103 44.0462:67 46.03455:69 55.05571:1436 55.12313:58 56.05012:8698 56.11761:325 56.21106:51 56.31717:51 56.36604:67 57.05152:294 57.06667:257 58.07233:69 65.03943:346 67.05408:1324 67.11102:57 68.05818:384 69.05898:385 70.06116:160 74.02385:224 82.04034:165 82.06258:349 82.0927:89 84.08217:13665 84.16231:509 84.19748:160 84.23772:121 84.28613:100 84.32957:92 84.37553:107 84.43094:88 84.45639:65 84.48668:63 84.64126:69 84.73434:60 85.08488:1111 85.16656:77 107.04031:85 108.05013:61 109.05069:210 119.03389:70 135.05353:88

8.797	132.10338	Leucine	[M+H]+	132.1019	11.20	C6H13NO2	ROHFNLRQFUQHCH-YFKPBYRVSA-N	68.4	132.10254:1070127 133.10589:87578 134.10925:7671	41.04007:4599 41.09476:152 42.0437:190 42.6097:64 43.05542:8764 43.11238:377 43.13512:145 43.14891:95 43.20858:55 43.24496:57 43.28622:50 44.05153:11020 44.10969:521 44.12992:144 44.16374:87 44.21144:88 44.24863:64 45.05208:729 55.0172:237 55.05319:415 69.07537:182 73.0601:55 86.09774:566 86.17113:72
8.288	132.10632	Isoleucine	[M+H]+	132.1019	33.46	C6H13NO2	AGPKZVBTJJNPAG-UHFFFAOYSA-N	71.5	132.1013:744357 133.10465:54021 134.10801:5165	40.03126:81 41.03884:7963 41.09546:283 41.15598:61 41.23664:72 42.03873:561 43.01479:90 43.05311:140 44.04934:8149 44.10897:412 44.12925:130 44.15442:51 44.18075:62 44.22876:88 45.04168:786 45.1124:59 55.05302:97 55.98888:74 56.05021:1664 56.08526:136 56.10973:104 57.0569:3482 57.12542:120 57.14436:55 58.06546:522 58.09241:112 59.04853:143 67.05669:117 69.03398:72 69.06951:1851 69.14529:63 70.06984:419 71.07297:123 73.06594:112

										86.09672:980 86.15041:53 87.10301:75
3.951	129.10332	Lisdexamfetamine	[M+H-C9H13N]+	129.1022	8.68	C15H25N3O	VOBHXZCDAVEXEY-JSGCOSHPSA-N	68.1	129.10336:8509 130.10671:147712 131.11007:9479	41.04119:174 42.03362:197 53.04162:65 56.05021:1277 57.06683:79 65.02959:56 67.05385:154 68.05117:80 69.05799:115 82.06473:146 84.08074:1368 85.0834:80
4.656	116.07166	Hydroxyarginine	[M+H-CH5N3O]+	116.0706	9.13	C6H14N4O3	FQWRAVYMZULPNK-BYPYZUCNSA-N	64.4	116.07166:13898 117.07501:863 118.07837:1158	70.06712:497 70.0982:90
1.393	120.06792	Homoserine	[M+H]+	120.0655	20.16	C4H9NO3	UKAUYVFTDYCKQA-VKHMYHEASA-N	65.6	120.0677:6426 121.07105:564 122.07441:0	44.04729:57 56.04768:200
3.836	156.07881	Histidine	[M+H]+	156.0768	12.88	C6H9N3O2	HNDVDQJCIGZPNO-YFKPBYRVSA-N	71.3	156.07823:260376 157.08158:20843 158.08494:1472	41.03825:278 42.03978:87 42.43792:50 43.04379:79 54.03357:187 55.04394:162 55.87814:65 56.04969:2374 56.11421:79 57.05264:163 66.03418:606 68.04672:136 69.05264:115 71.04937:87 81.04646:1327 81.15623:68 82.05346:1595 82.13232:57 82.19868:51 83.06164:5189 83.14445:238 83.17742:95 84.06443:308 93.04594:2441 93.12605:133 94.04535:104 95.05792:171 109.79744:52

										110.07339:1149 111.07268:236
1.913	147.07895	Glutamine	[M+H]+	147.0764	17.34	C5H10N2O3	ZDXPYRJPNDTMRX-VKHMYHEASA-N	60.8	147.07594:21404 148.07929:15610 149.08265:1053	41.03629:83 44.05299:285 45.0532:54 56.04746:120 58.06613:69 84.04352:328
1.763	148.06125	Glutamic acid	[M+H]+	148.06039	5.81	C5H9NO4	WHUUTDBJXJRKMK-VKHMYHEASA-N	68.9	148.06071:229498 149.06406:15828 150.06742:2693	41.03846:1494 41.0813:84 41.11436:53 42.0416:86 56.04925:2419 57.05008:145 58.06145:80 70.06585:58 74.02131:109 84.04459:1672 84.10641:102 85.04759:363
11.536	461.33945	Leu-Val	[2M+H]+	461.3334	13.11	C11H22N2O3	MDSUKZSLOATHMH-IUCAKERBSA-N	61.7	461.33554:30675 462.33889:9507 463.34225:1879	69.07109:177 72.08523:76 86.09848:5175 86.18048:192 86.21274:114 86.39035:61 87.10057:845 87.18468:51 118.10427:77 145.07571:60 154.04855:64 158.0938:62 185.17171:68 231.1803:246 245.8925:55
4.121	201.12424	Leu-Ser	[M+H-H2O]+	201.1234	4.18	C9H18N2O4	XGDCYUQSFDQISZ-BQBZGAKWSA-N	61	201.12276:3239 202.12611:934 203.12947:31108	53.03629:103 60.04531:944 70.02612:75 70.06546:1356 71.07144:220 77.03871:58 116.06381:124
11.768	229.15863	Leu-Pro	[M+H]+	229.15469	17.19	C11H20N2O3	VTJUNIYRYIAIHF-IUCAKERBSA-N	68	229.15642:594200 230.15977:80432 231.16313:9013	41.03978:160 43.05494:850 43.11186:72 44.05064:1443 44.1093:64 44.17604:53 70.0675:8540 70.13969:252 70.16588:167 70.28007:67 70.43905:61 71.07181:349 86.0985:2561 86.17886:88 87.09713:124 88.10284:70

										116.07402:2217<br>116.17073:109
13.398	489.35556	Leu-Leu	[2M+H]+	489.36469	18.66	C12H24N2O3	LCPYQJIKPJLB-<br>ZJUUUORDSA-N	66	489.36237:11570<br>490.36572:5438<br>491.36908:1296	69.07314:64<br>86.09553:3253<br>86.17034:127<br>87.09609:124<br>113.09293:69<br>132.10316:75<br>199.17398:138<br>245.19185:253
14.528	269.14938	Leu-His	[M+H]+	269.1608	42.43	C12H20N4O3	XWOBNBRUDDUE<br>EY-NXEZZACHSA-N	67.8	269.16251:13552<br>270.16586:2776<br>271.16922:1286	56.04846:72 83.05785:332<br>86.09562:97 93.04306:270<br>95.02037:58<br>110.06923:949<br>111.06635:150<br>149.05925:66
11.053	189.12827	Leu-Gly	[M+H]+	189.1234	25.75	C8H16N2O3	LESXFEZIFXFIQR-<br>UHFFFAOYSA-N	64.4	189.12476:49762<br>190.12811:5996<br>191.13147:629	86.09528:204
12.765	288.20139	Leu-Arg	[M+H]+	288.203	5.59	C12H25N5O3	SENJXOPIZNYLHU-<br>RKDXNWHRSA-N	66.7	288.2048:72824<br>289.20815:14114<br>290.21151:1711	44.05041:167<br>60.05687:556<br>60.07976:253 61.05775:95<br>69.07362:202<br>70.06868:1348<br>70.12699:61 71.07095:61<br>72.08462:71 86.09962:779<br>86.1587:52 87.09913:127<br>97.076:90 112.09277:116<br>113.08583:100<br>116.07326:302<br>116.20543:57<br>117.07816:72<br>130.11604:87<br>158.09552:170<br>175.12099:172<br>176.12042:57

3.264	223.07675	Cystathionine	[M+H]+	223.07471	9.14	C7H14N2O4S	ILRYLPWNYFXEMH-RFZPGFLSSA-N	65.9	223.07675:100525 224.0801:17039 225.08346:12172	44.04918:124 46.02986:66 56.05024:1154 61.00963:114 74.02345:267 74.0596:135 88.02177:4451 88.10634:191 88.1262:76 89.02547:145 90.01762:182 102.05625:108 124.03908:56 134.02621:176
2.014	162.11456	Carnitine	[M+H]+	162.1125	12.71	C7H15NO3	PHIQHXFUZVPYII-ZCFIWIBFSA-N	72.2	162.11456:430284 163.11791:38026 164.12127:3581	41.03911:377 43.01908:3068 44.0504:336 45.05804:689 57.03402:884 58.06494:1423 58.12925:93 59.07216:673 60.08126:1944 60.15273:91 61.02809:169 73.04842:50 78.02944:57 85.03333:556 102.09347:845 102.16602:51 103.03902:326
1.556	134.04483	Aspartic acid	[M+H]+	134.0448	0.22	C4H7NO4	CKLJMWTZIZZHCS-REOHCLBHSA-N	63.5	134.0461:15838 135.04945:593 136.05281:0	43.02161:184 44.05121:93 46.02771:92 74.02657:50
5.557	130.08656	Arginine, methyl ester	[M+H-CH5N3]+	130.0863	2.00	C7H16N4O2	ZDXNCMJBOYJV-YFKPBYRVSA-N	64.3	130.08769:10939 131.09104:1050 132.0944:0	70.06783:122

6.463	175.12065	Arginine	[M+H]+	175.119	9.42	C6H14N4O2	ODKSFYDXXFIFQN-BYPYZUCNSA-N	68.6	175.11905:1028530 176.1224:101474 177.12576:8317	41.03962:364 43.03101:956 43.05537:1293 43.11283:60 44.03584:221 44.05935:145 55.02887:157 55.05285:84 56.03426:50 60.05709:2826 60.1274:86 60.14982:61 61.0567:154 68.05071:695 69.03471:107 69.72744:58 70.02916:75 70.06695:38838 70.14028:1924 70.22219:115 70.26053:222 70.3205:78 70.34708:72 70.43152:71 70.51375:75 70.54519:52 70.61765:67 70.66637:72 70.70923:76 70.76431:79 71.06693:2477 71.14228:84 71.29501:73 72.04922:86 72.08333:505 73.07024:57 74.02734:190 84.08414:125 86.09393:76 95.07044:72 97.0785:67 98.06287:198 115.09196:216 116.07112:186 118.92603:59 118.95603:52 130.09836:219
10.245	203.14244	Alanylnorleucine	[M+H]+	203.13901	16.88	C9H18N2O3	RSIYBPVKRVHPQK-UHFFFAOYSA-N	61.9	203.13997:51842 204.14332:6144 205.14668:1077	41.0378:128 44.05004:834 45.05352:63 69.06909:408 86.09724:757 86.14993:63 87.09759:55 109.04564:79

2.598	277.10571	γ-Glutamylglutamic acid	[M+H]+	277.103	9.78	C10H16N2O7	OWQDWQKWSLFFFR-WDSKDSINSA-N	62.4	277.10562:42736 278.10897:6390 279.11233:810	41.04222:68 56.05294:141 57.02993:54 84.04668:1287 85.03649:100 102.0529:98 115.03579:94 130.05066:85 136.04208:60 149.05647:79
4.026	144.10356	L-β-Homolysine	[M+H-NH3]+	144.1019	11.52	C7H16N2O2	PJDINCOFOROBQW-LURJTMIESA-N	66	144.10321:23782 145.10656:3693 146.10992:3872	41.04019:634 42.03522:700 42.06889:60 43.01643:113 43.04461:241 44.04964:137 55.05603:952 56.05213:7731 56.11195:296 56.1422:119 56.16928:70 56.21127:84 56.23831:90 56.43476:82 57.03583:80 57.06898:171 58.06164:97 65.03793:298 67.05663:1742 67.12988:97 68.05221:234 69.06081:743 69.10078:66 74.0248:67 82.06886:255 82.10082:56 84.08429:16432 84.16412:686 84.1935:280 84.23666:170 84.34631:115 84.43114:125 84.50006:69 84.63417:62 84.66437:82 84.84768:64 85.08122:83 98.09583:91

12.302	246.17247	Isovalerylcarnitine	[M+H]+	246.17	10.03	C12H23NO4	IGQBPDJNUXPEMT-SNVBAGLBSA-N	67.3	246.17247:2303454 247.17582:434988 248.17918:58550	41.04139:1868 41.08247:121 41.09798:56 42.04212:161 43.02067:230 43.05555:77 44.05138:262 45.06014:332 57.03703:7907 57.07298:13018 57.14009:585 57.16349:234 57.19561:167 57.23346:167 57.3091:89 57.43364:57 57.51246:53 57.6738:63 57.80603:52 57.95737:66 58.04155:204 58.07221:1434 58.13853:94 58.26577:57 58.35019:63 59.07525:584 59.26291:95 60.08437:7553 60.15228:287 60.17159:118 60.19467:51 60.23022:87 60.39358:58 60.99008:55 61.03385:228 61.09:559 83.14201:78 84.08511:1905 84.18896:54 84.46867:117 84.59404:57 84.72863:67 84.75533:65 84.79854:66 84.87317:55 85.03321:132459 85.11276:9375 85.50996:85 85.54721:123 85.59026:106 85.68156:107 85.72456:106 85.75204:87 85.77585:91 85.83241:73 85.85475:90 85.8902:123 85.91208:94 85.94531:63 86.03677:6396

										86.11787:287 86.14487:117 86.17639:125 86.23451:83 86.29674:61 86.33807:109 86.39422:69 86.41766:57 86.48383:65 86.50632:55 86.61379:100 86.68466:56 86.74207:78 86.75848:70 86.89024:80 87.03786:830 87.10484:69 87.15585:59 87.22612:58 89.6102:53 96.0854:68 98.09673:95 103.04055:95 103.08319:282 105.08705:57 129.08519:492 144.10852:1806 144.26768:65 145.11339:186 162.12146:181
12.81	132.08141	Indapamide	[M+H-C7H7ClN2O3S]+	132.0808	4.62	C16H16ClN3O3S	NDDAHWYSQHTHNT-UHFFFAOYSA-N	63.9	132.08205:8246 133.0854:1219 134.08876:0	117.0619:75
11.537	231.17419	Ile-Val	[M+H]+	231.1703	16.83	C11H22N2O3	BCXBIONYYJCSDF-CIUDSAMLSA-N	67.2	231.17419:458707 232.17754:71157 233.1809:8039	41.04092:1105 41.07592:76 43.0579:89 44.05178:2557 44.1088:63 44.30324:60 44.43355:61 45.05583:188 56.04962:229 56.96447:64 57.05663:207 67.061:57 69.07231:3330 69.1441:135 69.26608:52 70.0759:305 72.08518:764 72.29193:59 74.05829:61 85.65752:54 86.10015:6486 86.20309:137 86.23961:76 86.35312:63 87.10474:338 118.91988:99

6.719	233.14995	Ile-Thr	[M+H]+	233.1496	1.50	C10H20N2O4	DRCKHKZYJYFQ-YWIQKCBGSA-N	63.1	233.15056:62673 234.15391:7795 235.15727:0	41.03584:218 44.04996:282 57.05965:55 67.0526:56 69.06785:404 86.09619:699 87.09707:70 118.92463:204 159.04739:79 165.9733:88
6.183	219.13454	Ile-Ser	[M+H]+	219.1339	2.92	C9H18N2O4	TWVKGYNQQAUNRN-ACZMJKKPSA-N	67.4	219.13301:63424 220.13636:7762 221.13972:1348	41.03777:427 41.05672:110 44.05147:567 44.07538:134 58.06674:102 69.06957:750 70.06293:52 75.067:94 86.09776:690 87.09695:126 104.94244:69 191.1429:53
11.177	229.16508	Ile-Pro	[M+H]+	229.15469	45.34	C11H20N2O3	BBIXOODYWPFNDT-CIUDSAMLSA-N	69.9	229.1552:516490 230.15855:70873 231.16191:7322	41.03905:818 41.08678:64 44.04979:1832 44.10433:59 45.05295:115 55.05104:57 56.05251:108 57.06027:222 58.06441:271 58.99408:65 69.07146:2593 69.12304:169 69.17368:78 70.06684:7578 70.13559:229 70.16225:125 70.27633:54 70.52676:51 70.9544:66 71.0682:567 72.24392:53 86.09724:3528 86.17351:86 87.10232:258 116.07104:2063 117.07732:102 122.94785:53 135.08669:54

12.081	260.19666	Ile-Lys	[M+H]+	260.1969	0.92	C12H25N3O3	UWBNOCIDGPQE-LPEHRKFASA-N	66.5	260.19858:170475 261.20193:28652 262.20529:3240	41.03984:299 44.04902:84 56.04562:59 67.05515:178 69.07345:315 83.66894:73 84.04385:54 84.08221:2959 84.15842:121 85.08527:114 85.10703:53 86.0972:1482 86.16716:104 87.10511:108 91.04949:94 107.05024:73 107.08826:53 121.06097:70 129.1076:170 130.08734:593 147.11247:55 149.09268:73 182.91817:80 219.93713:59 245.16225:112
13.738	245.18016	Ile-Leu	[M+H]+	245.186	23.82	C12H24N2O3	JWBXCSQZLLIOCI-LPEHRKFASA-N	63.4	245.18176:212657 246.18511:38371 247.18847:4640	41.03645:185 42.03741:53 43.01868:172 43.0487:88 44.04905:452 45.05708:85 52.47237:52 57.05547:90 58.06891:59 67.05203:71 69.06798:722 69.12154:57 70.07027:69 85.02624:77 86.09434:2025 86.17671:81 87.09709:56 91.04749:199 93.07178:57 97.05621:54
12.196	269.16232	Ile-His	[M+H]+	269.1608	5.65	C12H20N4O3	QNBYCZTZNOVDMI-FXBDTBDDSA-N	66.8	269.16412:34766 270.16747:6806 271.17083:1343	41.04002:78 56.04884:76 57.03696:64 69.06989:98 72.08492:90 83.05712:87 83.08385:69 86.09588:226 86.12511:70 93.04816:57 95.05502:244 95.08612:53 110.07151:2077 110.14115:65 111.073:126

										121.05789:77 139.04988:64 155.94077:58 156.07513:174 200.91061:73
7.532	189.12624	Ile-Gly	[M+H]+	189.1234	15.02	C8H16N2O3	UCGDDTHMMVWVMV-FSPLSTOPSA-N	65	189.1259:107716 190.12925:11394 191.13261:2025	41.03768:315 44.04937:481 55.0545:204 57.05707:199 69.06887:515 84.08543:65 86.09448:556 86.13959:70
7.409	261.14478	Ile-Glu	[M+H]+	261.1445	1.07	C11H20N2O5	KTGFOCFYOZQVRJ-ACLDMZEESA-N	64	261.14648:144392 262.14983:41831 263.15319:5749	41.03837:263 44.04947:304 45.02799:51 45.04773:74 55.05846:73 56.05064:105 57.03273:98 58.06383:109 69.07058:685 69.12125:59 70.07001:69 84.0478:280 85.03072:712 86.09705:2241 86.17995:66 87.0955:150 102.05722:53 130.04579:53 144.09897:66 148.05638:74 149.04222:91 192.98679:76
6.985	260.16037	Ile-Gln	[M+H]+	260.16049	0.46	C11H21N3O4	CNPNWGHRMBQHBZ-ACLDMZEESA-N	61.2	260.1608:48105 261.16415:8575 262.16751:3932	41.03803:141 43.05201:125 44.04972:176 44.06339:78 45.04834:61 46.06409:80 69.02876:98 70.06744:54 84.04475:1005 85.04907:182 86.09711:829 87.10244:104 102.93959:58 130.04916:438 131.04474:69 182.90245:99

7.668	203.14497	Ile-Ala	[M+H]+	203.13901	29.34	C9H18N2O3	RCFDOSNHHZGBOY-ACZMJKKPSA-N	64.7	203.14247:185614 204.14582:25753 205.14918:2720	41.0395:402 44.05037:603 57.06802:66 69.07037:773 85.02667:68 86.09831:1239 86.18163:61 87.09953:137
12.969	255.14706	His-Val	[M+H]+	255.1452	7.29	C11H18N4O3	VLDVBZICYBVQHB-IUCAKERBSA-N	68.4	255.14629:75925 256.14964:12198 257.153:1431	55.05436:87 56.04842:141 56.06613:90 66.03566:99 67.05569:52 68.04738:78 81.04253:117 82.05036:115 83.06149:1101 84.06809:75 93.0458:561 110.07142:3468 110.16564:101 111.07627:167 113.07538:78 164.11682:119 175.07571:60
14.966	342.15707	His-Trp	[M+H]+	342.1561	2.83	C17H19N5O3	FBTYOQIYBULKEH-ZFWWWQNUSA-N	60	342.15567:11978 343.15902:3587 344.16238:0	60.08094:53 70.06289:52 83.06326:69 84.07616:60 86.10032:57 87.09364:60 93.04631:65 105.06458:52 106.90231:88 110.07146:1144 111.07573:66 170.05026:52 252.18443:60
10.239	253.13098	His-Pro	[M+H]+	253.1295	5.85	C11H16N4O3	LNCFUHAPNTYMJB-IUCAKERBSA-N	69.1	253.13141:86913 254.13476:12971 255.13812:5383	56.04919:253 57.05536:82 70.06364:284 81.0457:103 82.05238:100 82.7178:86 83.05966:1498 83.14132:87 84.0661:155 93.04523:1369 94.04597:163 95.06487:78 109.06184:57 110.0723:5664 110.16292:148 110.23589:66 111.0741:502

										116.06988:63 122.06414:61 167.02852:68
12.889	284.17639	His-Lys	[M+H]+	284.17169	16.54	C12H21N5O3	CZVQSYNVUHAILZ-UWVGGRQHSA-N	61.8	284.17453:24599 285.17788:5717 286.18124:1068	81.07293:96 84.08479:78 91.05821:76 93.03711:84 95.05427:66 110.07039:555
14.279	269.15176	His-Ile	[M+H]+	269.1608	33.59	C12H20N4O3	IDXZDKMBEXLFMB-HGNGGELXSA-N	66.8	269.16287:53731 270.16622:10301 271.16958:1487	56.04653:349 66.02747:53 69.06976:53 83.05433:1233 93.03942:563 94.04516:83 95.05052:69 110.06515:3513 110.15727:125 110.19118:52 111.07165:332 123.04897:66 178.12451:65
13.067	293.13766	His-His	[M+H]+	293.13571	6.65	C12H16N6O3	SGCGMORCWLEJNZ-UWVGGRQHSA-N	60.5	293.13766:21913 294.14101:4700 295.14437:1250	56.04917:98 58.96055:751 59.05723:54 83.06764:200 93.04208:118 95.0611:95 107.06084:53 110.07128:1003 111.08002:61 152.90257:63
6.506	213.09856	His-Gly	[M+H]+	213.09821	1.64	C8H12N4O3	LYCVKHSJGDMM-LURJTMIESA-N	66	213.09764:35166 214.10099:4051 215.10435:0	56.04898:76 66.03334:79 70.06346:50 72.08123:147 83.06009:824 93.04353:496 94.04693:97 97.06895:54 110.07175:1742 110.16132:55 111.07352:73
7.718	285.12433	His-Glu	[M+H]+	285.11929	17.68	C11H16N4O5	VHOLZZKNEBBHTH-YUMQZZPRSA-N	63.6	285.12277:34941 286.12612:5812 287.12948:790	56.04882:96 70.06428:56 83.05805:531 93.04468:428 94.04674:98 110.07227:1771 111.07642:84 114.0547:58

										124.07588:132 194.08975:58
6.652	271.10638	His-Asp	[M+H]+	271.1037	9.89	C10H14N4O5	MDCTVRUPVLZSPG-BQBZGAKWSA-N	60.5	271.10672:12385 272.11007:1564 273.11343:553	84.08381:64 93.04081:75 110.07033:431 132.03331:59
5.644	270.11499	His-Asn	[M+H]+	270.11969	17.40	C10H15N5O4	WSDOHRLQDGAOGU-BQBZGAKWSA-N	63.4	270.12177:27394 271.12512:4294 272.12848:780	82.06399:71 83.06104:324 93.04528:81 94.04807:85 110.07228:1453 110.14357:59 110.80051:66 111.07226:153
8.155	227.11371	His-Ala	[M+H]+	227.11391	0.88	C9H14N4O3	FRJIAZKQGSCKPQ-FSPLSTOPSA-N	67.6	227.1142:35486 228.11755:4826 229.12091:0	41.03847:51 55.0586:54 56.04946:185 58.02402:54 58.06385:54 59.077:56 81.0397:57 82.05111:53 83.06184:895 85.02838:276 93.04623:280 95.06351:84 107.07236:122 110.0724:1388 111.07381:118
5.878	201.11386	Hexanoylcarnitine	[M+H-C3H9N]+	201.11211	8.70	C13H25NO4	VVPRQWTYSNDTEA-LLVKDONJSA-N	64.4	201.11264:7032 202.11599:784 203.11935:2459	57.03222:258 58.06546:60 59.07067:112 85.02817:1150 85.70959:65 108.04447:66
2.086	364.06693	Guanosine 5'-tetraphosphate	[M+H-H3P3O9]+	364.06531	4.45	C10H17N5O17P4	KDFXXNGTULMGMF-BDXYJKHTSA-N	60.3	364.06662:6992 365.06997:1295 366.07333:0	84.05597:52 85.08038:51 110.03365:82 135.03296:75 152.06119:390 154.07791:75

2.349	364.06464	Guanosine 5'-monophosphate	[M+H]+	364.06531	1.84	C10H14N5O8P	RQFCJASXJCIDSX-UUOKFMHZSA-N	67.3	364.06464:61077 365.06799:8124 366.07135:1763	43.03066:98 55.03476:63 85.02755:118 97.02673:301 98.98354:214 109.05127:128 110.0334:344 135.03104:851 136.02834:51 137.03224:91 152.0558:4978 152.17085:134 152.21585:62 152.52007:65 153.05769:724 153.39882:61 187.97499:62
5.287	284.10211	Guanosine	[M+H]+	284.099	10.95	C10H13N5O5	NYHBQMYGNKIUIF-UUOKFMHZSA-N	69.7	284.09808:344584 285.10143:46117 286.10479:7822	43.02302:279 45.03516:124 55.02495:307 57.03563:294 61.02468:52 69.0334:126 71.0109:131 80.02468:138 82.03694:342 85.02576:132 109.05048:382 110.03483:2867 110.15516:51 111.03493:172 115.03145:74 124.04051:128 134.04703:140 135.02913:10325 135.12885:371 135.16681:215 135.24919:120 135.33034:100 135.37727:93 135.4594:85 135.57687:63 135.67809:57 135.75444:53

										135.89546:53<br>136.03125:765<br>136.13768:64<br>136.30804:55<br>152.05508:23770<br>152.16055:1037<br>152.20432:404<br>152.26164:225<br>152.31015:192<br>152.36873:216<br>152.41139:186<br>152.46596:156<br>152.60583:106<br>152.80431:90<br>152.91646:58<br>153.05525:2138<br>153.16151:61<br>153.19835:55<br>153.51387:66<br>153.57683:65<br>153.64877:88<br>153.89825:56<br>154.06476:160<br>155.06752:83
5.292	152.05641	Guanine	[M+H]+	152.0567	1.91	C5H5N5O	UYTPUPDQBNUYG<br>X-UHFFFAOYSA-N	70	152.05688:237371<br>153.06023:18635<br>154.06359:1463	40.01867:131 40.03873:52<br>41.01228:79 43.02972:514<br>53.01561:176<br>55.02845:701<br>65.01214:201 66.0166:100<br>68.02084:149<br>80.02293:572<br>82.03982:313 83.03162:66<br>93.0087:112 98.07881:61<br>110.03599:647<br>111.03056:56<br>134.04424:134<br>135.02986:1107<br>136.03137:68

6.456	60.05751	Guanidine	[M+H]+	60.0556	31.80	CH5N3	ZRALSGWEFCBTJO-UHFFFAOYSA-N	63	60.05751:7330 61.06086:0 62.06422:0	43.02949:113
13.433	313.20261	Granisetron	[M+H]+	313.2023	0.99	C18H24N4O	MFWNKCLOYSRHCJ-BTTYYORXSA-N	61.2	313.20056:4219 314.20391:2392 315.20727:581	87.04014:80 97.09199:73 99.08094:88 120.07675:66 121.07221:58 138.11414:55 313.19778:173
5.169	175.10999	Gly-Val	[M+H]+	175.1077	13.08	C7H14N2O3	STKYPAFSDFAEPH-LURJTMIESA-N	69.8	175.10803:50066 176.11138:4204 177.11474:524	55.05417:1144 56.05701:78 57.05716:218 72.08199:651 73.08213:54
1.091	258.11066	Glycerophosphocholine	[M+H]+	258.11011	2.13	C8H20NO6P	SUHOQUVVVLNYQR-MRVPVSSYSA-N	65.9	258.11066:335257 259.11401:44101 260.11737:6620	45.03266:272 45.05603:145 57.03051:58 58.06691:90 60.08249:543 61.08356:108 71.07455:158 86.09721:896 97.02742:94 98.98491:275 104.10778:1168 104.19907:58 124.99834:567 125.04691:55 166.06551:194 241.08054:66
7.395	232.14178	Gly-Arg	[M+H]+	232.1404	5.94	C8H17N5O3	JLXVRFDTDUGQEE-YFKPBYRVSA-N	65.4	232.14186:78596 233.14521:9826 234.14857:1494	43.02856:94 60.05642:116 70.06528:2136 70.13642:72 70.20454:81 71.04563:133 71.07093:176 72.07899:126 73.07016:53 74.01929:115 82.06812:60 84.04681:84 88.07806:100 95.06451:136 97.07253:152 98.06167:102 100.04993:152 112.0881:198 115.08862:102 116.06876:316

										116.10046:83 118.92158:150 130.09473:93 137.33598:80 158.10027:173
6.867	247.13098	Glu-Val	[M+H]+	247.1288	8.82	C10H18N2O5	SITLTJHOQZFJGG-XPUUQOCRSA-N	61	247.13098:55394 248.13433:26848 249.13769:4254	41.03991:345 44.0485:104 45.03361:276 50.54127:94 55.05345:186 56.04846:212 72.08014:973 73.07893:68 84.04646:1546 84.1218:68 84.18188:58 85.0284:687 85.09229:89 86.09384:281 102.05431:83 103.04166:80 118.08971:72 138.09526:80
8.665	311.12357	Glu-Tyr	[M+H]+	311.12381	0.77	C14H18N2O6	YSWHPLCDIMUKFE-UHFFFAOYSA-N	64.3	311.11896:12860 312.12231:2649 313.12567:0	70.06258:105 72.07739:55 84.04646:437 91.05109:198 102.05056:102 107.05633:54 119.05249:130 123.04681:56 136.07452:607 147.04648:141 148.04637:51 165.05522:200
13.136	334.1412	Glu-Trp	[M+H]+	334.13971	4.46	C16H19N3O5	LLEUXCDZPQOJMY-DGCLKSJQSA-N	61.5	334.13885:34911 335.1422:8984 336.14556:8331	58.9597:914 70.07027:94 84.04376:459 103.05764:58 125.07294:141 130.06125:352 132.07901:302 136.06174:606 136.9288:68 142.05556:60 144.07634:106 146.05405:586

										153.05687:57<br>159.08995:165<br>170.06252:262<br>188.06827:640<br>189.06857:130<br>204.11121:145<br>205.11774:67<br>225.09001:64
2.23	249.10918	Glu-Thr	[M+H]+	249.10809	4.38	C9H16N2O6	JSIQVRIXMINMTA-ZURKLDSA-N	66.1	249.11029:20954<br>250.11364:3819<br>251.117:0	56.04879:146<br>74.05983:322<br>84.04647:408<br>102.05569:200
5.839	245.11307	Glu-Pro	[M+H]+	245.1132	0.53	C10H16N2O5	YBTCBQBIJKGSJP-BQBZGAKWSA-N	69.8	245.11432:12725<br>246.11767:0<br>247.12103:1413	41.03749:121<br>56.05152:143 60.07997:87<br>69.04427:50 70.06599:928<br>83.05843:58 84.04425:908<br>85.02735:150 86.09949:59<br>116.06942:231
11.132	261.14667	Glu-Ile	[M+H]+	261.1445	8.31	C11H20N2O5	SNFUTOCQQRQD-LKEWCRSYSA-N	69.3	261.14673:41639<br>262.15008:5715<br>263.15344:1256	41.03879:274<br>44.04775:150<br>56.05201:626 60.07764:77<br>69.06927:93<br>84.04568:2123<br>84.11808:94 85.04731:71<br>86.09598:925<br>87.10061:190<br>102.04994:189
7.199	285.12115	Glu-His	[M+H]+	285.11929	6.52	C11H16N4O5	HKTRDWYCAUTRRL-HTQZYQBOSA-N	63.5	285.121:26471<br>286.12435:4457<br>287.12771:689	41.03977:131<br>83.05909:131<br>84.04439:195<br>95.06108:103<br>102.05261:54<br>110.07174:1410<br>111.07235:116<br>111.10002:62<br>122.06694:99<br>150.09274:94

									156.07555:136 176.07863:70
2.205	205.08629	Glu-Gly	[M+H]+	205.08189	21.45	C7H12N2O5	LSPKYLAFTPBWIL-BYPYZUCNSA-N	61	205.08624:26467 206.08959:3081 207.09295:683	41.04083:114 44.04701:113 56.05045:92 84.043:516 100.0584:53 130.04588:50
2.044	276.11841	Glu-Gln	[M+H]+	276.11899	2.10	C10H17N3O6	MGHKSHCBDXNTHX-RITPCOANSA-N	61.2	276.11841:20116 277.12176:2817 278.12512:1242	41.03475:56 84.04376:684 102.05595:131 130.05217:100 136.93048:52
6.794	246.14828	Gln-Val	[M+H]+	246.14481	14.10	C10H19N3O4	MRVYVEQPNDSWLH-XPUUQOCRSA-N	65.9	246.14828:46366 247.15163:11533 248.15499:7213	41.0407:174 42.04002:105 54.04283:66 55.05574:163 56.04966:267 57.05791:113 72.08118:813 72.14898:53 73.08391:65 83.059:279 84.04378:950 85.03249:92 138.10158:63 150.90721:118
8.433	310.14056	Gln-Tyr	[M+H]+	310.13971	2.74	C14H19N3O5	KGNSGRRALVIRGR-GHMZBOCLSA-N	60.4	310.14056:24896 311.14391:5283 312.14727:0	58.96259:71 66.02991:81 70.06597:177 74.09541:52 83.06126:392 84.04356:583 101.04052:59 121.06618:60 123.03716:128 126.05479:92 136.07521:164 137.07098:60 165.05122:118
2.029	248.12361	Gln-Thr	[M+H]+	248.1241	1.97	C9H17N3O5	HHSJMSCOLJVTCX-ZURKLDSA-N	66.3	248.12361:26747 249.12696:4282 250.13032:868	56.05303:74 74.06097:218 83.05859:254 84.04456:571 84.10503:53 130.04115:59

1.697	234.10712	Gln-Ser	[M+H]+	234.1084	5.47	C8H15N3O5	UKKNTTCNGZLJEX-WHFBIAKZSA-N	62.6	234.10808:74774 235.11143:8724 236.11479:1497	41.04158:117 42.03534:121 44.0506:70 56.04969:738 57.03584:57 59.03263:108 60.04444:54 70.02029:62 74.02161:75 74.05987:243 74.09524:56 83.06308:189 84.04383:1340 85.04394:76 97.02712:87 98.05344:52 98.07432:51 99.03627:110 106.06251:56 116.02377:58 126.07051:54
5.33	244.13222	Gln-Pro	[M+H]+	244.1292	12.37	C10H17N3O4	NJMYZEJORPYOTO-BQBZGAKWSA-N	72.2	244.12971:64351 245.13306:8732 246.13642:1564	41.04075:270 43.04123:54 56.04984:617 66.03697:130 66.06216:66 70.06583:2164 70.13798:54 71.07157:122 83.06173:2084 83.13992:77 84.04591:2881 84.14259:71 85.04648:114 101.07277:69 112.04909:69 116.07201:632 117.07647:68 129.06572:71
6.957	278.11865	Gln-Met	[M+H]+	278.11691	6.26	C10H19N3O4S	SIGGQAHUPUBWNF-BQBZGAKWSA-N	60.3	278.11829:18587 279.12164:3169 280.125:1919	56.04816:235 83.0622:91 84.01028:148 84.04272:681 104.04758:58 111.07092:58 131.05141:69 133.02904:77 168.06555:69

10.857	260.16385	Gln-Leu	[M+H]+	260.16049	12.92	C11H21N3O4	ARPVSMCNIDAQBO-SFYZADRCSA-N	67.9	260.16385:111735 261.1672:15771 262.17056:2417	41.0393:377 54.03963:61 56.04977:234 66.03065:97 83.06116:1575 83.14231:53 84.04649:2890 84.10297:195 84.1244:134 84.17517:50 85.046:237 86.09641:2429 86.17728:83 87.09789:115 101.07546:114 120.95487:72 129.05998:69 132.10744:154 182.8968:70
10.111	260.15985	Gln-Ile	[M+H]+	260.16049	2.46	C11H21N3O4	XITLYYAIPBBHPX-LKEWCRSYSA-N	64.9	260.16379:33091 261.16714:4490 262.1705:906	41.04167:75 69.07423:79 83.06161:648 84.04572:913 84.12569:63 86.09602:721 142.9585:53 200.91179:61
1.809	204.0988	Gln-Gly	[M+H]+	204.0979	4.41	C7H13N3O4	JEFZIKRIHOIF-BYPYZUCNSA-N	64.2	204.0988:26436 205.10215:2892 206.10551:2752	56.04668:261 58.06285:107 60.03898:88 70.02854:72 74.05891:54 83.0604:134 84.04402:899
1.908	275.13596	Gln-Gln	[M+H]+	275.13501	3.45	C10H18N4O5	LOJYQMFIIJVETK-RITPCOANSA-N	65.4	275.13461:51760 276.13796:9919 277.14132:1312	41.03949:72 56.04935:456 60.04222:66 70.06152:62 83.05966:1000 83.11705:62 84.04441:3201 84.1496:90 85.04705:133 98.06763:112 101.06956:135 102.05644:101 118.92493:61 129.0663:130 130.04793:496 131.0692:93 136.92538:62 167.07228:71 167.11072:54

										195.06366:80 214.09291:104
1.935	262.10623	Gln-Asp	[M+H]+	262.10339	10.84	C9H15N3O6	SSHIXEILTLPAQT-WHFBIAKZSA-N	68.5	262.10526:17556 263.10861:2622 264.11197:1342	41.04026:56 46.02909:291 56.04553:230 70.03162:101 74.02312:191 83.06009:227 84.04514:1275 88.03502:117 101.07147:105 116.02364:58
1.684	261.12106	Gln-Asn	[M+H]+	261.11929	6.78	C9H16N4O5	DXJZITDUDUPINW-WHFBIAKZSA-N	60.2	261.11951:17666 262.12286:3133 263.12622:1028	44.05014:73 56.05274:136 67.05145:100 74.02064:74 83.06127:258 84.04484:727 85.0493:91 87.05159:132 88.03514:62 96.04893:63 97.05118:53 98.01612:69 111.03452:71 113.06483:114 129.06851:57 139.05473:82
2.362	218.11311	Gln-Ala	[M+H]+	218.11349	1.74	C8H15N3O4	FAQVCWVVIYYWRR-WHFBIAKZSA-N	63.7	218.1153:33851 219.11865:13284 220.12201:1299	41.03738:105 44.04903:342 56.05341:177 57.03128:71 74.05717:99 83.06158:295 84.04504:793 101.07154:78
2.813	133.0618	Ethylmalonic acid	[M+H]+	133.0495	92.45	C5H8O4	UKFXDFUAPNAMPJ-UHFFFAOYSA-N	68.3	133.05034:3837 134.05369:0 135.05705:0	43.01743:124 45.03532:92
1.606	62.0602	Ethanolamine	[M+H]+	62.06	3.22	C2H7NO	HZAXFHJVJLSVMW-UHFFFAOYSA-N	67.2	62.06151:2131 63.06486:0 64.06822:0	42.03263:64 44.047:109 45.03275:220

1.577	230.09908	Ergothioneine	[M+H]+	230.09579	14.30	C9H15N3O2S	SSISHJJTAXXQAX-ZETCQYMHSA-N	63.4	230.09891:117841 231.10226:17509 232.10562:8396	41.03828:276 41.0581:80 42.0315:77 44.05106:120 56.04562:103 58.07206:119 58.99648:65 60.0822:346 67.04272:156 68.04871:410 69.05673:596 83.05831:142 84.0546:60 95.061:134 96.06181:63 100.01946:288 127.03094:807 129.02574:74
3.95	130.08989	Pipecolinic acid	[M+H]+	130.0863	27.60	C6H11NO2	HXEACLLIILLPRG-RXMQYKEDSA-N	69.8	130.08755:148219 131.0909:12219 132.09426:1065	41.03883:60 43.04469:56 55.05465:144 56.05154:1066 57.05359:123 67.05574:112 68.0638:79 69.05808:89 69.07487:65 82.0685:57 84.08425:1485
3.122	133.09892	Ornithine	[M+H]+	133.0972	12.92	C5H12N2O2	AHLPHDHHMVZTML-SCSAIBSYSA-N	65.4	133.09875:376713 134.1021:27532 135.10546:2673	43.01737:155 43.05478:716 44.0581:100 53.04051:121 58.06042:79 68.05147:173 68.08272:51 70.06618:7217 70.13803:267 70.16487:100 70.19067:98 70.26761:66 70.40656:58 71.06937:429
1.031	183.08617	Mannitol	[M+H]+	183.0863	0.71	C6H14O6	FBPFZTCFMRRESA-KVTDHHQDSA-N	60.7	183.08617:20765 184.08952:3830 185.09288:673	43.01755:103 45.03454:110 53.04179:90 53.05821:55 55.05094:62 69.03397:576 85.0452:66

10.548	166.09119	Phenylalanine	[M+H]+	166.0863	29.44	C9H11NO2	COLNVLDHVKWLRT-UHFFFAOYSA-N	71.2	166.0881:1333788 167.09145:163215 168.09481:14318	42.03474:1179 43.01678:75 43.03901:153 51.02208:1113 55.05917:56 65.03992:133 67.05562:96 76.38286:55 77.03938:20342 77.11559:802 77.15094:345 77.20178:149 77.25059:150 77.3186:99 77.38604:101 77.44771:78 77.54799:85 77.56898:67 77.86478:63 78.04227:1578 78.10362:101 79.05401:4892 79.13226:155 80.05208:508 91.05418:7200 91.13628:246 91.17113:91 92.05708:911 92.11341:81 92.15675:54 92.97358:65 92.99572:61 93.06932:5380 93.15093:200 93.22125:79 93.35571:69 94.06966:725 102.04521:944 102.11431:89 103.05445:44284 103.14165:2159 103.32021:143 103.38673:129 103.45084:91 103.50679:101 103.5484:64 103.61945:68 103.68944:64 103.80278:75 103.83044:60 103.86433:69 103.96684:63

										104.05724:4785<br>104.14104:145<br>104.17855:104<br>104.20298:68<br>104.2318:108<br>104.27966:76<br>104.31815:78 104.3431:54<br>104.45661:92<br>104.79941:52<br>105.06413:406<br>107.04802:243<br>117.04301:57<br>118.06441:697<br>119.07446:735 119.134:56<br>120.08067:18425<br>120.17595:855<br>120.20832:312<br>120.23794:215<br>120.27816:145<br>120.31069:142<br>120.33701:93<br>120.39368:112<br>120.52249:63<br>120.70444:55<br>120.87624:60<br>120.90711:84<br>120.96819:53<br>121.08163:1980<br>121.17714:105<br>121.2689:57
5.134	235.16628	Norvaline	[2M+H]+	235.16521	4.55	C5H11NO2	SNDPXSYFESPGGJ-<br>UHFFFAOYSA-N	62.3	235.16628:14485<br>236.16963:1497<br>237.17299:0	55.05645:475<br>56.05622:153<br>72.08194:2001 73.084:133<br>77.02093:71 118.08776:62

18.57	391.2843	Di-n-octyl phthalate	[M+H]+	391.2843	0.00	C24H38O4	MQIUGAXCHLFZKX-UHFFFAOYSA-N	65	391.2843:554029 392.28765:241814 393.29101:39656	41.0392:691 42.03756:60 43.05401:3393 43.11327:139 57.07011:5911 57.13773:269 57.20073:80 57.28115:70 57.40414:74 58.07151:550 65.03728:1061 69.07258:225 71.08536:3375 71.15791:133 71.1918:67 71.28494:52 72.08711:250 79.99824:68 81.0631:71 84.09212:81 85.09876:184 93.03221:859 95.08604:225 109.10165:355 120.04781:872 121.03411:1012 122.04296:580 122.13166:51 122.3078:69 123.04146:155 124.11578:65 125.05145:134 134.05861:66 135.05463:57 136.05411:221 137.06097:111 137.08463:82 148.51736:57 149.02254:43249 149.12604:2414 149.3521:69 149.44391:58 149.5499:185 149.76646:50 149.90163:67 150.0248:4827 150.13521:133 150.17175:93 150.24971:56

										150.43715:65 150.68257:57 151.02679:428 151.09854:67 167.02771:102 301.20718:72 301.24088:54 316.24704:87 359.27805:484 360.26718:608 361.27167:107 374.30533:123
18.501	259.1839	Dibutyl adipate	[M+H]+	259.1904	25.08	C14H26O4	XTJFFFGAUHQWII-UHFFFAOYSA-N	65.1	259.19446:29104 260.19781:6389 261.20117:748	55.05682:264 59.04999:103 83.05117:75 101.06824:143
18.57	149.02269	Di(5-nonyl) phthalate	[M+H-C18H38O]+	149.0233	4.09	C26H42O4	MGOWZWCOHIRWSL-UHFFFAOYSA-N	65.4	149.02287:191203 150.02622:22685 151.02958:2290	65.03854:5502 65.18745:64 65.27361:59 66.03996:376 93.03266:498 94.03593:96 121.02664:172
0.956	261.03952	Glucose 6-phosphate	[M+H]+	261.03699	9.69	C6H13O9P	NBSCHQHZLSJFNQ-DVKNGEFBSA-N	64.4	261.03952:23485 262.04287:1793 263.04623:11807	41.04029:84 43.01881:210 53.04197:285 55.01805:96 55.05276:114 61.03508:58 69.03249:631 81.03317:201 85.0258:85 98.98402:571 109.02821:189
14.106	257.17612	Decanoylcarnitine	[M+H-C3H9N]+	257.17471	5.48	C17H33NO4	LZOSYCMHQXPBFU-OAHLLOKOSA-N	63.9	257.17612:1481 258.17947:647 259.18283:0	43.05019:133 57.0341:315 60.07811:113 64.97323:86 67.05067:92 71.0798:73 79.04576:71 85.0273:1154 168.98561:50
4.789	112.05256	Cytidine 3'-monophosphate	[M+H-C5H9O7P]+	112.0505	18.38	C9H14N3O8P	UOOOPKANIPLQPU-AYZDMWBASA-N	64.9	112.05181:24730 113.05516:1705 114.05852:0	42.03881:112 52.01939:143 53.01088:90 67.03005:144 68.016:132 69.0468:58 95.03182:125

1.241	306.0498	Cytidine 2',3'-cyclic monophosphoric acid	[M+H]+	306.04861	3.89	C9H12N3O7P	NMPZCCZXCOMSDQ-XVFCMESISA-N	64.2	306.0498:8928 307.05315:1767 308.05651:0	69.04181:85 95.01672:77 112.04979:351 190.10344:53
3.541	244.09483	Cytarabine	[M+H]+	244.0928	8.32	C9H13N3O5	UHDGCWIWMRVCDJ-CCXZUQQUSA-N	66.5	244.09317:99268 245.09652:21016 246.09988:3803	42.03571:79 43.02113:116 45.03341:131 57.0318:77 58.95726:53 69.04548:728 70.06577:1199 71.0188:152 85.02836:201 94.0386:522 95.02227:1301 96.0252:131 97.02335:79 112.05018:11987 112.14227:549 112.1729:218 112.23959:102 112.30194:55 112.36659:60 112.42844:120 112.54703:54 112.97548:54 113.04952:714 130.06152:101
1.364	193.03607	Citric acid	[M+H]+	193.0343	9.17	C6H8O7	KRKNYBCHXYNGOX-UHFFFAOYSA-N	65.5	193.03607:11396 194.03942:1677 195.04278:0	43.02025:120 68.99818:368 87.01141:71

2.063	104.10895	Choline cation	[Cat]+	104.107	18.73	C5H14NO	OEYIOHPDSNJKLS-UHFFFAOYSA-N	71.4	104.10857:675177 105.11192:43736 106.11528:3074	41.03875:77 42.03456:548 43.01924:588 43.04055:742 43.08942:61 43.89357:90 44.0503:8264 44.1107:268 44.12501:132 44.27557:75 44.32546:52 44.38646:63 45.03416:7227 45.05669:7288 45.13377:144 45.15592:130 45.2523:100 45.32643:52 45.34202:69 45.53279:54 45.56498:61 46.03505:247 46.06201:367 57.77559:105 58.06623:16328 58.13291:735 58.15948:209 58.18972:178 58.25116:148 58.30614:94 58.33947:97 58.37038:124 58.41311:95 58.60676:57 58.70848:84 58.77831:52 59.07267:1092 59.95829:52 60.08197:8081 60.15258:299 60.1922:100 60.24606:106 60.44322:76 60.60894:61 61.08517:453 104.10458:73
16.182	481.40359	Bis(2,2,6,6-tetramethyl-4-piperidyl) sebacate	[M+H]+	481.39999	7.48	C28H52N2O4	XITRBUPOXXBIJN-UHFFFAOYSA-N	61.3	481.40155:2259 482.4049:1390 483.40826:0	58.06404:104 70.06476:68 140.14853:78 481.42383:60

1.62	118.08793	Betaine	[M+H]+	118.0863	13.80	C5H11NO2	KWIUHFFTVRNATP-UHFFFAOYSA-N	67.6	118.08504:243641 119.08839:15147 120.09175:11439	42.03408:447 43.04286:175 44.04915:100 56.04923:372 58.06586:14641 58.13231:612 58.15543:247 58.21812:86 58.28836:122 58.33663:87 58.49263:63 58.64717:53 59.07318:1869 59.13165:92 59.35784:51 60.07726:95 118.08438:118
7.61	91.0573	Benzylamine	[M+H-NH3]+	91.0542	34.05	C7H9N	WGQKYBSKWIADBV-UHFFFAOYSA-N	61.8	91.05577:840 92.05912:0 93.06248:0	50.01368:155 51.02243:229 63.0244:134 65.03983:160 91.0591:66
13.224	171.09961	Azelaic acid	[M+H-H2O]+	171.10159	11.57	C9H16O4	BDJRBEYXGGNYIS-UHFFFAOYSA-N	60.1	171.09988:91753 172.10323:11618 173.10659:2321	41.03841:138 43.01415:50 55.01488:70 55.05226:782 77.66525:55 79.05151:188 81.06769:143 106.94607:77
4.643	231.09891	Asp-Pro	[M+H]+	231.0975	6.10	C9H14N2O5	UKGGPJNBONZZCM-WDSKDSINSA-N	61.7	231.09959:26159 232.10294:3031 233.1063:1437	43.01849:91 70.03105:153 70.06531:797 70.36662:76 88.03994:79 96.03748:63 110.01502:50 116.07182:87 118.91486:76 125.07277:222
11.582	280.12955	Asn-Phe	[M+H]+	280.12921	1.21	C13H17N3O4	OMSMPWHEGLNQOD-NXEZZACHSA-N	63	280.13174:22779 281.13509:3922 282.13845:783	79.05785:56 87.05177:102 91.05677:651 103.05861:167 105.03275:50 119.08672:72 120.07878:197 120.12061:58 121.08109:64 128.07254:70 130.06567:56

										145.07002:52<br>157.06342:51<br>172.07376:71<br>175.08238:119<br>182.05661:104<br>200.06596:64
4.694	261.15579	Asn-Lys	[M+H]+	261.1557	0.34	C10H20N4O4	QJMCHPGWFZZRID-RQJHMYQMSA-N	60.3	261.15579:97560<br>262.15914:14481<br>263.1625:1999	56.04981:100 57.07305:52<br>69.03332:66 84.04272:77<br>84.08104:1246<br>85.08434:104<br>136.92874:57
11.302	272.17233	Arg-Pro	[M+H]+	272.17169	2.35	C11H21N5O3	LQJAALCCPOTJGB-YUMQZZPRSA-N	67.5	272.17233:200534<br>273.17568:33084<br>274.17904:20739	45.03172:69 55.05301:227<br>55.07425:68 60.0564:668<br>60.09535:70 69.06297:71<br>70.06581:9073<br>70.13626:352<br>70.16325:169<br>70.18102:129 70.20921:98<br>70.26326:74 70.32092:85<br>70.41151:82 71.06641:446<br>72.08244:681 72.15953:64<br>86.09116:142<br>97.07266:113<br>98.06068:285<br>99.06406:111<br>111.53584:75<br>112.08707:1092<br>115.0916:60<br>116.06892:792<br>123.05586:83 125.0662:95<br>126.08087:71<br>128.06807:103<br>130.09401:86<br>136.92555:199<br>140.94473:52<br>151.11234:90<br>158.09207:250<br>168.09163:86<br>175.1185:280 192.1124:92<br>195.10907:56

8.605	304.16095	Arg-Glu	[M+H]+	304.1615	1.81	C11H21N5O5	HFKJBCPRWWGPEY-RQJHMYQMSA-N	60.6	304.16095:100056 305.1643:16666 306.16766:2327	44.05063:129 60.0561:598 68.0497:53 70.06538:2215 70.15142:66 71.00961:131 71.0668:147 72.07726:85 84.03577:53 94.06208:59 97.07014:59 99.06023:53 112.08518:144 116.07175:127 117.04389:65 138.04837:57 141.07474:52 158.09305:123 175.11496:145 178.03564:61 182.07626:62 234.10083:52 240.86743:58 244.12387:55
2.073	191.10474	Ala-Thr	[M+H]+	191.1026	11.20	C7H14N2O4	BUQICHWNXBIBOG-VAYJURFESA-N	67.3	191.10451:13561 192.10786:1877 193.11122:0	44.04937:622 56.05119:287 56.07516:51 74.05914:76
5.778	187.10756	Ala-Pro	[M+H]+	187.1077	0.75	C8H14N2O3	WPWUFUBLGADILS-WDSKDSINSA-N	66.9	187.10953:113832 188.11288:11037 189.11624:0	44.05093:1725 44.10947:62 55.05431:94 56.055:198 58.06825:57 60.07833:121 68.04849:68 70.06638:2849 70.13994:81 70.23483:62 71.06635:186 72.08002:75 84.08292:154 116.0675:198
7.313	218.15147	Ala-Lys	[M+H]+	218.1499	7.20	C9H19N3O3	QXRNAOYBCYVZCD-BQBZGAKWSA-N	63.3	218.15147:186773 219.15482:27786 220.15818:3664	42.03341:120 55.05449:321 56.05035:792 57.04293:227 67.05331:175 69.06493:97 72.08266:357 74.05797:150 79.06012:52 84.08139:7223

										84.16237:246 84.2318:112 84.30962:64 84.46939:60 85.02954:1900 85.08254:858 85.15986:70 99.0508:118 102.93987:72 120.06277:86 130.08479:81
7.024	227.11591	Ala-His	[M+H]+	227.11391	8.81	C9H14N4O3	XZWXFWBHYRFLEF-CAHLUQPWSA-N	61.5	227.11563:44454 228.11898:6108 229.12234:0	44.04951:191 55.04314:104 58.06495:52 59.07487:53 68.04645:67 70.06631:155 82.06165:61 83.05821:133 84.08002:87 93.04707:167 95.05631:130 96.07832:72 109.07886:118 110.07196:828 114.0531:104 136.08746:66 142.08775:138
2.525	219.09863	Ala-Glu	[M+H]+	219.0975	5.16	C8H14N2O5	VYZAGTDAHUIRQA-WHFBIAKZSA-N	63.7	219.09981:28422 220.10316:4219 221.10652:654	41.03897:181 44.05091:636 44.07492:100 44.1087:51 45.05235:152 45.07287:66 56.05095:470 72.05496:100 84.04496:2343 84.12881:72 84.1519:52 85.04359:198 90.05982:73 102.04807:66
18.501	147.06157	Adipic acid	[M+H]+	147.0652	24.68	C6H10O4	WNLRTRBMVRJNCN-UHFFFAOYSA-N	61	147.06694:11663 148.07029:1182 149.07365:0	43.01498:105 55.05598:376 55.0905:56 58.99448:106
3.003	464.07748	Adenylosuccinic acid	[M+H]+	464.0813	8.23	C14H18N5O11P	OFBHPPMPBOJXRT-VWJPMABRSA-N	69.2	464.07748:60017 465.08083:12947 466.08419:2598	97.02688:211 136.06487:335 162.07532:451 188.0666:132 192.05032:297 192.10524:56 193.05165:167

										206.06509:216<br>218.91699:56<br>234.06366:345<br>252.07091:2006<br>252.2565:59<br>253.07385:270
2.533	348.07538	Adenosine 3'-monophosphate	[M+H]+	348.0704	14.31	C10H14N5O7P	LNQVTSROQXJCDD-NYDYUERISA-N	64.3	348.07416:100809<br>349.07751:13068<br>350.08087:2659	69.03664:135<br>85.02943:825<br>97.03049:255<br>98.98358:610<br>99.03161:121<br>109.04891:74<br>115.03648:50<br>115.95996:85<br>119.03253:63<br>136.06207:3776<br>136.16603:119<br>136.61684:51<br>136.93082:62<br>136.99942:72<br>137.06345:451<br>139.00551:70<br>152.05516:87<br>164.99751:52<br>186.07503:89
5.561	268.1055	Adenosine	[M+H]+	268.104	5.59	C10H13N5O4	OIRDTQYFTABQOQ-CRKDRTNXSA-N	67.6	268.10385:1128934<br>269.1072:170948<br>270.11056:21594	40.01914:71 41.03971:328<br>43.01797:638<br>45.03421:467<br>47.01379:104 49.02925:63<br>53.02323:60<br>55.01784:1670<br>55.08091:74 56.05357:64<br>57.03394:3068<br>57.10083:153 57.12204:53<br>59.04865:220<br>61.03109:437<br>65.01314:353<br>67.02551:119<br>69.03455:668 69.4093:53

70.02871:55 71.01457:464
71.0676:63 73.0299:937
73.10262:59 85.0291:1004
86.03385:166 87.04498:75
92.02129:293 92.04658:79
94.03895:299
97.02663:286
103.03274:67
107.04208:52
109.04893:279
110.07159:283
115.03892:108
119.03405:3660
119.12411:137
120.04074:168
135.9424:73 136.00816:65
136.06091:63383
136.162:3714
136.47987:199
136.52559:133
136.55301:145
136.64799:216
136.8029:178
136.90425:60
136.9534:103
137.06386:5271
137.16454:253
137.22629:110
137.3819:89 137.4328:62
137.49883:66
137.56224:65
137.70029:74
137.89775:59
137.99631:61
138.06587:181
148.07765:50
179.01718:56

5.557	136.0621	Adenine	[M+H]+	136.0618	2.20	C5H5N5	GFFGJBXGBJISGV-UHFFFAOYSA-N	68.9	136.06213:95131 137.06548:7146 138.06884:0	40.01939:101 41.01484:60 55.02761:244 56.96762:55 62.97966:62 65.01166:188 67.02983:375 82.04419:135 92.02402:750 94.04395:66 107.04993:59 118.91465:149 119.03286:1171 136.05606:215
1.281	162.07652	Acetylthreonine	[M+H]+	162.0761	2.59	C6H11NO4	PEDXUVCGOLSNLQ-WUJLRWPWSA-N	63.2	162.07812:31794 163.08148:9534 164.08484:827	43.01676:561 44.05098:619 45.0314:128 56.05033:1530 56.11435:87 57.03615:131 74.02152:56 74.05949:697 75.06681:84 102.04979:112
5.514	204.12708	Acetylcarnitine	[M+H]+	204.123	19.99	C9H17NO4	RDHQFKQIGNGIED-UHFFFAOYSA-N	71.8	204.12526:116163 205.12861:12933 206.13197:1803	43.01769:121 44.02044:127 57.03278:420 58.05988:67 59.07125:56 60.07976:345 61.02433:63 84.08311:241 85.02821:3829 85.13136:94 86.03353:110 87.03391:72
17.43	295.22955	9-Oxo-10(E),12(E)-octadecadienoic acid	[M+H]+	295.22681	9.28	C18H30O3	LUZSWWYKKLTDHU-SIGMCMEVSA-N	61.4	295.22983:3012 296.23318:820 297.23654:0	41.15309:68 43.05788:63 44.06127:96 53.03782:160 55.05931:135 67.05396:212 69.0704:214 71.08753:96 79.05661:91 81.03133:117 83.04665:53 91.05334:151 93.07449:84 95.04702:52 95.08822:103
5.932	298.11707	7-Methylguanosine	[M+H]+	298.11459	8.32	C11H15N5O5	OGHAROSJZRTIOK-AMKBJPJNSA-N	64.1	298.11969:10973 299.12304:2407 300.1264:833	58.96235:96 62.95489:62 124.0465:69 149.04532:202 166.06943:627 166.12836:58

4.409	150.077	7-Methyladenine	[M+H]+	150.07739	2.60	C6H7N5	HCGHYQLFMPXSDU-UHFFFAOYSA-N	63.7	150.07732:3201 151.08067:0 152.08403:532	133.04385:97
10.065	298.10025	5'Methyl-5'-thioadenosine	[M+H]+	298.0968	11.57	C11H15N5O3S	WUUGFSXJNOTRMR-IOSLPCCCSA-N	64.5	298.10028:121256 299.10363:20922 300.10699:6867	44.0515:69 46.99401:106 61.01123:1572 61.04769:137 61.06786:112 61.10787:52 62.01046:164 63.00776:138 69.03362:65 70.06389:374 71.00594:69 71.0201:55 75.02684:1113 92.02305:52 97.02999:91 117.0324:59 119.03566:164 120.03778:84 130.08536:54 136.06262:6249 136.16341:174 137.06375:334 137.12643:51 145.02806:110 200.92258:56
8.656	123.0916	4-Dimethylamino pyridine	[M+H]+	123.0917	0.81	C7H10N2	VHYFNPMBLIVWCW-UHFFFAOYSA-N	61.5	123.09377:1871 124.09712:0 125.10048:0	53.04094:61 79.04271:181 80.05378:61 107.05916:157
12.806	118.06532	4-Amino-5-(2-methylphenyl)-4H-1,2,4-triazole-3-thiol	[M+H-CH3N3S]+	118.0651	1.86	C9H10N4S	CUEYNOAJNUCBAP-UHFFFAOYSA-N	66.3	118.06532:11594 119.06867:1362 120.07203:0	51.01967:74 55.04819:78 63.02205:79 65.03869:316 89.04673:53 90.04809:112 91.05481:193 102.05051:62 117.05379:147 118.06783:89
5.973	248.15283	3-Hydroxybutyrylcarnitine	[M+H]+	248.1492	14.63	C11H21NO5	UEFRDQSMQXDWTO-YGPZHTELSA-N	61.7	248.15004:36813 249.15339:4718 250.15675:836	45.03076:62 57.03698:115 58.06511:71 59.06823:66 60.08194:89 69.03278:75 85.0285:945 86.03466:58 87.03619:57 103.03511:188

										103.07262:67 108.06875:88 152.8914:80
1.207	527.16382	3'-epi-Gemcitabine	[2M+H]+	527.15082	24.66	C9H11F2N3O4	SDUQYLNIPVEERB-NKCGXFSVSA-N	60	527.15295:828 528.1563:0 529.15966:0	142.94336:67 251.06009:60 527.15723:177
3.986	116.10984	3-Dehydrocarnitine	[M+H-CO2]+	116.107	24.46	C7H13NO3	YNOWULSFLVIUDH-UHFFFAOYSA-N	65.4	116.10958:50874 117.11293:6032 118.11629:2126	58.06673:2570 59.07335:103
2.506	129.06757	3-Aminopiperidine-2,6-dione	[M+H]+	129.0659	12.94	C5H8N2O2	NPWMTBZSRRLQNJ-UHFFFAOYSA-N	60.2	129.06668:3055 130.07003:1607 131.07339:0	56.05083:88 83.06769:65 84.04422:70
1.217	138.05528	3-Acetoxypyridine	[M+H]+	138.0549	2.75	C7H7NO2	QZDWODWEESGPLC-UHFFFAOYSA-N	63.4	138.05545:6290 139.0588:785 140.06216:0	96.04929:60
1.09	707.22095	3α-Mannobiose	[2M+Na]+	707.22162	0.95	C12H22O11	QIGJYVCQYDKYDW-VXSGSMIHSA-N	66.6	707.2193:913 708.22265:0 709.22601:0	203.0459:130 365.10922:652
9.981	181.09032	3,5-Dimethyl-4-methoxybenzoic acid	[M+H]+	181.08591	24.35	C10H12O3	WXVQURJGDUNJCS-UHFFFAOYSA-N	62.3	181.09032:1585 182.09367:0 183.09703:1312	137.1039:145
6.812	118.08758	3-(Dimethylamino) propionic acid	[M+H]+	118.0863	10.84	C5H11NO2	JMOXSQYGVIXBBZ-UHFFFAOYSA-N	64.2	118.08797:4460 119.09132:0 120.09468:0	58.06451:147
3.126	155.07971	3-(5-Methyl-1H-pyrazol-1-yl) propanoic acid	[M+H]+	155.0815	11.54	C7H10N2O2	AVWPEIKOPXXROD-UHFFFAOYSA-N	66.6	155.08028:10662 156.08363:1772 157.08699:0	55.05492:98 83.05132:60
12.81	146.06065	3-(2-Nitro-1-propenyl)indole	[M+H-C2H3NO]+	146.06	4.45	C11H10N2O2	BPAQTDYDNBMYAM-SOFGYWHQSA-N	70.4	146.06078:25774 147.06413:3315 148.06749:0	65.0404:219 90.04875:146 91.05293:1622 91.41978:62 92.0526:85 102.93526:53 117.05634:532 118.06472:272

12.807	144.08015	3-(2-Hydroxyethyl) indole	[M+H-H2O]+	144.0808	4.51	C10H11NO	MBBOMCVGYCRMEA-UHFFFAOYSA-N	62.4	144.08084:14984 145.08419:1829 146.08755:28516	65.03818:131 66.04118:103 72.07688:67 77.0368:167 84.08275:86 89.03759:172 90.0411:70 91.05473:1229 91.13766:70 92.05367:76 115.05363:204 116.06121:180 117.05844:966 118.06425:274 127.06076:79 142.05856:70 143.07124:273 143.1165:70
10.535	103.05646	2-Methylisoquinolin-2-ium cation	[Cat-C2H3N]+	103.0542	21.93	C10H10N	ZONQVNWUCWMRBX-UHFFFAOYSA-N	63.7	103.05591:9921 104.05926:1034 105.06262:0	50.01472:100 51.02369:2278 51.09241:82 52.02427:179 63.02475:125 77.03801:786 78.04012:86
12.301	187.09679	2-Methylbutyryl carnitine	[M+H-C3H9N]+	187.0965	1.55	C12H23NO4	IHCPDBBYTYJYIL-QVDQXJPCSA-N	67.3	187.09808:22684 188.10143:2358 189.10479:0	57.03033:147 57.07316:114 85.0295:500 91.05926:78
6.25	119.03529	2-Methoxy-6-nitrobenzonitrile	[M+H-CH2NO2]+	119.0366	11.01	C8H6N2O3	ZHJPRHIYTNVTLI-UHFFFAOYSA-N	62.1	119.03497:1378 120.03832:0 121.04168:0	44.04853:50 91.05361:57 119.02935:101
10.537	107.04799	2-Hydroxybenzene methanol	[M+H-H2O]+	107.0491	10.37	C7H8O2	CQRYARSYNCAZFO-UHFFFAOYSA-N	63.3	107.05102:1773 108.05437:0 109.05773:0	51.02343:407 52.02712:74 77.03863:1215 77.11738:58 78.0435:71 79.05373:78
5.813	535.20422	2'-Deoxyguanosine	[2M+H]+	535.20081	6.37	C10H13N5O4	YKBGVTZYEHREMT-KVQBGUIXSA-N	63.7	535.20422:1551 536.20757:577 537.21093:0	136.06378:252 152.05627:1238 153.05487:76 268.10867:113 286.05038:54

5.062	228.09651	2'-Deoxycytidine	[M+H]+	228.0979	6.09	C9H13N3O4	CKTSBUTUHBMZGZ-BBVRLYRLSA-N	60.7	228.09837:8010 229.10172:997 230.10508:1150	43.02327:62 45.03595:180 67.031:105 69.04657:97 70.06667:355 81.03367:63 85.03254:53 94.03848:134 95.03273:54 112.04798:831 125.06149:75
6.253	252.11116	2'-Deoxyadenosine	[M+H]+	252.1091	8.17	C10H13N5O3	OLXZPDWKRNYJJZ-RRKCRQDMSA-N	67.8	252.10999:206481 253.11334:27993 254.1167:2613	41.04036:110 42.96753:53 43.01732:158 43.05725:699 45.03328:682 57.03307:189 69.03397:207 71.01754:87 71.04967:420 73.02878:513 81.03089:114 82.03217:83 92.02378:62 94.03862:69 99.04831:151 109.05415:65 117.05644:60 119.03887:413 120.03863:63 136.06284:7638 136.16316:209 136.20763:127 136.28909:56 137.0647:961
4.158	200.04082	2-Chlorophenylalanine	[M+H]+	200.0473	32.39	C9H10ClNO2	CVZZNRXMDCOHBG-QMMMGPOBSA-N	61.9	200.0425:1900 201.04585:0 202.04921:0	154.04236:87
12.807	142.06531	2-Chloro-6-methyl-3-quinoline carbaldehyde	[M+H-CHClO]+	142.06509	1.55	C11H8ClNO	FSLNYYZJXMGKHK-UHFFFAOYSA-N	63.5	142.06601:4120 143.06936:10921 144.07272:12079	65.03683:96 89.0397:279 91.05576:171 114.04163:61 115.05614:764 116.05592:161 117.05788:233 128.04944:143

										140.04077:71 142.06306:208
2.532	162.07774	2-Aminoadipic acid	[M+H]+	162.0761	10.12	C6H11NO4	OYIFNHCXNCRBQI-UHFFFAOYSA-N	68.9	162.07774:218313 163.08109:18768 164.08445:3095	43.01956:166 43.05523:105 44.0512:110 55.01938:2267 55.11752:65 55.13005:53 56.05175:1178 56.11504:65 57.03223:87 57.04977:64 60.0825:174 70.06558:991 71.06922:228 73.02708:118 98.06738:283 99.03069:52 102.09259:57
10.536	120.08222	2-Amino-1-phenylethanol	[M+H-H2O]+	120.0808	11.83	C8H11NO	ULSIYEODSMZIPX-UHFFFAOYSA-N	71.1	120.08195:660092 121.0853:61484 122.08866:2888	42.03566:240 43.04646:68 51.02472:3061 51.07156:184 51.08597:112 52.02799:278 53.03861:80 63.02896:67 65.03995:1291 65.12964:68 66.04427:263 75.02605:92 77.04025:12215 77.11751:422 77.13998:178 77.16566:153 77.22078:95 77.26369:86 77.34902:70 77.43271:78 77.50092:56 77.52402:72 77.63527:64 78.043:1178 78.10303:75 78.27956:96 79.04181:97 80.04783:71 91.05527:2753 91.14136:111 92.05933:335 101.03669:55 102.04889:420 103.05636:1813

										103.12616:105 103.18754:59 103.52067:72 104.05386:226 118.06842:238 119.0735:208 120.07751:64
16.177	306.24362	2,6-Di-tert-butyl-4-(4-morpholinylmethyl) phenol	[M+H]+	306.2428	2.68	C19H31NO2	KWGUJRCPGGSTKB-UHFFFAOYSA-N	61.6	306.24551:2779 307.24886:1457 308.25222:520	160.08385:126 174.19106:60 178.13605:62 219.17418:430 306.23523:63
5.088	124.08698	2,5-Dimethyl-4-pyrimidinamine	[M+H]+	124.0869	0.64	C6H9N3	UXKNAXNFIYFMIB-UHFFFAOYSA-N	64.3	124.08698:10621 125.09033:1239 126.09369:628	56.04979:185
5.933	166.07454	2,3,4,5-Tetrahydro-1,4-benzothiazepine	[M+H]+	166.0685	36.37	C9H11NS	SUBDEKBXSIKCSA-UHFFFAOYSA-N	61.7	166.0731:3290 167.07645:546 168.07981:0	166.07158:65
7.677	114.10464	1-Pyrrolidine carboximidamide	[M+H]+	114.1026	17.88	C5H11N3	PIGIRWPZTWLVLB-UHFFFAOYSA-N	63.3	114.10526:4405 115.10861:0 116.11197:0	55.03326:111 72.08109:78
6.143	126.06586	1-Methylcytosine	[M+H]+	126.0662	2.70	C5H7N3O	HWPZZUQOWRWFDB-UHFFFAOYSA-N	61.4	126.0648:5048 127.06815:829 128.07151:0	109.07303:85
4.414	282.12064	1-Methyladenosine	[M+H]+	282.11969	3.37	C11H15N5O4	GFYLSDSUCHVORB-FCKMSMMTSA-N	60.3	282.11935:29791 283.1227:4366 284.12606:1221	58.96109:95 94.03983:68 133.05186:74 150.0768:1769 150.19096:50
2.265	130.08502	1-Aminocyclopentanecarboxylic acid	[M+H]+	130.0863	9.84	C6H11NO2	NILQLFBWTXNUOE-UHFFFAOYSA-N	63.3	130.08286:6150 131.08621:0 132.08957:1169	84.08155:162

6.829	153.14049	1,8-Diazabicyclo[5.4.0]undec-7-ene	[M+H]+	153.1386	12.34	C9H16N2	GQHTUMJGOHRCHB-UHFFFAOYSA-N	65.1	153.14049:60211<br>154.14384:6145<br>155.1472:0	42.03424:126<br>44.05118:404 55.0559:372<br>68.04631:124 69.0724:226<br>96.0815:821 96.12801:84<br>97.08769:131<br>98.08232:227<br>109.07035:74<br>111.0938:248<br>124.09325:63<br>125.1088:476<br>125.35406:72<br>153.14088:1249<br>153.21458:98
18.57	279.15826	1,4-Dibutyl benzene-1,4-dicarboxylate	[M+H]+	279.15909	2.97	C16H22O4	LQLQDKBJAIILIQ-UHFFFAOYSA-N	62.7	279.15811:55236<br>280.16146:13723<br>281.16482:1557	43.05769:75 57.07237:104<br>65.04001:95<br>121.02625:157<br>149.01993:744<br>150.02637:109
12.683	72.08189	1,2-Diamino-2-methylpropane	[M+H-NH3]+	72.0808	15.12	C4H12N2	OPCJOXGBLDJWRM-UHFFFAOYSA-N	62.1	72.08189:14490<br>73.08524:793<br>74.0886:3386	42.03323:158<br>55.05378:127<br>56.04852:163 57.05719:86
12.809	143.07385	1-(4-Methoxyphenyl)-1H-pyrrole	[M+H-CH3O]+	143.073	5.94	C11H11NO	WYFMHHMFUMBCGI-UHFFFAOYSA-N	61.2	143.07452:9092<br>144.07787:11398<br>145.08123:1006	65.03796:254 89.03989:69<br>90.04705:176<br>91.05525:510 91.11145:54<br>101.04967:77<br>115.05867:654<br>116.05891:125<br>117.06125:395<br>118.06359:156<br>142.07558:83<br>143.07593:277
1.247	335.06573	β-Nicotinamide mononucleotide	[M+H]+	335.0639	5.46	C11H15N2O8P	DAYLJWODMCOQEW-TURQNECASA-N	67.5	335.06332:17608<br>336.06667:2171<br>337.07003:627	53.03657:62 80.04749:53<br>97.02915:454<br>98.03485:106<br>106.02593:59<br>123.0554:1022

7.408	148.06039	α-Guanidino glutaric acid	[M+H-CH2N2]+	148.06039	0.00	C6H11N3O4	RHVVRMJOHATSPD-VKHMYHEASA-N	66.2	148.06284:1378 149.06619:0 150.06955:0	56.05594:107 84.04668:96 84.07423:77
9.833	232.15805	Butyrylcarnitine	[M+H]+	232.1543	16.15	C11H21NO4	QWYFHHGCZUCMBN-SECBINFHSA-N	67.3	232.15556:645202 233.15891:87798 234.16227:9953	41.03886:88 43.01789:138 43.05596:1701 43.11219:74 44.0595:54 44.99747:229 57.03373:2134 58.03433:139 58.06579:107 58.10399:66 59.07047:315 60.08087:1675 60.12778:91 60.15114:97 71.05275:288 84.08148:412 85.02922:24172 85.10889:948 85.14163:358 85.20018:244 85.27773:124 85.34407:112 85.40762:85 85.49803:88 85.59771:79 85.64507:60 85.66129:52 85.7021:101 86.03175:1216 86.09203:101 86.14253:73 86.19368:60 86.44883:51 87.03429:142 89.05507:142 98.0927:71 102.08495:75 118.92081:184 129.07545:118 136.93018:160 144.09734:289 155.02859:73

7.613	119.05521	(4-Bromo-1H-pyrazol-1-yl)(3-methylphenyl) methanone	[M+H-C3H3BrN2]+	119.0491	51.32	C11H9BrN2O	WACJMZOPBBWBAJ-UHFFFAOYSA-N	65.5	119.05193:10313 120.05528:1008 121.05864:1240	65.03879:123 91.05412:263 94.05945:65
7.677	131.13237	(4-Aminobutyl) guanidine	[M+H]+	131.1291	24.94	C5H14N4	QYPPJABKJHAVHS-UHFFFAOYSA-N	68	131.13141:36984 132.13476:3160 133.13812:0	43.02907:81 55.05174:177 60.05598:130 72.08193:490
2.923	146.11781	(3-Carboxypropyl) trimethylammonium cation	[Cat]+	146.1176	1.44	C7H16NO2	JHPNVNIEXXLNTR-UHFFFAOYSA-O	69.1	146.11781:51246 147.12116:4635 148.12452:699	41.03719:140 43.01712:391 44.04644:75 45.03236:954 58.06423:62 60.08135:209
7.182	262.16794	3-Hydroxyisovaleroyl carnitine	[M+H]+	262.16489	11.63	C12H23NO5	IGLHHSKNBDXCEY-SECBINFHSA-N	66.1	262.1676:94229 263.17095:15200 264.17431:2175	57.0364:80 59.04953:574 60.04723:71 60.08307:124 85.02923:2374 86.03703:85

Table 2S. LC/MS Q-TOF raw data file**:** area values obtained from the analysis of twelve *P. ostreatus* samples, six grown on WS substrate (WS 1-6) and six on the LcS substrate (LcS 1-6).

**Sample**	**WS 1**	**WS 2**	**WS 3**	**WS 4**	**WS 5**	**WS 6**	**LcS 1**	**LcS 2**	**LcS 3**	**LcS 4**	**LcS 5**	**LcS 6**
3-Hydroxyisovaleroylcarnitine	720889	619195	554704	806898	716103	666282	78008	70289	612878	500149	483471	433273
(3-Carboxypropyl)trimethylammonium cation	141897	375238	447296	153594	191995	239755	0.0053	0.0041	0.0034	0.0016	0.0052	0.0047
(4-Aminobutyl)guanidine	0	0	0	191597	208628	21704	278703	44954	482583	3403	683332	57161
(4-Bromo-1H-pyrazol-1-yl)(3-methylphenyl)methanone	0.0073	0.0087	0.0083	0.0082	0.0079	0.0079	0.0089	0.0094	102102	0.0081	0.0094	0.0094
Butyrylcarnitine	1E+07	1E+06	1E+07	4E+06	439899	4E+06	2E+07	2E+07	2E+07	1E+07	1E+07	1E+07
α-Guanidinoglutaric acid	0.0013	0.0012	0.0012	0.0008	0.0009	0.0008	0.0012	0.0011	0.001	0.0012	0.0011	0.0012
β-Nicotinamide mononucleotide	0.0083	0.0041	0.003	148683	0.0095	0.0024	15602	142617	13831	0.0099	0.0093	108851
1-(4-Methoxyphenyl)-1H-pyrrole	0.0074	0.009	0.0095	0.0078	0.0083	0.0083	101111	106227	107783	0.0095	1038	101026
1,2-Diamino-2-methylpropane	225262	2E+06	2E+06	2E+06	2E+06	2E+06	3E+06	3E+06	3E+06	3E+06	27498	3E+06
1,4-Dibutyl benzene-1,4-dicarboxylate	484346	467875	258227	710206	302028	310376	590551	276575	266668	656785	277908	296264
1,8-Diazabicyclo[5.4.0]undec-7-ene	6E+06	4E+06	3E+06	4E+06	372124	4E+06	4E+06	3E+06	3E+06	3696	3E+06	319894
1-Aminocyclopentanecarboxylic acid	0.0005	0.0001	0.0001	0.0016	0.0016	0.0015	0.0025	0.0024	0.0023	0.0064	0.0054	0.0051
1-Methyladenosine	227912	252734	258458	159916	147408	156685	601333	54235	537014	3429	338538	37022
1-Methylcytosine	0.0056	0.0057	0.0053	0.0034	0.0029	0.0031	0.0042	0.0041	0.0034	0.0049	0.0044	0.0053
1-Pyrrolidinecarboximidamide	0.0003	0.0001	0.0003	0.0012	0.0016	0.0018	0.0019	0.003	0.0036	0.0025	0.0049	0.004
2,3,4,5-Tetrahydro-1,4-benzothiazepine	0.0036	0.0045	0.0046	0.0028	0.003	0.0036	0.0068	0.008	0.0085	0.0052	0.0059	0.0069
2,5-Dimethyl-4-pyrimidinamine	0.0012	0.0011	0.0011	0.0014	0.0013	0.0012	0.0025	0.0023	0.0021	0.0081	0.0074	0.0063
2,6-Di-tert-butyl-4-(4-morpholinylmethyl)phenol	0.0028	0.0029	0.0028	0.0032	0.003	0.0029	0.0021	0.0025	0.0023	0.0022	0.0026	0.0028
2-Amino-1-phenylethanol	4E+06	44751	5E+06	4E+06	425238	4E+06	5E+06	5E+06	5E+06	5E+06	526243	5E+06
2-Aminoadipic acid	248136	108877	0.0001	152033	101886	235944	2E+06	2E+06	1E+06	1E+06	1E+06	1E+06
2-Chloro-6-methyl-3-quinolinecarbaldehyde	0.0034	0.0036	0.0042	0.0034	0.0037	0.0038	0.0042	0.0046	0.0047	0.004	0.0048	0.0045
2-Chlorophenylalanine	0.0006	0.0003	0.0003	0.0005	0.0007	0.0005	0.0021	0.0017	0.0017	0.0014	0.0018	0.0016

2'-Deoxyadenosine	2E+06	2E+06	171637	129731	1E+06	1E+06	2E+06	2E+06	2E+06	1E+06	2E+06	2E+06
2'-Deoxycytidine	100469	0.0081	0.0097	0.0063	0.0057	0.0056	0.0032	0.0035	0.0039	0.0085	0.0096	0.0083
2'-Deoxyguanosine	0.0006	0.0006	0.0006	0.0001	0	0	0.0012	0.0009	0.0009	0.0006	0.0009	0.0007
2-Hydroxybenzenemethanol	0.0017	0.0018	0.0017	0.0015	0.0015	0.0018	0.0022	0.0021	0.002	0.0021	0.0021	0.002
2-Methoxy-6-nitrobenzonitrile	0.0011	0.0009	0.001	0.0008	0.0008	0.0007	0.0011	0.0013	0.0011	0.0009	0.0011	0.0011
2-Methylbutyrylcarnitine	175505	19142	197997	0.0047	0.0047	0.0049	153529	170119	166223	100699	117227	114028
2-Methylisoquinolin-2-ium cation	0.0074	0.008	0.0087	0.0074	0.0075	0.0072	0.0093	0.0097	0.0094	0.0092	0.0098	0.0093
3-(2-Hydroxyethyl)indole	0.0094	107182	11114	0.0092	0.01	0.0099	120803	128373	125092	117421	127655	125236
3-(2-Nitro-1-propenyl)indole	171943	189699	193529	171536	18084	174767	205894	228282	216416	201023	219851	215416
3-(5-Methyl-1H-pyrazol-1-yl)propanoic acid	0.0082	0.0081	0.0077	113414	107604	10175	150489	150486	160054	0.0099	103423	102271
3-(Dimethylamino)propionic acid	0.003	0.0031	0.0034	0.003	0.0035	0.0034	0.0034	0.0031	0.0032	0.0031	0.0035	0.0033
3,5-Dimethyl-4-methoxybenzoic acid	0	0	0	0.0003	0.0002	0.0003	0	0	0	0.0012	0.001	0.0013
3α-Mannobiose	0.0011	0.0003	0.0002	0.0001	0.0001	0.0001	0.0005	0.0007	0.0004	0.0004	0.0003	0.0001
3-Acetoxypyridine	0.0064	0.0061	0.0055	0.0023	0.0023	0.0018	0.0036	0.0041	0.004	0.0052	0.0049	0.0065
3-Aminopiperidine-2,6-dione	0.0013	0.0016	0.0014	0.0027	0.0025	0.0028	0.0024	0.0025	0.0024	0.0062	0.0066	0.0068
3-Dehydrocarnitine	267983	207442	193859	117837	119367	148255	1E+06	1E+06	10456	129638	121011	1E+06
3'-epi-Gemcitabine	1E+06	1E+06	1E+06	1E+06	1E+06	1E+06	461587	336933	237192	954085	628355	617801
3-Hydroxybutyrylcarnitine	1E+06	1E+06	1E+06	2E+06	2E+06	183401	613169	584045	50633	131321	1E+06	1E+06
4-Amino-5-(2-methylphenyl)-4H-1,2,4-triazole-3-thiol	0.0066	0.0081	0.0079	0.0066	0.0072	0.0075	0.0087	0.0094	0.0089	0.008	0.0087	0.0092
4-Dimethylaminopyridine	0.0019	0.002	0.0015	0.0021	0.0016	0.0016	0.0017	0.0017	0.0014	0.0014	0.0015	0.0015
5'-Methyl-5'-thioadenosine	102462	1E+06	1E+06	224828	223693	194815	726774	746603	626232	172706	172923	149879
7-Methyladenine	0.0025	0.0025	0.0027	0.0017	0.0017	0.0015	0.0064	0.006	0.0056	0.0043	0.0041	0.0044
7-Methylguanosine	0.0084	0.01	10311	0.0056	0.0066	0.0072	16472	189884	200602	115938	122341	154391
9-Oxo-10(E),12(E)-octadecadienoic acid	0.0007	0.0016	0.0015	0.0024	0.0025	0.0028	0.0024	0.0026	0.0027	0.0031	0.0036	0.0035
Acetylcarnitine	9E+06	8E+06	8E+06	1E+07	1E+07	1E+07	6E+06	6E+06	6E+06	502437	494142	5E+06
Acetylthreonine	0.0039	0.0029	0.0028	0.0021	0.002	0.0024	230008	265334	259869	165642	155699	154671
Adenine	727792	727553	701218	651649	591415	605258	737909	759391	721212	710003	747236	755755
Adenosine	1E+07	1E+07	1E+07	9E+06	8E+06	8E+06	1E+07	1E+07	1E+07	1E+07	1E+07	1E+07
Adenosine 3'-monophosphate	1E+06	109841	879908	1E+06	1E+06	1E+06	505853	417891	327047	774629	682876	583984
Adenylosuccinic acid	166541	139744	139979	448216	4214	551091	229997	216827	166362	171	176681	178502
Adipic acid	150929	15052	0.0098	210834	102766	104825	181568	0.0096	0.0089	20292	0.0099	101919
Ala-Glu	308378	294224	248079	209458	216481	248304	341883	30314	288342	215716	203813	238321
Ala-His	344295	376545	38156	41497	406572	391892	213413	21728	22052	226673	24983	198756

Ala-Lys	2E+06	2E+06	4E+06	404072	408042	4E+06	2E+06	2E+06	3E+06	2E+06	2E+06	181072
Ala-Pro	1E+06	992316	993574	819749	836659	863446	133852	100028	0.0089	0.0086	0.0078	0.007
Ala-Thr	179187	112109	118365	0.0093	0.0096	0.01	0.0058	0.0044	0.0048	0.0045	0.0057	0.0044
Arg-Glu	765467	758634	691261	653957	62376	556807	510812	360137	26061	615933	507989	372845
Arg-Pro	2E+06	2E+06	2E+06	135611	1E+06	1E+06	0.0097	0.0072	0.0062	0.0089	0.0091	0.0052
Asn-Lys	265563	212037	175417	0.0093	0.0068	0.0054	639507	581626	530777	623369	771488	556757
Asn-Phe	200441	198342	198824	198638	208862	18469	0.0033	0.0023	0.0018	0.0068	0.0047	0.0031
Asp-Pro	229771	221577	238767	175092	187355	219397	176773	15986	160421	104898	124056	111972
Azelaic acid	224723	302901	718082	764828	836001	8425	0.0037	0.0065	0.0074	0.0063	0.0077	0.009
Benzylamine	0.0008	0.0009	0.0008	0.0008	0.0007	0.0008	0.001	0.0011	0.0009	0.001	0.0009	0.001
Betaine	198934	151935	127522	395801	313168	543076	7E+06	8E+06	7E+06	6E+06	8E+06	7E+06
Bis(2,2,6,6-tetramethyl-4-piperidyl) sebacate	0.0016	0.0022	0.0035	0.001	0.0022	0.0015	0.0017	0.0016	0.0012	0.0007	0.001	0.001
Choline cation	4E+06	6E+06	6E+06	6E+06	6E+06	5E+06	1E+07	1E+06	1E+07	1E+07	1E+07	1E+07
Citric acid	0.0043	0.003	0.002	0.003	0.0022	0.0021	0.0023	0.0029	0.0024	0.0079	0.009	0.0065
Cytarabine	2E+06	1E+06	1E+06	224958	2E+06	2E+06	530761	351485	229851	2E+06	1E+06	105583
Cytidine 2',3'-cyclic monophosphoric acid	0.0068	0.0032	0.0014	0.0013	0.0002	0.0001	0.0017	0.0014	0.0011	0.0057	0.0032	0.0009
Cytidine 3'-monophosphate	69366	542284	240494	443731	403706	438333	128543	125748	131257	219555	191331	224669
Decanoylcarnitine	0.0008	0.0007	0.001	0.0014	0.0014	0.0014	0.0005	0.0006	0.0005	0.0007	0.0006	0.0007
D-Glucose 6-phosphate	179669	0.0071	0.0063	0.0074	0.0062	0.0071	0.0062	0.0068	0.0061	0.0067	0.0053	0.0051
Di(5-nonyl) phthalate	2E+06	2E+06	93156	235978	1E+06	1E+06	2E+06	985004	939274	2E+06	964758	1E+06
Dibutyl adipate	398977	40493	254029	557844	278277	286868	491859	251091	253074	546325	258093	271768
Di-n-octyl phthalate	9E+06	8E+06	5E+06	1E+06	6E+06	6E+06	102368	5E+06	5E+06	1E+06	5E+06	543462
Norvaline	0.0086	0.0085	0.009	0.0082	0.0084	0.0086	104966	109525	11839	103509	109833	114524
Phenylalanine	1E+07	1E+07	1E+07	1E+07	1E+07	1E+07	1E+07	1E+07	1E+07	1E+07	1E+07	1E+07
Mannitol	158861	0.0085	0.0065	110067	127269	140509	0.005	0.0043	0.0046	0.0088	0.0062	113309
Ornithine	569643	506572	484816	713992	715488	742235	3E+06	318566	307904	909256	904047	86555
D-Pipecolinic acid	662888	643453	661003	665523	665173	655857	120178	1E+06	1E+06	1E+06	1E+06	1E+06
Ergothioneine	918491	679556	719043	802981	757997	943438	1E+06	2E+06	2E+06	732591	823195	1E+06
Ethanolamine	0.0035	0.0058	0.0034	0.0066	0.0055	0.0052	0.0016	0.0019	0.0021	0.002	0.0022	0.0021
Ethylmalonic acid	0.004	0.0035	0.0034	0.0028	0.0029	0.003	0.0071	0.004	0.0033	0.0039	0.0039	0.0034
Gln-Ala	315851	286731	260582	216395	213649	215718	189164	15016	106822	163891	139565	100602
Gln-Asn	178745	149642	150591	152351	144104	155731	131319	122874	109845	104841	0.0092	0.0094
Gln-Asp	0.0071	148704	178714	0.0076	101844	109302	0.0083	169713	194442	0.009	143581	169326

Gln-Gln	395987	660842	68784	431726	477559	432828	310283	331839	27755	347955	327975	301519
Gln-Gly	16726	223923	223177	214761	219445	226792	100473	112752	108148	11104	104864	106131
Gln-Ile	251296	239991	288831	167521	213114	195559	170906	146311	118308	281724	240713	200328
Gln-Leu	85482	773111	861754	451264	553058	503443	0.0094	0.0072	0.0059	178591	0.0099	0.0073
Gln-Met	193311	157442	151113	0.0083	0.0081	0.0063	0.0038	0.0026	0.0015	0.0063	0.0035	0.0023
Gln-Pro	681132	565429	561687	496195	484169	400289	242259	0.0007	0.0004	0.0003	0.0008	0.0063
Gln-Ser	766138	671229	65266	398835	429975	437546	434339	388431	383161	246785	258003	259449
Gln-Thr	204625	121126	143784	0.0091	0.0087	130427	100509	0.0094	192211	0.0087	105137	206175
Gln-Tyr	190467	163227	16168	146326	138786	122086	0.003	0.0017	0.0019	0.005	0.003	0.0023
Gln-Val	354717	291936	293664	272664	261773	223618	326841	329991	273747	346537	323991	277455
Glu-Gln	153894	0.0091	0.0099	0.0068	0.0074	0.0095	0.0096	0.007	0.0098	0.0062	0.0063	0.0095
Glu-Gly	583103	527987	511422	438912	413644	401001	591795	577028	512029	470679	443891	416777
Glu-His	192457	224225	203299	180509	181807	192206	354017	315299	302363	276514	301275	314032
Glu-Ile	428102	435387	473264	259054	343969	323728	667047	613348	624996	63187	652818	704912
Glu-Pro	110942	125194	111067	0.0093	0.0096	0.0096	0.0092	0.0082	0.0081	0.0064	0.0065	0.0056
Glu-Thr	216575	177493	136912	115966	103273	0.0091	226264	171525	136118	149858	129637	0.01
Glu-Trp	19201	224823	304717	114996	143509	146575	402819	425418	469853	316696	375885	433352
Glu-Tyr	128257	128502	148042	0.0095	115453	118088	175213	16798	148574	164195	147943	152257
Glu-Val	386974	330773	346968	238317	249041	224774	41743	36541	33285	409805	388175	360826
Gly-Arg	639548	646709	686023	454791	478751	470636	544518	485573	481636	456921	497653	457575
Glycerophosphocholine	3E+06	1E+06	976554	748372	587345	77536	108698	109281	0.0061	398955	21711	133164
Gly-Val	383026	34204	322764	242613	252062	230406	667968	55173	554114	567615	578345	584661
Granisetron	0.0032	0.0034	0.0037	0.0001	0.0001	0.0002	0.0005	0.0004	0.0005	0.0002	0.0001	0.0001
Guanidine	0.0037	0.0041	0.0035	0.0034	0.0036	0.003	0.005	0.0052	0.0048	0.0056	0.0054	0.0058
Guanine	2E+06	2E+06	2E+06	2E+06	2E+06	2E+06	2E+06	2E+06	2E+06	22904	2E+06	2E+06
Guanosine	302647	3E+06	3E+06	3E+06	2E+06	2E+06	4E+06	4E+06	4E+06	4E+06	4E+06	4E+06
Guanosine 5'-monophosphate	467264	42994	339673	458157	394254	429205	128187	106829	0.0079	389634	315644	276806
Guanosine 5'-tetraphosphate	0.0054	0.0028	0.0027	0.0019	0.0018	0.0015	113337	0.007	0.0053	109116	0.0085	0.0048
Hexanoylcarnitine	0.0054	0.0089	0.006	0.0037	0.0041	0.0043	0.0095	0.0095	122261	0.0082	0.0095	0.0097
His-Ala	271484	243961	200723	558942	498339	419557	371532	388797	349278	48909	490785	461261
His-Asn	218098	19538	179047	211921	0.0082	180707	223585	218914	20221	215246	216629	193222
His-Asp	128768	126865	125921	149147	158309	175421	148015	147336	136972	13605	131261	132554
His-Glu	305955	295965	2502	321657	27004	241466	159596	115121	0.0083	207631	163867	13313

His-Gly	156223	29701	306943	316569	302112	330504	248954	209503	173977	324637	316259	270639
His-His	0.0052	0.0044	0.0035	0.007	0.0067	0.0059	133005	114142	107653	162265	173286	156426
His-Ile	0.0015	0.0017	0.0006	0.0045	0.0027	0.0028	531295	493863	439171	679918	700643	615804
His-Lys	0.0043	0.0033	0.0024	0.0063	0.0046	0.0032	197795	170813	142179	188124	201268	14957
His-Pro	734314	736198	767975	673139	776384	653452	101613	0.0082	0.0067	0.0088	0.0091	0.005
His-Trp	0.0004	0.0003	0	0	0	0	0.009	0.0072	0.0057	106592	0.0086	0.0069
His-Val	0.0015	0.0017	0.0012	0.0067	0.0041	155421	742848	694074	62057	902315	939502	797439
Ile-Ala	153992	2E+06	2E+06	1E+06	1E+06	1E+06	2E+06	2E+06	2E+06	2E+06	2E+06	1E+06
Ile-Gln	520663	40747	347159	41154	372422	290446	197219	181931	137222	322827	236609	155423
Ile-Glu	1E+06	971129	1E+06	713478	794446	723115	1E+06	1E+06	919986	1E+06	1E+06	970735
Ile-Gly	863591	912404	98605	854838	900473	917236	1E+06	1E+06	1E+06	125681	1E+06	1E+06
Ile-His	265972	257586	226735	312912	313755	285189	550285	523347	482748	50526	557787	500714
Ile-Leu	3E+06	3E+06	2E+06	2E+06	2E+06	2E+06	2E+06	2E+06	2E+06	3E+06	3E+06	2E+06
Ile-Lys	1E+06	1E+06	1E+06	1E+06	1E+06	1E+06	2E+06	1E+06	1E+06	2E+06	2E+06	143271
Ile-Pro	4E+06	4E+06	5E+06	4E+06	4E+06	4E+06	4E+06	4E+06	4E+06	3E+06	4E+06	3E+06
Ile-Ser	48522	42181	415333	26309	249315	222313	770071	69253	705253	676988	680854	599213
Ile-Thr	639222	550736	547042	39189	424727	369083	438443	414592	360424	452157	438183	319582
Ile-Val	928594	884677	873596	731695	826871	735117	3E+06	4E+06	3E+06	3E+06	4E+06	3E+06
Indapamide	0.0071	0.0083	0.0084	0.0069	0.0078	0.0076	0.0089	0.0093	0.0094	0.0084	0.0094	0.0095
Isovalerylcarnitine	2E+07	2E+07	2E+07	6E+06	6E+06	7E+06	2E+07	2E+07	2E+07	1E+07	2E+07	2E+07
β-Homolysine	0.0036	0.0006	0.001	0.0043	0.0011	0.0088	0.0037	0.0041	0.0052	364675	389203	435148
γ-Glutamylglutamic acid	394942	391178	373017	3045	318118	338234	413624	354179	30747	310691	289659	285984
Alanylnorleucine	1E+06	1E+06	1E+06	945313	1E+06	1E+06	738431	819641	749313	948361	1E+06	690505
Arginine	1E+07	1E+07	9E+06	9E+06	9E+06	9E+06	1E+07	1E+06	1E+07	1E+06	1E+07	1E+07
Arginine, methyl ester	0.0035	0.0042	0.0037	0.0028	0.0029	0.003	0.0084	0.0089	0.0077	0.0069	0.0042	0.0047
Aspartic acid	180348	134722	138245	0.0076	0.007	0.0081	0.0039	0.0038	0.0039	0.0014	0.0016	0.0018
Carnitine	3E+06	3E+06	4E+06	2E+06	27997	4E+06	1E+06	1E+06	2E+06	1E+06	1E+06	2E+06
Cystathionine	769052	758579	689456	449824	355434	42931	460598	484563	414429	754862	420224	817645
Leu-Arg	526349	349508	261289	644513	575231	459636	369247	339129	244292	595909	575884	276295
Leu-Gly	289187	307752	383527	394264	453146	47215	318254	334445	406728	336331	434825	437751
Leu-His	0.0019	0.0018	0.0011	0.0047	0.0032	0.0021	153095	109767	0.0078	279537	191466	131405
Leu-Leu	0.0087	0.0053	0.0043	0.0001	0.0036	0.002	0.0066	0.0077	0.0054	0.0089	109724	0.0063
Leu-Pro	5E+06	503317	5E+06	5E+06	5E+06	526797	2E+06	227496	2E+06	2E+06	2E+06	2E+06

Leu-Ser	0.0035	0.0027	0.0027	0.0018	0.0019	0.0015	0.0034	0.0029	0.0024	0.0026	0.0024	0.0019
Leu-Val	3E+06	3E+06	3E+06	2E+06	2E+06	2E+06	2E+06	2E+06	1E+06	309328	3E+06	2E+06
Glutamic acid	2E+06	194396	2E+06	185819	2E+06	2E+06	1E+06	14825	1E+06	1E+06	1E+06	1E+06
Glutamine	4E+06	3E+06	3E+06	3E+06	3E+06	3E+06	3E+06	3E+06	3E+06	4E+06	4E+06	4E+06
Histidine	6E+06	606445	6E+06	6E+06	6E+06	6E+06	8E+06	8E+06	8E+06	7E+06	675208	7E+06
Homoserine	115765	103389	0.0089	0.008	0.0083	0.0078	138193	22082	215478	149775	153448	100134
Hydroxyarginine	0.0066	0.006	136337	0.0059	0.0056	0.0059	0.0028	0.0025	0.0018	0.0025	0.0028	0.0028
Lisdexamfetamine	0.0042	0.0041	0.0043	0.0045	0.0041	0.0041	0.0072	0.0073	0.0071	0.0075	0.0073	0.0071
Isoleucine	6E+06	6E+06	7E+06	7E+06	6E+06	7E+06	7E+06	8E+06	8E+06	7E+06	7E+06	8E+06
Leucine	8E+06	9E+06	9E+06	9E+06	1E+07	9E+06	9E+06	1E+07	1E+07	9E+06	1E+07	1E+07
Lysine	4E+06	3E+06	4E+06	4E+06	4E+06	4E+06	5E+06	5E+06	460057	5E+06	5E+06	5E+06
Methionine	2E+06	6E+06	3E+06	2E+06	2E+06	2E+06	3E+06	4E+06	3E+06	3E+06	3E+06	3E+06
Phenylalanine, methyl ester	0.0088	0.0094	0.01	0.008	0.0095	0.0093	0.0067	0.0081	0.0065	0.006	0.007	0.0043
Pipecolic acid	438357	438323	420272	460104	420604	42116	251036	25426	245154	370436	439807	354509
Proline	3E+06	3E+06	3E+06	2E+06	2E+06	2E+06	8E+06	8E+06	7E+06	517002	5E+06	6E+06
Propionylcarnitine	4E+06	4E+06	4E+06	4E+06	4E+06	4E+06	3E+06	3E+06	3E+06	1E+06	1E+06	1E+06
Threonine	0.0014	0.0012	0.0011	0.0005	0.0001	0.0005	101335	117096	113621	0.0085	0.0077	0.0076
Tryptophan	5E+06	528774	5E+06	5E+06	5E+06	5E+06	57851	6E+06	6E+06	554449	6E+06	6E+06
Tyrosine	4E+06	51252	5E+06	5E+06	4E+06	5E+06	5E+06	6E+06	568914	5E+06	5E+06	6E+06
Valine	31933	3E+06	334537	308435	3E+06	3E+06	374631	4E+06	4E+06	4E+06	4E+06	4E+06
Lys-Asp	397683	425522	415259	324299	326231	325408	533367	535918	584462	534531	552469	582319
Lys-Pro	1E+06	114957	1E+06	107484	1E+06	1E+06	21327	130802	0.0098	212162	172277	126047
Lys-Ser	384814	346977	320721	236925	192166	184292	540345	464304	44478	557808	720188	477724
Maltotetraose	0.0013	0.0007	0.0004	0.0013	0.0013	0.0012	0.0006	0.0007	0.0004	0.0007	0.0006	0.0007
Melibiose	468569	293082	246489	271364	201538	272592	225992	338471	263481	225052	207293	221901
Met-Glu	0.0092	0.0083	0.0072	0.0041	0.0047	0.0038	0.0018	0.0011	0.0011	0.0033	0.0011	0.0001
Met-Gly	0.0045	0.0036	0.0035	0.0022	0.0022	0.0025	0.0019	0.0013	0.0011	0.003	0.0018	0.0015
Met-Pro	416868	371	418673	394672	434249	413411	195611	196428	194756	230789	269298	292612
N-(3-(Aminomethyl)benzyl)acetamidine	0.0022	0.0023	0.0025	0.0009	0.001	0.001	0.001	0.0011	0.001	0.0007	0.0011	0.0011
N-(3-Aminopropyl)acetamide	0.0075	0.007	0.0083	0.0049	0.0042	0.0047	0.0066	0.007	0.0069	120838	101753	131765
N-(4-Acetylphenyl)-4-methylbenzamide	0.009	0.0052	0.0054	0.0054	0.0056	0.0057	0.0047	0.0045	0.0046	0.0042	0.0042	0.0046
N-(Aminocarbonyl)phenylalanine	0.0014	0.0017	0.0017	0.0041	0.0046	0.0047	0.0032	0.0033	0.0033	0.0025	0.0027	0.003
N,N'-Diacetylchitobiose	0.0015	0.0004	0.0004	0.0003	0.0004	0.0003	0	0	0	0	0	0

N,N-Dimethylhistidine	0.0015	0.0011	0.0012	0.0021	0.0013	0.0019	0.0035	0.0033	0.0028	0.0017	0.0014	0.0011
N-α-(tert-Butoxycarbonyl)histidine	460049	456808	308037	421319	43752	465872	618931	616458	597027	484096	497878	507421
N-α-Acetylornithine	286184	218541	204033	185858	172559	170719	62317	556008	50158	430457	395701	354574
N-[2-(1H-Indol-3-yl)ethyl]nicotinamide	1E+06	2E+06	2E+06	665539	921403	1E+06	3E+06	4E+06	4E+06	5E+06	7E+06	8E+06
N1,N12-Diethylspermine	0.0009	0.0009	0.0008	0.0015	0.0014	0.0015	125248	126944	132328	119452	130937	124249
N5-(1-Iminoethyl)ornithine	2702	273933	2933	241766	273577	263414	191067	19633	184049	195599	198316	177758
N-Acetylglucosamine	116708	0.0063	0.0054	0.004	0.0034	0.004	0.0036	0.0063	0.0052	0.0055	0.0061	0.0062
1-Acetylproline	0.0009	0.0007	0.0006	0.0006	0.0008	0.0009	0.002	0.0025	0.0029	0.0044	0.0059	0.0068
N-Acetylphenylalanine	0.0012	0.0011	0.0011	0.0005	0.0007	0.0006	0.0025	0.0028	0.003	0.0019	0.0029	0.0029
N-Acetylproline	0.0018	0.0013	0.0011	0.001	0.001	0.001	0.0036	0.0034	0.0028	0.0026	0.0021	0.002
N-Acetyltyrosine	59344	656396	665971	605966	596093	638821	716527	76733	752499	666684	722306	760691
N-Cyclopentylglycine	0	0	0	0	0	0	0	0	0	158963	114516	10973
N-Formyltryptophan	3E+06	3E+06	4E+06	3E+06	3E+06	3E+06	4E+06	4E+06	4E+06	4E+06	373302	4E+06
NG,NG-Dimethylarginine	738279	643327	674284	5175	579017	582396	1E+06	1E+06	1E+06	986001	1E+06	995501
Nicotinic acid	2E+06	2E+06	3E+06	2E+06	2E+06	2E+06	1E+06	1E+06	891955	1E+06	969892	870127
Octanoylcarnitine	0.0056	0.0074	0.0078	0.0089	0.0085	0.0095	0.0067	0.007	0.0073	0.0032	0.0037	0.0035
O-tert-Butylthreonine	2E+06	1E+06	1E+06	1E+06	112495	1E+06	1E+06	1E+06	1E+06	951126	993011	1E+06
Pantothenic acid	652845	545193	512694	577072	610449	629698	6429	662902	67619	287369	0.01	187707
p-Coumaric acid	0.0072	0.0089	0.0088	0.0082	0.0078	0.0082	0.0094	0.0094	100427	0.0081	0.0095	0.0099
Phe-Gly	223116	22666	279775	257034	29659	290935	20657	235497	289744	205052	274979	272824
Phe-Pro	824426	1E+06	1E+06	916171	1E+06	1E+06	560212	557976	555693	46197	557296	421516
Phosphocholine	737865	312945	274334	332578	27407	318794	547853	610662	536994	435219	463029	391621
Phthalic acid	223567	211997	120631	313627	136506	13869	2661	129389	12218	314564	127375	128538
Pro-Ala	199952	162866	190542	33404	389076	422344	390875	421665	499244	298401	298502	460346
Pro-Ala-Arg	0.0006	0.0007	0.0008	0.0007	0.0009	0.0009	0.0029	0.003	0.001	0.002	0.002	0.0008
Pro-Asn	0.0095	0.0061	0.0056	0.0074	0.0062	0.0057	0.0091	0.0061	0.0051	0.0079	0.0057	0.0055
Pro-Asp	142673	121224	126311	110967	115753	124966	152824	134773	142377	129436	138751	152455
Pro-Gln	200844	171013	176238	179555	184051	196775	265717	230548	243429	278304	271866	234912
Pro-Glu	272415	22145	22099	264118	276482	27079	354696	300598	308218	242289	212512	298714
Pro-Gly	192238	189399	187045	162716	158266	188221	272496	231398	212028	215521	21673	210994
Prolylalanine	0.0025	0.003	0.0023	0.0068	0.0074	0.0072	0.0077	0.0023	0.0024	0.0027	0.0031	0.0033
Pro-Phe	204867	242153	284783	440734	489888	552511	764537	87117	908751	894572	875635	925374
Pro-Pro	2E+06	2E+06	213569	2E+06	2E+06	3E+06	309478	3E+06	3E+06	3E+06	3E+06	3E+06

Pro-Thr	22045	198299	17902	249578	218717	219206	420416	356077	327825	321063	293544	290902
Pro-Tyr	0.0034	0.003	0.0034	0.0095	0.0096	103367	142115	135464	143735	186778	167692	199354
Pro-Val	216706	241708	26246	314159	321172	407215	1E+06	2E+06	2E+06	1E+06	1E+06	2E+06
Pyrrolidine	0.002	0.0022	0.0027	0.0053	0.0058	0.0056	195021	190296	211994	180094	192912	18469
Acetamidomethylcysteine	0.0014	0.0009	0.0009	0.0009	0.0008	0.0007	0.0045	0.006	0.0073	0.0043	0.0059	0.0097
Sarcosine	114323	146863	0.0095	0.0099	0.0095	10259	0.0071	0.0065	0.0065	0.0068	0.0068	0.0062
Ser-Glu	337435	263965	233733	168218	158333	14683	252796	178293	140891	190026	143836	134413
Ser-Ile	207595	200661	218973	13911	16863	150527	211804	182477	175143	263406	264209	24422
Ser-Leu	459529	473836	572328	254625	344333	326204	38489	375908	386721	417552	471574	404945
Ser-Lys	242645	250206	208981	172508	152061	151967	169376	147303	159593	113208	153799	155603
Ser-Pro	799044	710294	63038	564749	506041	536689	131801	0.005	0.0055	0.0092	0.0065	0.0044
Ser-Thr	401174	409832	367782	274406	258298	267081	353121	353661	331871	299454	298294	285544
Spermidine	438983	397751	430618	826267	830403	810078	7E+06	6E+06	7E+06	6E+06	670365	6E+06
Tetrabutylammonium cation	1E+06	9E+06	9E+06	8E+06	792066	8E+06	8E+06	8E+06	7E+06	8E+06	7E+06	7E+06
Thiamine cation	0.0029	0.0025	0.0024	0.0024	0.0023	0.002	0.0083	0.0069	0.0059	0.0063	0.0065	0.0062
Thr-Ala	518572	407481	373825	279068	275258	265234	410996	342291	279569	256716	229686	215214
Thr-Arg	758832	72378	770463	31748	296728	275123	646596	686406	66043	475029	494712	48325
Thr-Glu	459799	439172	396435	261859	26607	297694	281056	222108	173227	204057	167699	172742
Thr-Gly	109287	162643	233179	121163	151711	177382	120409	202467	241752	110679	16954	197707
Thr-Leu	649341	539646	515463	423242	4948	444471	638349	559969	499607	843991	709822	625731
Thr-Lys	400137	383125	368561	262921	259896	270989	554776	556984	521696	50615	561699	478146
Thr-Pro	2E+06	2E+06	2E+06	1E+06	1E+06	1E+06	415993	33354	304625	280617	271203	166423
Thr-Ser	175671	159436	151103	106004	11271	113961	116204	110701	0.0094	0.007	0.0069	0.007
Thr-Thr	357789	23455	200857	152509	140409	117036	315254	226144	183088	21871	168836	141873
Thr-Trp	0.0097	0.0085	0.0082	0.0074	0.0087	0.0081	311086	27845	247758	366419	329823	284559
trans-Aconitic acid	0.001	0.0007	0.0005	0.0007	0.0005	0.0006	0.0007	0.0007	0.0005	0.0023	0.0027	0.0019
trans-Cinnamic acid	151382	164685	167889	150601	15701	156768	190693	198001	19427	18951	194821	195212
Tropic acid	12822	138933	141466	125321	128765	134866	155773	167053	160034	154668	163354	165583
Tryptamine	394392	62676	884996	58653	790492	849763	1E+06	2E+06	2E+06	3E+06	4E+06	5E+06
Tyr-Glu	213179	213408	167255	219865	182119	17712	0.0014	0.0015	0.001	0.0022	0.0015	0.001
Tyr-Pro	384145	413475	458586	382066	481988	411219	160403	159043	150822	152629	164621	110121
Urea	0	0	0	0	0	0	0.0022	0.0022	0.0024	0.0018	0.0018	0.0024
Val-Ala	1E+06	846361	911813	399776	434308	422424	1E+06	1E+06	1E+06	1E+06	1E+06	907749

Val-Arg	677944	499181	382804	665633	576238	465956	1E+06	1E+06	783222	1E+06	1E+06	765002
Val-Asn	53805	424425	399752	271512	252579	248957	658085	551195	489945	579403	569007	432488
Val-Asp	178707	140302	148273	113986	118816	118033	249225	222823	181728	170172	164536	146246
Val-Gln	98739	75281	755651	539915	558034	476935	970289	796601	658803	684487	635016	484499
Val-Glu	528638	461004	457731	2867	29371	280979	527745	447495	395025	498387	45584	408566
Val-His	32337	272398	25786	280721	289496	254286	636277	539305	439723	603856	610824	50855
Val-Ile	108698	108233	1E+06	723156	842537	741652	2E+06	2E+06	2E+06	2E+06	2E+06	2E+06
Val-Lys	1E+06	1E+06	1E+06	1E+06	1E+06	1E+06	2E+06	2E+06	2E+06	2E+06	228259	2E+06
Val-Met	138544	100111	0.0088	0.0086	0.0099	0.0081	207136	189003	206676	331177	330476	282829
Val-Phe	529203	399431	31504	449553	475433	356427	544919	486004	34586	917194	64858	429862
Val-Pro	3E+06	3E+06	3E+06	2E+06	3E+06	3E+06	758415	698766	63598	61399	690323	391455
Val-Ser	0.0062	0.0044	0.0075	0.002	0.0041	0.0046	0.0075	0.0096	166693	0.0038	0.0055	0.0086
Val-Thr	749752	562372	548205	372922	388147	314427	714472	574184	4724	557115	476139	3662
Val-Trp	101281	0.0067	0.0032	0.0061	0.0067	0.0052	12112	137365	102849	268343	264691	154206
Val-Val	714652	740127	826526	531339	720439	663026	2E+06	2E+06	2E+06	2E+06	257803	2E+06
Vildagliptin	111629	11891	123324	120044	35332	123046	118859	121095	116821	108981	10639	112303

Figure 1S. Fragmentation patterns for the identification of three selected amino acids and dipeptides (blue colour) confirmed by comparison with theoretical spectra (red colour).

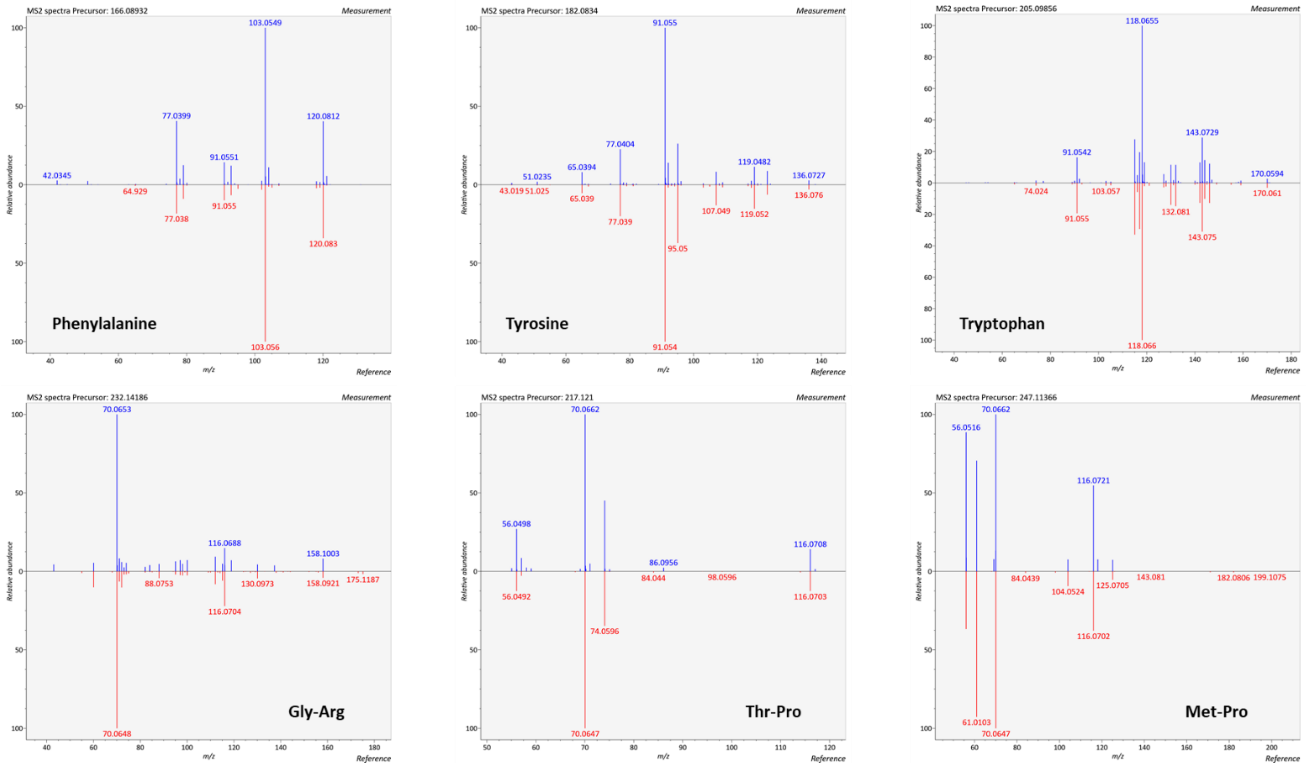

Table 3S. Enrichment pathways of 24 discriminant amino acids differentially expressed in *P. ostreatus* samples grown on WS and LcS substrates: Match Status is the number of metabolites that match in the indicated pathway; p is the original p-value calculated from the Enrichment Analysis; Holm p value is the p value adjusted by Holm-Bonferroni test; impact is the pathway impact value calculated from pathway topology analysis.

Pathway Name	Match Status	*p*	-log(p)	Holm p	Impact	Involved metabolites
Glycine, serine and threonine metabolism	2/33	6.72E-09	8.1727	1.28E-07	0.05034	Betaine, Threonine
Valine, leucine and isoleucine biosynthesis	1/8	1.18E-06	5.9282	2.12E-05	0	Threonine
D-Glutamine and D-glutamate metabolism	1/6	1.18E-05	4.9283	2.01E-04	0.5	Glutamate
Glutathione metabolism	1/28	1.18E-05	4.9283	2.01E-04	0.01966	Glutamate
Glyoxylate and dicarboxylate metabolism	1/32	1.18E-05	4.9283	2.01E-04	0	Glutamate
Butanoate metabolism	1/15	1.18E-05	4.9283	2.01E-04	0	Glutamate
Porphyrin and chlorophyll metabolism	1/30	1.18E-05	4.9283	2.01E-04	0	Glutamate
Nitrogen metabolism	1/6	1.18E-05	4.9283	2.01E-04	0	Glutamate
Arginine and proline metabolism	2/38	1.47E-05	4.8322	2.01E-04	0.1638	Proline, Glutamate
Alanine, aspartate and glutamate metabolism	2/28	1.97E-05	4.7064	2.01E-04	0.42068	Aspartate, Glutamate
Arginine biosynthesis	2/14	1.97E-05	4.7064	2.01E-04	0.11675	Aspartate, Glutamate
Histidine metabolism	2/16	1.97E-05	4.7064	2.01E-04	0	Aspartate, Glutamate
Aminoacyl-tRNA biosynthesis	5/48	4.37E-05	4.3596	3.06E-04	0	Phenylalanine, Aspartate, Threonine,Proline, Glutamate
Lysine degradation	1/25	1.17E-04	3.9327	7.01E-04	0	Pipecolate
beta-Alanine metabolism	1/21	0.001542	2.8119	0.0077099	0	Aspartate
Nicotinate and nicotinamide metabolism	1/15	0.001542	2.8119	0.0077099	0	Aspartate
Pantothenate and CoA biosynthesis	1/19	0.001542	2.8119	0.0077099	0	Aspartate
Phenylalanine, tyrosine and tryptophan biosynthesis	1/4	0.082706	1.0825	0.16541	0.5	Phenylalanine
Phenylalanine metabolism	1/10	0.082706	1.0825	0.16541	0.35714	Phenylalanine
